# Synthesis, Biological Evaluation and In Silico Studies of Certain Oxindole–Indole Conjugates as Anticancer CDK Inhibitors

**DOI:** 10.3390/molecules25092031

**Published:** 2020-04-27

**Authors:** Tarfah Al-Warhi, Ahmed M. El Kerdawy, Nada Aljaeed, Omnia E. Ismael, Rezk R. Ayyad, Wagdy M. Eldehna, Hatem A. Abdel-Aziz, Ghada H. Al-Ansary

**Affiliations:** 1 Department of Chemistry, College of Science, Princess Nourah bint Abdulrahman University, Riyadh 12271, Saudi Arabia; tarfah-w@hotmail.com (T.A.-W.); noaljaeed@pnu.edu.sa (N.A.); 2 Department of Pharmaceutical Chemistry, Faculty of Pharmacy, Cairo University, Kasr El-Aini Street, Cairo 11562, Egypt; ahmed.elkerdawy@cu.edu.eg; 3 Department of Pharmaceutical Chemistry, Faculty of Pharmacy, New Giza University, Newgiza, km 22 Cairo–Alexandria Desert Road, Cairo 12577, Egypt; 4 Department of Biochemistry, Faculty of Pharmacy, Egyptian Russian University, Badr City, Cairo 11829, Egypt; omniaezzat@gmail.com; 5 Department of Pharmaceutical Chemistry, Faculty of Pharmacy, Al-Azhar University, Cairo 11651, Egypt; rezek_ayad@yahoo.com; 6 Department of Pharmaceutical Chemistry, Faculty of Pharmacy, Kafrelsheikh University, Kafrelsheikh 33516, Egypt; 7 Department of Applied Organic Chemistry, National Research Center, Dokki, Giza 12622, Egypt; hatem_741@yahoo.com; 8 Department of Pharmaceutical Chemistry, Pharmacy Program, Batterejee Medical College, Jeddah 6231, Saudi Arabia; 9 Department of Pharmaceutical Chemistry, Faculty of Pharmacy, Ain Shams University, Abbassia, Cairo 11566, Egypt

**Keywords:** anti-proliferative activity, apoptosis, breast cancer, oxindole, CDK4 inhibitors

## Abstract

On account of their overexpression in a wide range of human malignancies, cyclin-dependent kinases (CDKs) are among the most validated cancer targets, and their inhibition has been featured as a valuable strategy for anticancer drug discovery. In this study, a hybrid pharmacophore approach was adopted to develop two series of oxindole–indole conjugates (**6a–i** and **9a–f**) and carbocycle–indole conjugates (**11a**,**b)** as efficient antitumor agents with potential inhibitory action toward CDK4. All oxindole–indole conjugates, except **6i**, **9b**, and **9c** efficiently affected the growth of the human breast cancer MCF-7 (IC_50_: 0.39 ± 0.05–21.40 ± 1.58 μM) and/or MDA-MB-231 (IC_50_: 1.03 ± 0.04–22.54 ± 1.67 μM) cell lines, whereas bioisosteric replacement of the oxindole nucleus with indane or tetralin rings (compounds **11a**,**b**) diminished the anti-proliferative activity. In addition, hybrids **6e** and **6f** displayed effective cell cycle disturbance and proapoptotic capabilities in MCF-7 cells. Furthermore, the efficient anti-proliferative agents towards MCF-7 and/or MDA-MB-231 cell lines (**6a–h**, **9a**, and **9e**) were investigated for their potential inhibitory action toward CDK4. Hybrids **6a** and **6e** displayed good CDK4 inhibitory activity with IC_50_s equal 1.82 and 1.26 µM, respectively. The molecular docking study revealed that oxindole moiety is implicated in two H-bonding interactions via both (NH) and (C=O) groups with the key amino acids Glu94 and Val96, respectively, whereas the indole framework is stably accommodated in a hydrophobic sub-pocket establishing hydrophobic interactions with the amino acid residues of Ile12, Val20, and Gln98 lining this sub-pocket. Collectively, these results highlighted hybrids **6a** and **6e** as good leads for further optimization as promising antitumor drugs toward breast malignancy and CDK inhibitors.

## 1. Introduction 

Cancer is a serious health problem that is considered a leading cause of death worldwide. Cancer is characterized by uncontrolled cell proliferation and differentiation mechanisms [1]. Although it started long ago, the search for effective and safer new antitumor drugs is yet an active research topic [2]. This is attributed to the systemic toxicity caused by the non-selective traditional cytotoxic chemotherapies and their side effects, in addition to the resistance emerged toward them [2]. On the other hand, targeted therapies selectively target cancer cells or their supporting environment with minimum effects on normal cells [3,4].

Cyclin-dependent kinases (CDKs) are categorized as protein serine/threonine kinases that are responsible for regulating the cell cycle through the complexation with regulatory proteins known as “cyclins”. These complexes are responsible for the progression of the cell cycle through its different phases [5,6]. This key role of CDKs in cell cycle progression makes them potential targets for cancer treatment and makes their inhibitors promising for targeted chemotherapy [7,8,9,10]. 

**Figure 1:**
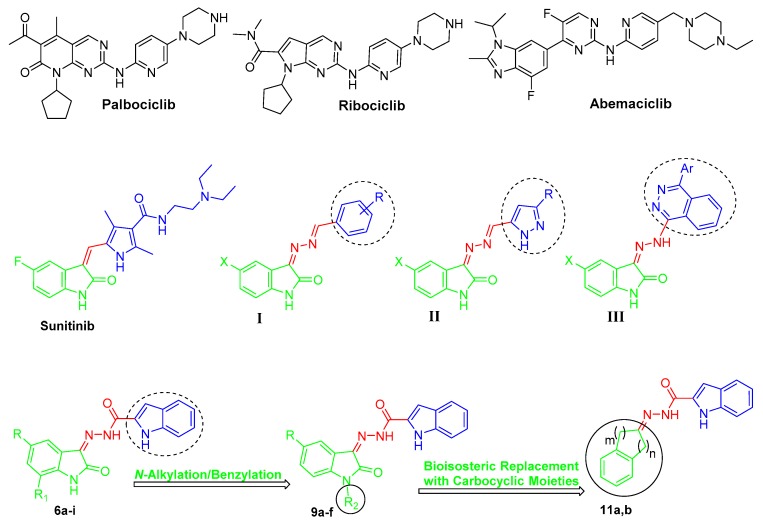
Chemical structures for the approved CDK4/6 inhibitors (palbociclib, ribociclib, and abemaciclib), approved oxindole-based anticancer drug (sunitinib), some reported oxindole-based anticancer hybrids (**I–III**), and the target hybrids (**6a–i**, **9a–f**, and **11a**,**b**).

CDK4 is a key CDK cell cycle regulator that is responsible for the progression from the G1 to S phase through complexation with cyclin-D at the G1-phase [11,12]. Several cancer types such as non-small cell lung cancer, colorectal carcinomas, melanomas, and breast cancer involve decreased levels of endogenous CDK inhibitors, as well as CDK4 overexpression [13,14]. Moreover, CDK4 cognate cyclin “cyclin D1” is commonly upregulated in several cancer types such as breast, lung, bladder, and GIT cancers [15]. Therefore, CDK4 inhibition is featured as a potential strategy for targeted treatment of several cancers through cell cycle arrest within the G1 phase [16]. Palbociclib, ribociclib, and abemaciclib (Figure 1) are recently approved CDK4/6 inhibitors for advanced or metastatic breast cancer treatment [17,18,19].

As a privileged scaffold, isatin (indole-2,3-dione) represents a leading and promising heterocyclic nucleus that is endowed with a range of interesting biological properties, chiefly antitumor activities [20]. Upon FDA approval in 2006 of the anticancer drug sunitinib (Sutent^®^, Figure 1), a multikinase oxindole-based inhibitor, for gastrointestinal stromal tumor as well as renal cell carcinoma [21], the isatin nucleus was extensively utilized for the development of diverse effective small molecules with efficient anticancer activities. In this vein, diverse oxindole-based derivatives were designed and synthesized as potential inhibitors for several tyrosine and serine/threonine kinases such as CDKs [22], FLT3 kinase [23], polo-like kinase 4 (PLK4) [24], glycogen synthase kinase-3*β* (GSK-3*β*) [25], aurora B kinase [26], p90 ribosomal S6 protein kinase 2 (RSK2) [27], and microtubule affinity-regulating kinase 4 (MARK4) [28], to name just a few.

In the current medical era, the molecular hybridization strategy is being featured as a promising drug design approach, particularly in anticancer drug discovery [29,30]. It is thought that conjugating two or more pharmacophoric subunits from different biologically active molecules in the molecular architecture of a single hybrid compound might reduce the risk of drug–drug interactions, overcome the drug resistance, and enhance the biological activity via the potential interaction with two targets as one single entity [31]. In this field, our research group has reported many studies concerning the development of efficient oxindole-based anticancer hybrids (hybrids **I**–**III** [32,33,34], Figure 1) that exhibited different enzymatic and cellular targets such as apoptosis induction in different human cancer cell lines [35,36], inhibition of cancer-related carbonic anhydrase IX isoform [37,38], in addition to inhibition of tyrosine kinases [39,40].

Prompted by the above-mentioned findings, in this study we adopted a hybrid pharmacophore approach to develop different series of oxindole/carbocycle–indole conjugates (**6a–i**, **9a–f**, and **11a**,**b)** aiming to develop efficient antitumor agents with potential inhibitory action toward CDK4. First, the privileged indole moiety was selected to be conjugated with the isatin scaffold to furnish the first series (**6a–i**); thereafter, N-1 of the oxindole moiety was alkylated and benzylated to produce the second set of target hybrids (**9a–f**) (Figure 1). Finally, a bioisosteric replacement approach was carried out by replacing the oxindole moiety with another carbocyclic moieties, indane and tetralin rings, to yield the third set (**11a**,**b**) with a view to investigating the significance of the oxindole moiety in achieving the desired biological activities (Figure 1). 

All the reported conjugates (**6a–i, 9a–f**, and **11a,b**) were examined for their potential cytotoxic activity towards MCF-7 and MDA-MB-231 cancer cell lines via SRB assay. Thereafter, hybrids **6e** and **6f** were further explored for their plausible influence on cell cycle progression and apoptosis induction possibility in breast cancer MCF-7 cells. In addition, the most efficient anti-proliferative agents towards MCF-7 and/or MDA-MB-231 cells (conjugates **6a–h**, **9a**, and **9e**) were examined for their inhibitory action toward CDK4, and then docked in the CDK4 binding site to justify their inhibitory action as well as to explore their binding pattern.

## 2. Results and Discussion 

### 2.1. Chemistry

**Scheme 1:**
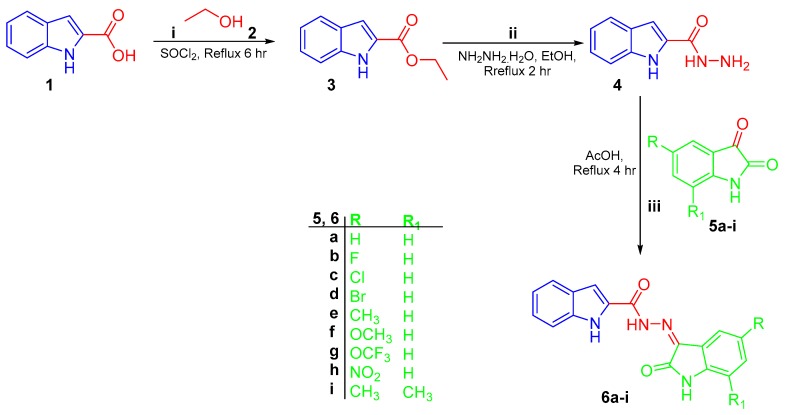
Synthesis of conjugates **6a–i**; (**i**) SOCl_2_/reflux 6 h; (**ii**), (**iii**) 99% NH_2_NH_2_.H_2_O/ethanol/reflux 2 h; (**iii**) glacial acetic acid/reflux 4 h.

**Scheme 2:**
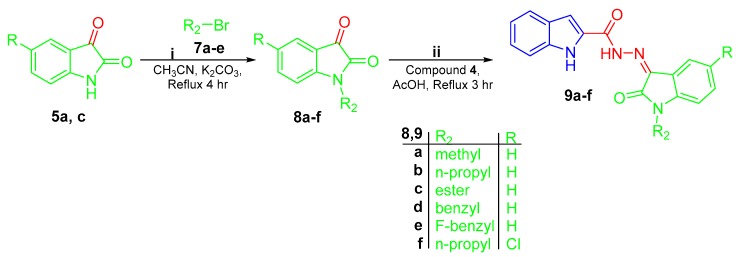
Synthesis of conjugates **9a–f**; (**i**) acetonitrile/K_2_CO_3_/reflux 4 h; (**ii**) 1*H*-indole-2-carbohydrazide **4/**glacial acetic acid/reflux 3 h.

**Scheme 3:**
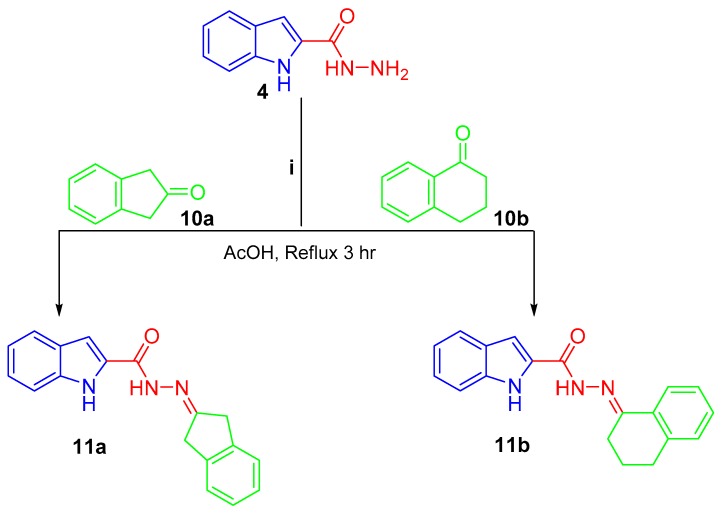
Synthesis of conjugates **11a, b**; (**i**) glacial acetic acid/reflux 3 h.

Preparation of targeted conjugates **6a–i, 9a–f**, and **11a,b** was performed according to the general synthetic tracks as shown in Scheme 1, Scheme 2 and Scheme 3. In the first scheme, esterification of 1*H*-indole-2-carboxylic acid **1** was performed through refluxing in absolute ethanol in the presence of thionyl chloride to produce ethyl 1*H*-indole-2-carboxylate **3** [41], which was subsequently treated by hydrazine monohydrate (N_2_H_4_·H_2_O) in boiling ethanol to produce the key intermediate 1*H*-indole-2-carbohydrazide **4**. Final target hybrids **6a–i** were obtained via condensation of 1*H*-indole-2-carbohydrazide **4** with the appropriate unsubstituted (**5a**), 5-substituted (**5b–h**), and 5,7-disubstituted (**5i**) isatin derivative in refluxing glacial acetic acid for four hours (Scheme 1).

In Scheme 2, *N*-substituted isatins **8a–f** were prepared through *N*-alkylation of isatins **5a** and **5c** with different alkyl halides **7a–e** in boiling acetonitrile in the presence of K_2_CO_3_. Thereafter, the produced *N*-substituted isatins **8a–f** were condensed with the key intermediate 1*H*-indole-2-carbohydrazide **4** in refluxing glacial acetic acid in order to yield the target hybrids **9a–f** (Scheme 2).

Lastly, treating the hydrazide **4** with 2-indanone **10a** and 1-tetralone **10b** in refluxing glacial acetic acid afforded the final compounds **11a** and **11b**, respectively (Scheme 3).

The proposed structures for the target conjugates **6a–i**, **9a–f**, and **11a**,**b** were in full accordance with their spectral and elemental analyses data.

### 2.2. Biological Evaluation 

#### 2.2.1. Anti-Proliferative Activities towards Breast Cancer Cell Lines (MCF-7 and MDA-MB-231)

All the synthesized conjugates (**6a–i, 9a–f**, and **11a,b**) were examined for the potential cytotoxic activity towards breast cancer MCF-7 and MDA-MB-231 cell lines, utilizing the protocol of the sulforhodamine B colorimetric (SRB) assay [42]. With the aim of extracting a reasonable structure–activity relationship (SAR), three series: **6**, **9**, and **11** were designed with diverse substituents on C-5 and C-7 of the oxindole moiety; R and R_1_ in series **6a–i**, and diverse substitutions on the N-1 of the oxindole moiety; and R_2_ in series **9a–f**, while the oxindole moiety was replaced by an indane and tetralin rings in compounds **11a**,**b** in order to explore the privilege of the oxindole moiety over other pharmacophoric groups. The IC_50_ values for the tested conjugates were compared as an insight of their anti-proliferative potency taking the clinically approved staurosporine as the positive control anticancer drug (Table 1).

Analyzing the obtained IC_50_ values for the tested hybrids toward MCF-7 breast cancer cells revealed that most hybrids belonging to series **6** had excellent activity against the aforementioned cell line with IC_50_ values spanning between 0.39 ± 0.05 μM and 6.00 ± 0.32 μM, with the exception of compounds **6h** and **6i** (IC_50_ = 21.4 ± 1.58 and 47.16 ± 3.02 μM, respectively) bearing a strong electron withdrawing 5-NO_2_ group and 5,7-dimethyl substitution on the oxindole moiety, respectively (Table 1).

The IC_50_ of the unsubstituted derivative **6a** (IC_50_ = 3.12 ± 0.14 μM) revealed that it had double the activity of staurosporine (IC_50_ = 6.81 ± 0.22 μM), which highlighted that this scaffold is a promising one. The influence of grafting different groups on the oxindole moiety reflected that substitution with a halide was advantageous for the anti-proliferative activity in case of 5-chloro substituent in **6c**, as it displayed slight potency enhancement compared to its unsubstituted analogue **6a** (IC_50_ values 2.72 ± 0.17 μM vs. 3.12 ± 0.14 μM, respectively), while the 5-bromo derivative **6d** (IC_50_ = 3.31 ± 0.11 μM) did not prove to be more effective than the unsubstituted derivative **6a** (IC_50_ = 3.12 ± 0.14 μM), and even the 5-fluoro derivative **6b** (IC_50_ = 6.00 ± 0.32 μM) showed about half the activity in comparison to **6a** (IC_50_ = 3.12 ± 0.14 μM).

Furthermore, C-5 monosubstitution of the oxindole motif with a methyl group in **6e** (IC_50_ = 0.39 ± 0.05 μM) markedly boosted the activity 8-fold compared to the unsubstituted prototype **6a** (IC_50_ = 3.12 ± 0.14 μM), emphasizing that an electron donating group is advantageous for activity. In accordance with this finding, compound **6f**, bearing an electron-donating 5-OCH_3_ group, also exhibited excellent anti-proliferative activity (IC_50_ = 1.85 ± 0.08 μM) with 1.7-fold efficacy enhancement as compared to **6a** (IC_50_ = 3.12 ± 0.14 μM). Unfortunately, compound **6g**, which possesses a trifluoromethoxy group, did not exhibit superior anti-proliferative activity compared to **6a**, yet it still proved to be more efficient than the reference compound, staurosporine, with an IC_50_ value of 5.07 ± 0.21 μM.

On the other hand, the tested hybrids of series **9** did not show better cytotoxic action against the MCF-7 cell line than their *N*-unsubstituted analogues **6a–i**; (compounds **9a–e**; IC_50_: 3.70 ± 0.19–67.51 ± 3.02 μM vs. compound **6a**; IC_50_ = 3.12 ± 0.14 μM) and (compound **9f**; IC_50_ = 19.26 ± 0.65 μM vs. compound **6c**; IC_50_ = 2.72 ± 0.17 μM), indicating that both *N*-alkylation and *N*-benzylation for the oxindole nucleus is not advantageous for the growth inhibitory action toward MCF-7 breast cancer cell line. N-1 substitution of the oxindole moiety with a methyl group produced compound **9a**, which is slightly more potent toward the MCF-7 cell line compared to the reference staurosporine (IC_50_ = 5.50 ± 0.28 μM vs. 6.81 ± 0.22 μM, respectively). Alternatively, compound **9b**, bearing an n-propyl group, and **9c**, possessing an ester group, showed very poor anti-proliferative action against MCF-7 cancer cells with IC_50_s equal to 40.89 ± 3.17 μM and 67.51 ± 3.02 μM, respectively. While compound **9d**, bearing a benzyl ring, showed moderate anti-proliferative activity with an IC_50_ value of 9.56 ± 0.61 μM, compound **9e**, with a fluorobenzyl group, proved to be three-fold more potent compared to its counterpart **9d** with an unsubstituted benzyl group with an IC_50_ of 3.70 ± 0.19 μM, Table 1. 

Moreover, the 5-chlorooxindole-based hybrid **9f**, bearing an n-propyl group, displayed about two-fold enhanced activity (IC_50_ = 19.26 ± 0.65 μM) toward the MCF-7 cell line than its C-5 unsubstituted counterpart **9b** (IC_50_ = 40.89 ± 3.17 μM), highlighting that C-5 substitution may be beneficial for the growth inhibitory activity of the *N*-substituted oxindole-based hybrids against MCF-7 cells. Moreover, it indicates that a chloro substitution at the oxindole C-5 is beneficial for activity, as also seen in compound **6c**.

Scrutinizing the IC_50_ profile for the tested hybrids against the MDA-MB-231 cell line unravels very interesting results. All the compounds showed a narrower range of IC_50_ values against MDA-MB-231 as compared to their corresponding values toward the MCF-7 cell line. The IC_50_s were ranging from 1.03 ± 0.04 μM to 22.54 ± 1.67 μM, which proves that the examined conjugates exhibited more potent growth inhibitory action against TNBC, MDA-MB-231, with the exception of conjugates **6i**, **9c**, **11a**, and **11b**, which showed no activity toward the tested cell line. Regarding the IC_50_ values of series **6a–i**, the unsubstituted derivative, **6a**, did not show superior activity to staurosporine (IC_50_ values 15.10 ± 0.73 μM vs. 10.29 ± 0.72 μM, respectively), whereas C-5 substitution of the oxindole nucleus with a halide was suggested to be advantageous for the anti-proliferative action regarding the fluoro substitution in **6b** (IC_50_ = 6.36 ± 0.29 μM), which is more potent than the reference compound (IC_50_ = 10.29 ± 0.72 μM). Moreover, grafting a chloro group afforded **6c** with comparable potency to staurosporine (IC_50_ values 9.48 ± 0.44 μM vs. 10.29 ± 0.72 μM), while substitution with a bromo group afforded **6d** (IC_50_ = 18.33 ± 0.71 μM), which is much less potent than staurosporine. This proves that increasing the size of the halide group is detrimental for the cytotoxic activity against MDA-MB-231. Unexpectedly, grafting a methyl group was not advantageous for the growth inhibitory action toward breast cancer MDA-MB-231 cells in contrast to activity recorded against MCF-7, as the IC_50_ of **6e** against MDA-MB-231 was 22.54 ± 1.67 μM, which is almost half the potency of staurosporine. Fortunately, grafting a stronger electron donating group, a methoxy group, boosted the activity of **6f** (IC_50_ = 2.85 ± 0.9 μM) 3.6-fold as compared to staurosporine (IC_50_ = 10.29 ± 0.72 μM). In addition, substitution with a trifluoromethoxy group imparted **6g**, which is more potent than the reference drug (IC_50_ values 7.19 ± 0.38 μM vs. 10.29 ± 0.72 μM). Surprisingly, grafting a strong electron withdrawing group, a nitro group, afforded **6h**, which is 5.56-fold more potent compared to staurosporine (Table 1). 

Regarding the antiproliferative action of series **9a–f** against the MDA-MB-231 cell line, all the tested conjugates displayed excellent to moderate activity except for **9c**, which had no anti-proliferative activity. *N*-substitution of the oxindole moiety with a methyl group afforded **9a**, which is more potent than staurosporine (IC_50_ values 8.39 ± 0.27 μM vs. 10.29 ± 0.72 μM). Increasing the alkyl group to a propyl group afforded **9b** which has a greater IC_50_ value (18.25 ± 0.85 μM), suggesting that increasing the bulkiness of the alkyl group is unfavorable for the anti-proliferative activity. Conversely, substitution with a benzyl group afforded **9d**, which exhibited good anti-proliferative activity in comparison to staurosporine (IC_50_ values 6.50 ± 0.39 μM vs. 10.29 ± 0.72 μM). Replacement of the benzyl ring with a fluorobenzyl group even boosted the activity ten-fold as compared to staurosporine (IC_50_ values 1.03 ± 0.04 μM vs. 10.29 ± 0.72 μM).

Finally, bioisosteric replacement of the oxindole moiety with indane and tetralin carbocyclic rings afforded carbocycle–indole conjugates (**11a**,**b)**, which did not exert any significant anti-proliferative action toward MCF-7 and MDA-MB-231 cancer cell lines (Table 1), emphasizing the importance of the oxindole motif as a privileged pharmacophoric group for the desired anticancer activity.

#### 2.2.2. Cell Cycle Analysis

**Figure 2:**
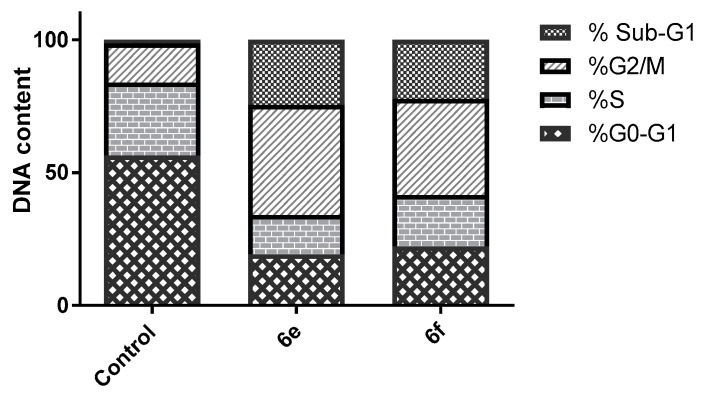
Impact of conjugates **6e** and **6f** on the cell cycle phases of MCF-7 cells.

Most of the cytotoxic agents wield their anti-proliferative effect by arresting the cell cycle at some checkpoints. Cell cycle analysis employs flow cytometry to distinguish cells within the different cell cycle phases. In this work we inspected the effect of two of the most potent compounds, **6e** and **6f**, on the cell cycle progression in order to explore the definite phase at which cell cycle arrest takes place in the MCF-7 breast cancer cell line. MCF-7 cells were treated by the IC_50_ of the two conjugates and their impact on the cell population was recorded. Treatment of MCF-7 cells with **6e** and **6f** resulted in significant decline in the cell population at the G0/G1 phase with 19.23% and 22.19%, respectively, compared to that of the control, which was 56.45% (Table 2, Figure 2). This was accompanied by discernible escalation in the proportion of cells in the G2/M phase by 2.80- and 2.46-fold with concomitant elevation in the sub-G1 phase by 17.8 and 16.25-fold, respectively, in comparison to the control (DMSO). This obviously indicates that our compounds halted the cell cycle proliferation of MCF-7 cells in the G0/G1 phase.

#### 2.2.3. The Effect on the Apoptotic and Anti-Apoptotic Marker Levels

**Figure 3:**
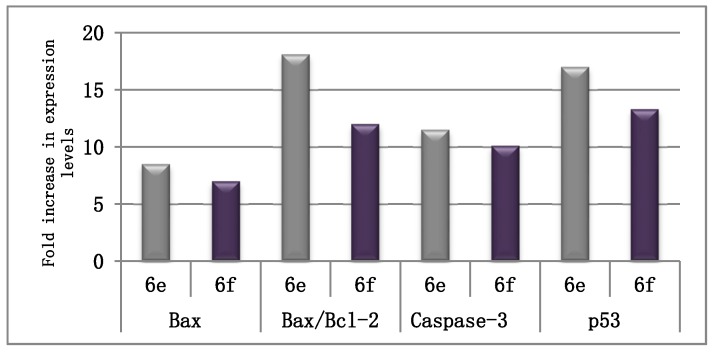
The extent of fold increase on the Bax/Bcl-2 ratio, in addition to expression levels of Bax, caspase-3, and p53 in MCF-7 cells after treatment with compounds **6e** and **6f**, as compared to the control (1% DMSO).

The influence of conjugates **6e** and **6f** on the expression levels of the distinguishable apoptotic markers (Bax, caspase-3, and p53) and the anti-apoptotic marker Bcl-2 was investigated to prove that our tested compounds exert their antiproliferative activity by driving the cell to apoptosis. Auspiciously, both conjugates evidently enhanced the level of the proapoptotic Bax protein by 8.5- and 7-fold, respectively. Homogeneously, both conjugates were able to decrease the level of the anti-apoptotic Bcl-2 protein by 2.12-, and 1.7-fold, respectively (Table 3). As a more analytical variable, the Bax/Bcl-2 ratio was verified to be amplified by conjugates **6e** and **6f,** 18- and 12-fold, respectively. This further shows that these compounds prompt apoptosis by considerably augmenting the Bax/Bcl-2 ratio (Table 3, Figure 3).

In addition, their influence on the level of caspase-3 and p53 was estimated in the MCF-7 cell line. The results revealed that **6e** and **6f** boosted the expression level of caspase-3 by 11.45- and 10.1-fold, respectively, in comparison to the control (Table 4, Figure 3). Moreover, they increased the level of p53 by 17- and 13.3-fold, respectively, as compared to the control. These results indicate that our compounds induce apoptosis, resulting in their anti-proliferative activity.

#### 2.2.4. Annexin V-FITC (Anx V) Apoptosis Assay

Anx V-based flow cytometry assay represents a helpful tactic for determining whether death of cells is attributed to programmed apoptosis or to uncontrolled necrosis.

**Figure 4:**
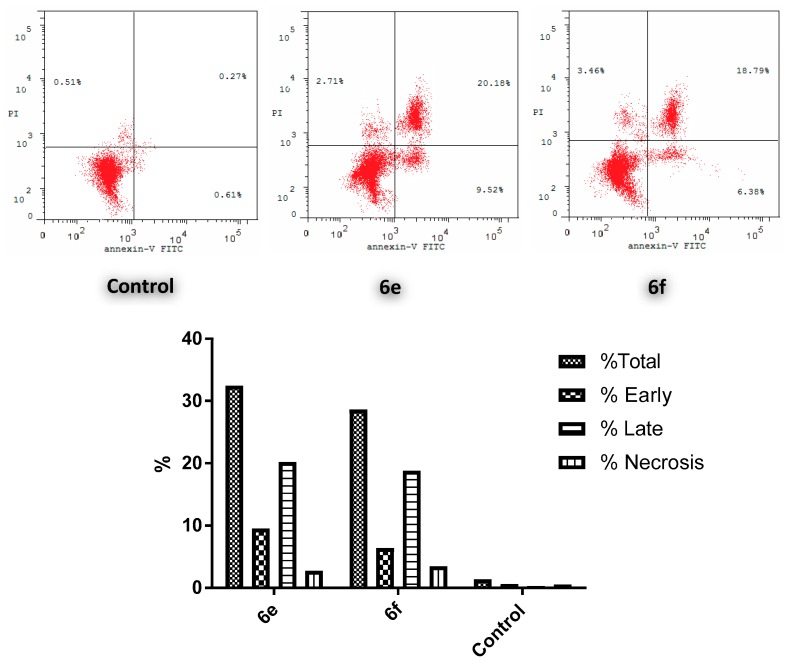
Influence of **6e** and **6f** on the percentage of annexin V-FITC-positive staining in MCF-7 cells.

Since conjugates **6e** and **6f** showed the highest anticancer action toward the MCF-7 cell line, both conjugates were selected to be tested for their effect on the cell cycle of the aforementioned cell line. The results revealed a significant elevation in the percent of Anx V-FITC by 29.7% and 25.17%, respectively (Figure 4), which parallels an increase for the entire apoptosis percentages by 33.75- and 28.60-fold, correspondingly, in comparison to the control (DMSO). This clearly verifies that cell death resulting from the anti-proliferative action of the target hybrids was attributable to physiological apoptosis and not to nonspecific necrosis.

#### 2.2.5. CDK Inhibitory Activity

##### CDK4 Enzyme Assay

The most efficient anti-proliferative agents reported here towards MCF-7 and/or MDA-MB-231 cancer cell lines within both *N*-unsubstituted **6** and *N*-substituted **9** series (**6a–h**, **9a**, and **9e**) were initially evaluated for their percent inhibition of CDK4 at a dose of 20 μM (Table 5). The inhibitory results obtained for CDK4 were variable for the tested compounds. Where compounds **6a** (92%) and **6e** (93%) showed stunning inhibitory activities, the rest of the examined hybrids showed low to moderate activity with percent inhibition spanning in the range of 12% to 46% (Table 5). 

Accordingly, compounds **6a** and **6e** were further evaluated for their IC_50_ values against CDK4. As presented in Table 5, both of the tested conjugates displayed good inhibitory activity toward CDK4 with IC_50_ values equal 1.82 and 1.26 µM, correspondingly.

##### Screening of CDK2 and CDK9 Inhibitory Activities

With the aim of screening and profiling the inhibitory activity for the target compounds toward other CDK isoforms, compounds **6a–h**, **9a**, and **9e** were screened for their potential inhibitory activities against CDK2 and CDK9 isoforms. The ten compounds were tested for their single dose percent inhibition (20 μM) (Table 6). 

The presented results in Table 6 revealed that CDK9 was weakly inhibited by all the examined compounds with percent inhibition range of 4%–32%, which proves that these compounds do not have significant inhibitory activity against this isoform. 

On the other hand, inhibition of CDK2 ranged from good to weak, with percent inhibition range of 45%–85% (Table 6). In particular, **6a** and **6d** were the best developed CDK2 inhibitors here with percent inhibition of 78% and 85%, respectively. In addition, compounds **6c**, **6e**, **6f**, and **6h** displayed percent inhibition equal to or higher than 50% toward this isoform at a concentration of 20 μM.

In conclusion, the results of inhibition of the three tested CDK isoforms (CDK2, 4, and 9) disclosed the inability of the target compounds to inhibit CDK9 significantly, with concomitant good inhibitory activities against CDK2 and CDK4. Accordingly, further optimization for the here reported oxindoles considering their plausible dual CDK2 and CDK4 inhibitory actions are currently in development and expected to be conveyed in the future.

### 2.3. CDK4 Molecular Docking Study and Structure–Activity Relationship

A protein data bank (PDB) search showed that there are not any CDK4 protein structures co-crystalized with an inhibitor available (holoprotein). The available structures are apoprotein structures for CDK4 in complex with cyclin D such as PDB ID 2W96, 2W9Z, 2W9F, and 2W99 [43]. On the other hand, there are more than two hundred crystal structures obtainable in the PDB for CDK2 that are co-crystalized with an inhibitor [44]. The overall homology between CDK2 and CDK4 is 45%, suggesting that both proteins are folded in a similar fashion [45]. To define CDK4 kinase inhibitors’ binding site and to induce its bound state shape and topology, the CDK4 protein structure (PDB ID: 2W96) [43] was aligned to the CDK2 protein structure (PDB ID: 4FX3) co-crystalized with an oxindole inhibitor. CDK2 protein was then deleted leaving behind the oxindole ligand in the CDK4 binding site followed by energy minimization of the CDK4 protein structure. The final resulting structure contained CDK4 protein bound to an oxindole inhibitor interacting with the key amino acids Glu94 and Val96 in the CDK4 binding site.

**Figure 5:**
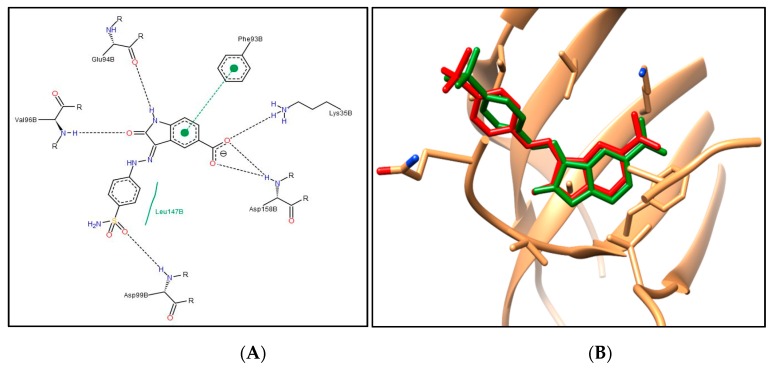
(**A**) The 2D diagram for the docking pose of the oxindole ligand in CDK4 binding site; (**B**) 3D illustration of the superimposition for the docking pose (green) and the added (red) oxindole ligand in the CDK4 binding site (RMSD = 0.879 Å).

First, the molecular docking protocol was validated by performing self-docking for the added oxindole ligand in the vicinity of the CDK4 binding site. The self-docking validation replicated the binding pattern for the added ligand, efficiently demonstrating the aptness of the used setup for the planned docking study, and this was confirmed by the low RMSD of 0.879 Å between the docked pose and the added ligand (energy score (S) = −9.72 kcal/mol) (Figure 5).

**Figure 6:**
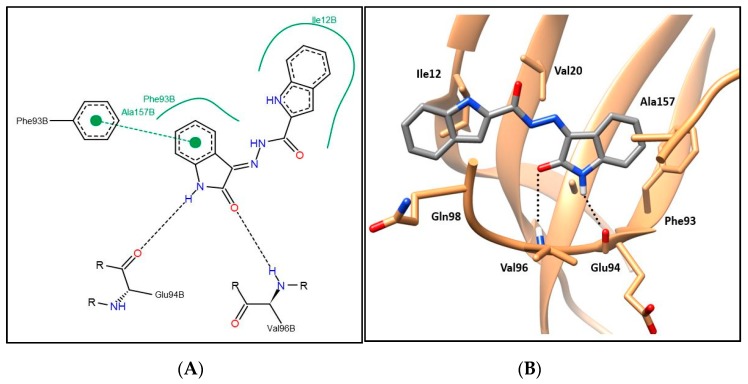
(**A**) The 2D diagram; (**B**) 3D representation for hybrid **6a** displaying its interaction with the CDK4 binding site.

**Figure 7:**
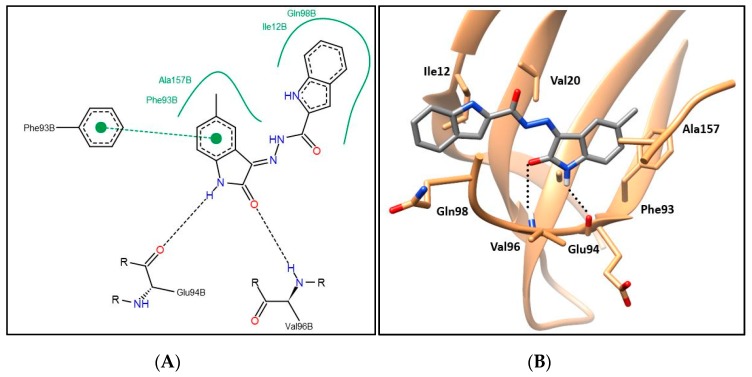
(**A**) The 2D diagram; (**B**) 3D representation for hybrid **6e** displaying its interaction with the CDK4 binding site.

Compounds **6a–i** showing promising cytotoxic as well as CDK4 inhibitory activity were docked in the CDK4 binding site in order to justify their inhibitory action and investigate their binding patterns. In general, hybrids **6a–i** achieved similar binding patterns, where the oxindole framework was fitted in the ATP adenine binding pocket like that of the added ligand in the hinge region. As observed in Figure 6 and Figure 7, the oxindole ring was engaged in hydrogen bond interactions via both (NH) and (C=O) functional groups with the key Glu94 and Val96 amino acids, respectively, which indicates the importance of the unsubstituted NH to be available for interaction with Glu94, which rationalizes the lower activity of series **9** with *N*-substituted derivatives. Moreover, the oxindole ring interacts by its phenyl part through π–π stacking interaction with the side chain phenyl of Phe93 and through hydrophobic interaction with the side chains of Phe93 and Ala157. A hydrophobic substituent with the appropriate size on the oxindole ring 5-position could improve the binding affinity, which rationalizes the promising activity of **6e** with a 5-methyl substitution. Thus, derivatives in this series carrying substituents with hydrophilic characters, **6b**, **6f**, and **6g**, or bulky size, **6c**, **6d**, **6h**, and **6i**, showed lower CDK4 inhibitory activity as reflected in their CDK4 inhibitory actions and docking scores (Table 5 and Table 7).

The indole part of the target hybrids was fitted in a hydrophobic sub-pocket of CDK4 active site and engaged in a hydrophobic interaction with the hydrophobic pocket depicted by the amino acids Ile12, Val20, and Gln98 lining this sub-pocket (Figure 6 and Figure 7, and for further details see Supplementary Materials).

## 3. Materials and Methods

### 3.1. Chemistry

#### 3.1.1. General

Infrared (IR) spectra were recorded as KBr disks by the use of a Schimadzu FT-IR 8400S spectrophotometer. NMR spectra were recorded on JEOL ECA-500 II (500/125 MHz) or Bruker (400/100 MHz) NMR spectrophotometers using deuterated dimethylsulfoxide (DMSO-*d_6_*). All coupling constant (*J*) values were expressed in hertz. The used abbreviations are as following: s, singlet; d, doublet; m, multiplet. Compound **3** [41], **8a–f** [46,47], **4** [48], and **6a–d** [49] were synthesized in accordance with the literature procedures.

#### 3.1.2. Preparation of Key Intermediate 1H-Indole-2-Carbohydrazide 4

An excess of 99% hydrazine monohydrate (3.7 mL, 75 mmol) was added to a hot solution of ethyl indole-2-carboxylate **3** (2.84 g, 15 mmol) in ethyl alcohol (25 mL). The resulting reaction mixture was heated under reflux for two hours. Thereafter, it was cooled to r.t. and then poured over crushed ice. The formed solid was filtered-off, washed with water (3 × 5 mL), and recrystallized from isopropyl alcohol to produce the key intermediate 1*H*-indole-2-carbohydrazide **4**. 

#### 3.1.3. Synthesis of Final Compounds **6a–i, 9a–f, and 11a,b**

A solution of indole-2-carbohydrazide **4** (210 mg, 1.2 mmol) in glacial acetic acid (15 mL) was treated with the appropriate isatin derivative **5a–i** and **8a–f** (1.2 mmol), or ketones **10a** and **10b** (1.2 mmol). The resulting reaction mixture was refluxed for four hours then cooled to r.t. The obtained precipitate was collected by filtration and dried to get a powder that was recrystallized from glacial acetic acid to furnish the titled conjugates **6a–i, 9a–f**, and **11a,b**.

Details for the full characterization of the key intermediate (**4**) and the target hybrids (**6a–i, 9a–f**, and **11a,b**) are provided in the Supplementary Materials.

### 3.2. Biological Evaluation 

Details for experimental protocols used in the diverse biological assays for the targeted hybrids (**6a–i, 9a–f**, and **11a,b**) are provided in the Supplementary Materials.

#### 3.2.1. Anti-Proliferative Activities Against Human Breast Cancer Cell Lines

Target compounds **6a–i, 9a–f**, and **11a,b** were tested for their potential anti-proliferative potency toward two breast cancer cell lines (MCF-7 and MDA-MB-231) that obtained from American Type Culture Collection. Cytotoxicity was assessed following the SRB colorimetric assay protocol [42], as reported earlier [50,51].

#### 3.2.2. Cell Cycle Analysis

Flow cytometric analysis (FACS) was performed to assess the cell cycle distributions in MCF-7 breast cancer cells after treatment by hybrids **6e** and **6f** at their IC_50_ (0.39 and 1.85 µM, respectively), by BD FACS Calibur flow cytometer, as described previously [36].

#### 3.2.3. Apoptosis study 

Assessment of the levels for the proapoptotic markers (Bax, caspase-3, and p53) as well as the antiapoptotic marker (Bcl-2) was carried out through the use of ELISA colorimetric kits in accordance with the manufacturer’s instructions, as mentioned earlier [36]. In addition, the pro-apoptotic potential of indoles **6e** and **6f** towards MCF-7 cells was evaluated through an FITC Annexin V apoptosis detection kit by flow cytometry, in accordance with the manufacturer’s protocol and referring to the reported procedures [52].

#### 3.2.4. CDK Kinase Inhibitory Activity

The in vitro CDK kinase inhibition assay was performed by Reaction Biology Corp. (PA, USA) (http://www.reactionbiology.com) Kinase HotSpotSM service.

#### 3.2.5. Molecular Modeling Study

##### Defining CDK4 Binding Site and Adjusting Its Shape and Topology

The CDK2 protein structure (PDB ID: 4FX3) co-crystalized with an oxindole inhibitor and the CDK4 protein structure (PDB ID: 2W96) [43] were downloaded from the protein data bank. Protein structures were aligned using their backbone α-carbons using the *MatchMaker* command available in UCSF Chimera package 1.12 [53]. Then, the CDK2 protein was deleted, leaving behind the oxindole ligand in CDK4 binding site. MOE 2010.10 software was used for energy minimization of the CDK4 protein structure containing the added oxindole ligand. These procedures yielded a final structure containing CDK4 protein bound to an oxindole inhibitor interacting with the key amino acids in the CDK4 binding site, Glu94 and Val96 (Figure 1). 

##### Molecular Docking

For carrying out the molecular docking for the target hybrids in the CDK4 binding site, the following procedures were adopted. 

(A) Ligand preparation: The synthesized hybrids as well as the added oxindole ligand were built as 3D structures using Discovery Studio Visualizer 2017R2 [54]. The OMEGA 3.0.0.1 program in OpenEye package was used to generate optimal conformers to be used for docking pose prediction using *Pose* mode [55,56].

(B) Protein preparation: The generated structure for CDK4 in step 3.2.5.1. with the added oxindole inhibitor was used in this step. Using Discovery Studio Visualizer 2017R2 [54], the protein was prepared for the docking study. The cyclin D chain, water molecules, and ligands that were not involved in the binding were removed. The GUI module “MakeReceptor 3.2.0.2” from the “OEDocking 3.2.0.2” program in OpenEye package was used for further protein preparation and to define the active site and the docking box for molecular docking [57,58,59,60].

(C) Molecular docking: The HYBRID docking module of “OEDocking 3.2.0.2” program in OpenEye package was utilized to carry out the molecular docking for the generated conformers of the herein reported hybrids as well as the oxindole inhibitor in the CDK4 binding site using the *Chemgauss4* scoring function [57,58,59,60].

First, the molecular docking protocol was validated by performing self-docking for the added oxindole ligand in the CDK4 binding site, producing a docking pose with energy score (S) and RMSD equal to −9.72 kcal/mol and 0.879Å, respectively. Then, this validated docking setup was adopted to investigate the ligand–target interactions in the CDK4 active site for the prepared hybrids to explore their binding pattern and to justify their binding affinity. PoseView 1.1.2 was used to generate the 2D figures of the ligand–target interactions [61,62,63,64], whereas UCSF Chimera package 1.12 was used to generate the 3D molecular graphics of the ligand–target interactions [53]. 

## 4. Conclusions

The current study presents the development of two series of oxindole–indole hybrids (**6a–i** and **9a–f**) and carbocycle–indole hybrids (**11a**,**b)** as efficient antitumor agents with potential inhibitory action toward CDK4. All the prepared hybrids (**6a–i, 9a–f**, and **11a,b**) were examined for the potential cytotoxic activity towards MCF-7 and TNBC MDA-MB-231 breast cancer cell lines via SRB assay. The synthesized oxindole–indole conjugates, except **6i**, **9b** and **9c**, efficiently affected the growth of the human breast cancer MCF-7 (IC_50_: 0.39 ± 0.05–21.40 ± 1.58) and/or MDA-MB-231 (IC_50_: 1.03 ± 0.04–22.54 ± 1.67) cell lines, whereas bioisosteric replacement of the oxindole motif with indane or tetralin rings (conjugates **11a**,**b**) diminished the anti-proliferative activity, indicating the importance of the oxindole scaffold for the antitumor activity. Moreover, hybrids **6e** and **6f** triggered cell cycle arrest and apoptosis in MCF-7 cancer cells as explicated by their capabilities to considerably boost the Bax/Bcl-2 ratio, and to up-regulate the level of caspase-3 and p53. Furthermore, single dose (20 μM) inhibitory activity of the most efficient anti-proliferative agents (**6a–h**, **9a** and **9e**) was initially evaluated toward CDK4. Hybrids **6a** and **6e** showed the most potent inhibitory activity of 92% and 93%, respectively, whereas the rest of the examined hybrids showed low to moderate activity with percent inhibition of 12%–46%. Thereafter, the IC_50_ values for hybrids **6a** and **6e** in CDK4 were further determined and were found to be 1.82 and 1.26 µM, respectively. The molecular docking study revealed that the oxindole moiety was engaged in two hydrogen bonding interactions via both (NH) and (C=O) functionalities with the key amino acids Glu94 and Val96, respectively, rationalizing the lower activity of series **9** with *N*-substituted derivatives. The oxindole ring interacted by its phenyl part through π–π stacking interactions with the side chain phenyl of Phe93 and through hydrophobic interaction with the hydrophobic side chains of Phe93 and Ala157. In addition, the indole framework was stably accommodated in a hydrophobic sub-pocket, establishing a hydrophobic interaction with the hydrophobic side chains of amino acids Ile12, Val20, and Gln98 lining this sub-pocket. Finally, compounds **6a–h**, **9a**, and **9e** were screened for their potential inhibitory activities against CDK2 and CDK9 isoforms. While CDK9 was weakly inhibited with percent inhibition range of 4%–32%, inhibition of CDK2 ranged from good to weak, with percent inhibition range of 45%–85%. Collectively, these results highlighted hybrids **6a** and **6e** as good leads for further optimization as promising antitumor drugs toward breast malignancy and CDK inhibitors.

## Figures and Tables

**Table 1:**
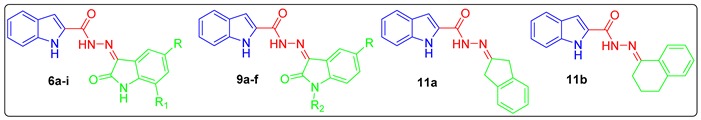
Anti-proliferative activities for hybrids **6a–i**, **9a–f**, and **11a**,**b** against MCF-7 and MDA-MB-231 breast cancer cell lines.

Comp.	R	R_1_	R_2_	IC_50_ (µM)^a^	
				MCF-7	MDA-MB-231
**6a**	H	H	-	3.12 ± 0.14	15.10 ± 0.73
**6b**	F	H	-	6.0 ± 0.32	6.36 ± 0.29
**6c**	Cl	H	-	2.72 ± 0.17	9.48 ± 0.44
**6d**	Br	H	-	3.31 ± 0.11	18.33 ± 0.71
**6e**	CH_3_	H	-	0.39 ± 0.05	22.54 ± 1.67
**6f**	OCH_3_	H	-	1.85 ± 0.08	2.85 ± 0.9
**6g**	OCF_3_	H	-	5.07 ± 0.21	7.19 ± 0.38
**6h**	NO_2_	H	-	21.40 ± 1.58	1.85 ± 0.07
**6i**	CH_3_	CH_3_	-	47.16 ± 3.02	NA^b^
**9a**	H	-	-CH_3_	5.50 ± 0.28	8.39 ± 0.27
**9b**	H	-	-CH_2_CH_2_CH_3_	40.89 ± 3.17	18.50 ± 0.85
**9c**	H	-	-CH_2_COOC_2_H_5_	67.51 ± 3.02	NA^b^
**9d**	H	-	-CH_2_C_6_H_5_	9.56 ± 0.61	6.50 ± 0.39
**9e**	H	-	-CH_2_C_6_H_4_-4-F	3.70 ± 0.19	1.03 ± 0.04
**9f**	Cl	-	-CH_2_CH_2_CH_3_	19.26 ± 0.65	9.44 ± 0.35
**11a**	-	-	-	84.70 ± 4.02	NA^b^
**11b**	-	-	-	NA^b^	NA^b^
**Staurosporine**	-	-	-	6.81 ± 0.22	10.29 ± 0.72

**^a^** IC_50_ values reported as the mean ± S.D. from 3 separate experiments, ^b^ NA: Hybrids possessing IC_50_ more than 100 µM.

**Table 2:** Effect of compounds **6e** and **6f** on the cell cycle phases of MCF-7 cells.

Comp.	%G0–G1	%S	%G2/M	%Sub-G1
**6e**	19.23	14.81	41.47	24.49
**6f**	22.19	19.29	36.26	22.26
**Control**	56.45	27.45	14.73	1.37

**Table 3:** Effect of conjugates **6e** and **6f** on the expression levels of Bcl-2 and Bax in MCF-7 cancer cells.

Cpd.	Bax (pg/mg of Total Protein)	Bcl-2 (ng/mg of Total Protein)	Bax/Bcl-2
**6e**	325.3 ± 12.1	2.19 ± 0.10	148.5
**6f**	268.7 ± 9.5	2.73 ± 0.13	98.6
**Control**	38.3 ± 2.2	4.65 ± 0.23	8.2

**Table 4:** Effect of compounds **6e** and **6f** on the expression levels of active caspase-3 and p53 in MCF-7 cancer cells.

Cpd.	Caspase-3 (pg/mg)	p53 (pg/mg)
**6e**	411.70 ± 12.3	704.39 ± 32.8
**6f**	364.15 ± 14.8	547.60 ± 29.5
**Control**	35.92 ± 1.8	41.26 ± 2.7

**Table 5:** Inhibitory effect of compounds **6a–h**, **9a**, and **9e** on CDK4 at a single-dose of 20 µM, and IC_50_ values of the most potent compounds **6a** and **6e.**

Comp.	CDK4	
	% Inhibition	IC_50_ (µM)
**6a**	**92**	**1.82**
**6b**	33	-
**6c**	28	-
**6d**	43	-
**6e**	**93**	**1.26**
**6f**	14	-
**6g**	19	-
**6h**	46	-
**9a**	20	-
**9e**	12	-
**Staurosporine**	**99**	**0.017**

**Table 6:** Inhibitory effect of compounds **6a–h**, **9a**, and **9e** on CDK2 and CDK9 at a single-dose of 20 µM.

Comp.	% Enzyme Inhibitory Activity	
	CDK2	CDK9
**6a**	78	20
**6b**	48	7
**6c**	57	11
**6d**	85	32
**6e**	68	18
**6f**	50	8
**6g**	14	4
**6h**	67	10
**9a**	41	13
**9e**	5	9
**Staurosporine**	99	98

**Table 7:** Docking energy scores (S) in kcal/mol for the conjugates **6a–i** and the added oxindole ligand in the CDK4 binding site.

Comp.	Energy Score (S) kcal/mol
**6a**	−8.60
**6b**	−8.43
**6c**	−8.27
**6d**	−7.83
**6e**	−8.36
**6f**	−7.62
**6g**	−6.61
**6h**	−7.65
**6i**	−8.04
**Oxindole ligand**	−9.72

## Supplementary Materials

The following are available online at https://www.mdpi.com/1420-3049/25/9/2031/s1, Figure S1-S7: 2D diagram for target hybrids **6b-d** and **6f-i** showing their interactions with the CDK4 binding site; Experimental procedures for the biological assays; Characterization of intermediate (**4**) and the target hybrids (**6a–i, 9a–f** and **11a, b**); NMR Spectra and IR Spectra.
