# Supplementary material for: Synthesis, Biological Evaluation and In Silico Studies of Certain Oxindole–Indole Conjugates as Anticancer CDK Inhibitors
Source: Molecules. 2020 Apr 27;25(9):2031. doi: 10.3390/molecules25092031 (PMC7248897; doi:10.3390/molecules25092031)
Supplement: Supplementary file 1 [file molecules-25-02031-s001.pdf]

Supporting Information

Synthesis, biological evaluation and *in silico* studies of certain oxindole-indole conjugates as anticancer CDK inhibitors

Tarfah Al-Warhi, Ahmed M. El Kerdawy, Nada Aljaeed, Omnia E. Ismael, Rezk R. Ayyad,Wagdy M. Eldehna*, Hatem A. Abdel-Aziz, Ghada H. Al-Ansary*

Tables of Contents

**1.**	Anti-proliferative Activity Against Breast Cancer Cell Lines	3
**2.**	Cell Cycle Analysis	3
**3.**	ELISA Immunoassay	4
**4.**	AnnexinV-FITC/PI Apoptosis Assay	4
**5.**	CDK Kinase Inhibitory Activity	5
**6.**	2D Diagrams for hybrids **6b-d** and **6f-i** showing their interactions with the CDK4 binding site	6-9
**7.**	Characterisation of intermediate (**4**) and the target hybrids (**6a-i, 9a-f and 11a, b**)	10-13
**8.**	NMR Spectra	14-35
**9.**	IR Spectra	36-49

1. Anti-proliferative action toward human breast cell lines

The two examined human breast cancer cell lines (MCF-7 and Breast MDA-MB-231) have been obtained from American Type Culture Collection (ATCC). The cells were maintained in Dulbecco's modified Eagle's medium (DMEM) supplemented with 10% heat inactivated fetal calf serum (GIBCO), penicillin (100 U/ml) and streptomycin (100 μg/ml) at 37 °C in humidified atmosphere containing 5% CO_2. Cells at a concentration of 0.50 x 10^6 were grown in a 25 cm^2 flask in 5 ml of culture medium.

The anti-proliferative activity of the tested hybrids **6a-i**, **9a-f** and **11a**, **b** was measured *in vitro* using the Sulfo-Rhodamine-B stain (SRB) assay. Briefly, Cells were inoculated in 96-well microtiter plate (5X10^4 cells/ well) for 24 h before treatment with the tested compounds to allow attachment of cell to the wall of the plate. Tested compounds were dissolved in DMSO at 1 mg/ml immediately before use and diluted to the appropriate volume just before addition to the cell culture. Different concentrations of tested compounds, doxorubicin and sorafenib were added to the cells (three wells were prepared for each individual dose). Cells were incubated with the compounds for 48 h at 37°C and in atmosphere of 5% CO_2. After 48 h cells were fixed, washed, and stained for 30 min with 0.4% (w/v) SRB dissolved in 1% acetic acid. Unbound dye was removed by four washes with 1% acetic acid, and attached stain was recovered with Tris-EDTA buffer. Color intensity was measured in an ELISA reader. The relation between percent of surviving fraction and drug concentration is plotted to get the survival curve for each cell line. The concentration required for 50% inhibition of cell viability (IC_{50}) was calculated.

2. Cell Cycle Analysis

Breast cancer MCF-7 cells were treated with hybrids **6e and 6f** for 24 h (at their IC_{50} concentration), and then cells were washed twice with ice-cold phosphate buffered saline (PBS). Subsequently, the treated cells were collected by centrifugation, fixed in ice-cold 70% (*v/v*) ethanol, washed with PBS, re-suspended with 100 μg/mL RNase, stained with 40 μg/mL PI, and analyzed by flow cytometry using FACS Calibur (Becton Dickinson, BD, Franklin Lakes, NJ, USA). The cell cycle distributions were calculated using CellQuest software 5.1 (Becton Dickinson).

3. ELISA Immunoassay

The levels of the pro-apoptotic markers (Bax, caspase-3 and p53) as well as the anti-apoptotic marker Bcl-2 were determined using ELISA colorimetric kits per the manufacturer's instructions. Breast cancer MCF-7 cells were cultured as a monolayer in T-25 flasks and were seeded to attain 30% confluency prior to treatment. Cells were then treated separately with hybrids **6e and 6f** at their IC_{50} concentrations for 48 h. At the end of treatment, cells were collected via trypsinization and centrifuged at 10,000 rpm. The pellet was then rinsed with PBS and lysed in RIPA lysis buffer at 4 °C for 45 min, then centrifuged at 14,000 rpm for 20 min to remove the cellular debris. Lysates were then collected and stored at −80 °C for later protein determination using Pierce BCA Protein Assay Kit according to manufacturer's recommendations.

The cell lysate was diluted 10 times, and 100 μL (50 mg protein) was added to the wells of four separate microtiter plates for the four ELISA kits that were pre-coated with primary antibodies specific to Bax, Bcl-2, caspase-3 and p53 proteins, respectively. A secondary biotin-linked antibody specific to the protein captured by the primary antibody was further added to bind the captured protein, forming a "sandwich" of specific antibodies around the desired protein in the cell lysate. The streptavidin-HRP complex was then used to bind the biotin-linked secondary antibody through its streptavidin portion. The HRP domain reacted with the added TMB substrate to form a colored product that measured at 450 nm by a plate reader ChroMate-4300 after the reaction was terminated *via* the addition of stop solution.

4. Annexin V-FITC Apoptosis Assay

Phosphatidylserine externalization was assayed using Annexin V-FITC/PI apoptosis detection kit (BD Biosciences, USA) according to the manufacturer's instructions. Breast cancer MCF-7 cells were cultured to a monolayer then treated with hybrids **6e and 6f** at their IC_{50} concentration. Briefly, cells were then harvested *via* trypsinization, and rinsed twice in PBS followed by binding buffer. Moreover, cells were re-suspended in 100 μL of binding buffer with the addition of 1 μL of FITC-Annexin V followed by an incubation period of 30 min at 4 °C. Cells were then rinsed in binding buffer and resuspended in 150 μL of binding buffer with the addition of 1 μL of DAPI (1 μg/μL in PBS). Cells were then analyzed using the flow cytometer BD FACS Canto II and the results were interpreted with FlowJo7.6.4 software (Tree Star, Ashland, OR, USA).

5. CDK Kinase Inhibitory Activity

Reaction Biology Corp. Kinase HotSpotSM service (http://www.reactionbiology.com) was used for screening of tested compounds. CDK Kinase inhibitory activities were assessed by the HotSpot assay platform, which contained specific kinase/substrate pairs along with required cofactors. Base reaction buffer: 20 mM Hepes (pH 7.5), 10 mM $MgCl_2$, 1 mM EGTA, 0.02% Brij35, 0.02 mg/ml BSA, 0.1 mM Na_3VO_4, 2 mM DTT, 1% DMSO. Testing compounds were dissolved in 100% DMSO to specific concentration. The serial dilution was conducted by Integra Viaflo Assist in DMSO. The reaction mixture containing the examined compound and 33P-ATP was incubated at room temperature for 2 h and radioactivity was detected by filter-binding method. Kinase activity data were expressed as the percent remaining kinase activity in test samples compared to vehicle (dimethyl sulfoxide) reactions.

6. 2D Diagrams for hybrids 6b-d and 6f-i showing their interactions with the CDK4 binding site

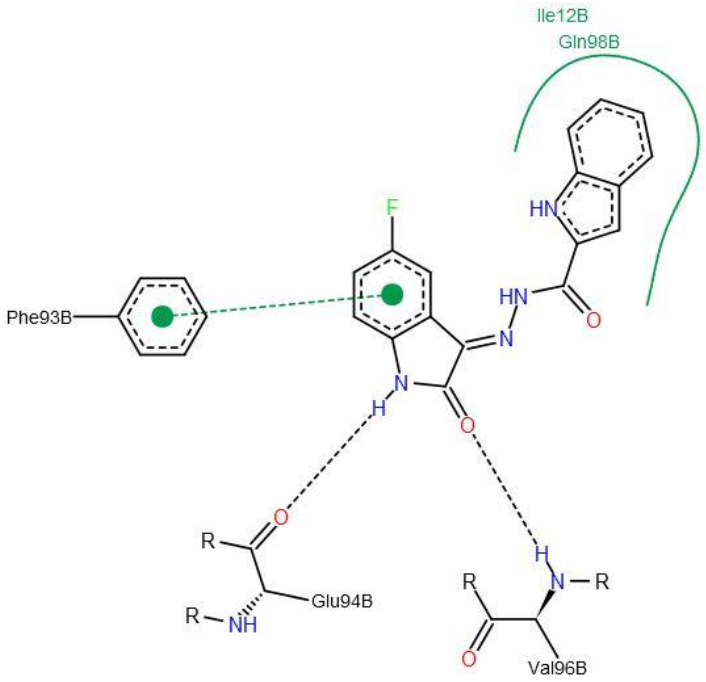

Figure S1. 2D diagram for hybrid **6b** showing its interaction with the CDK4 binding site.

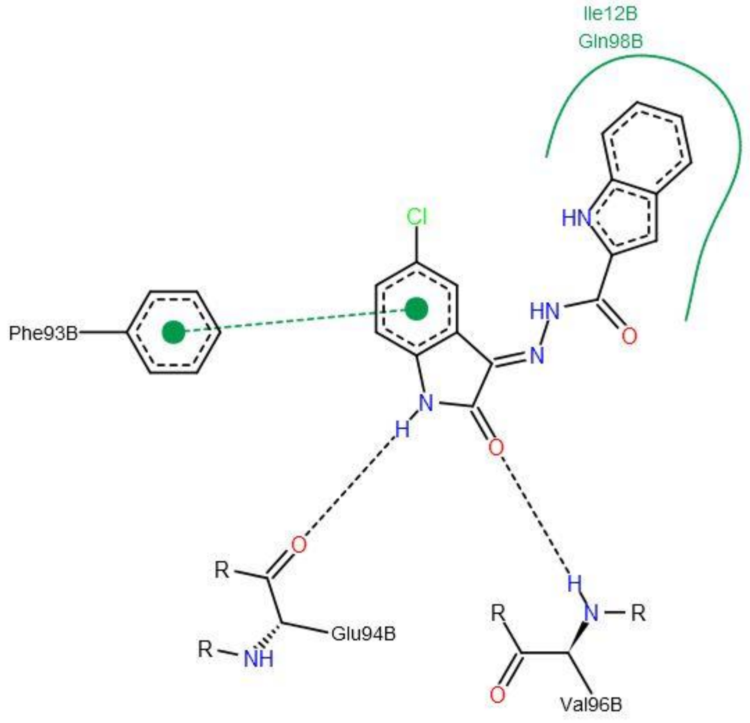

Figure S2. 2D diagram for hybrid **6c** showing its interaction with the CDK4 binding site.

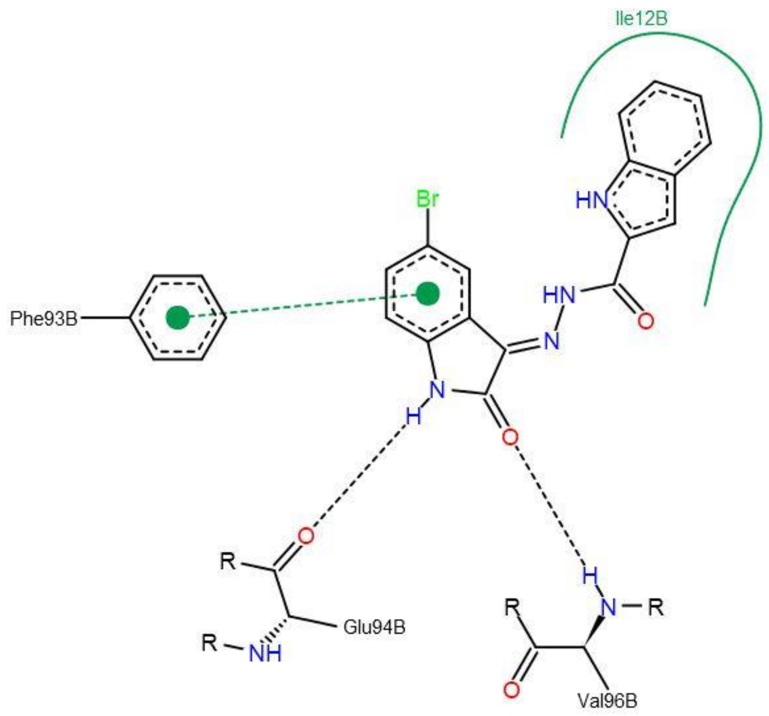

Figure S3. 2D diagram for hybrid **6d** showing its interaction with the CDK4 binding site.

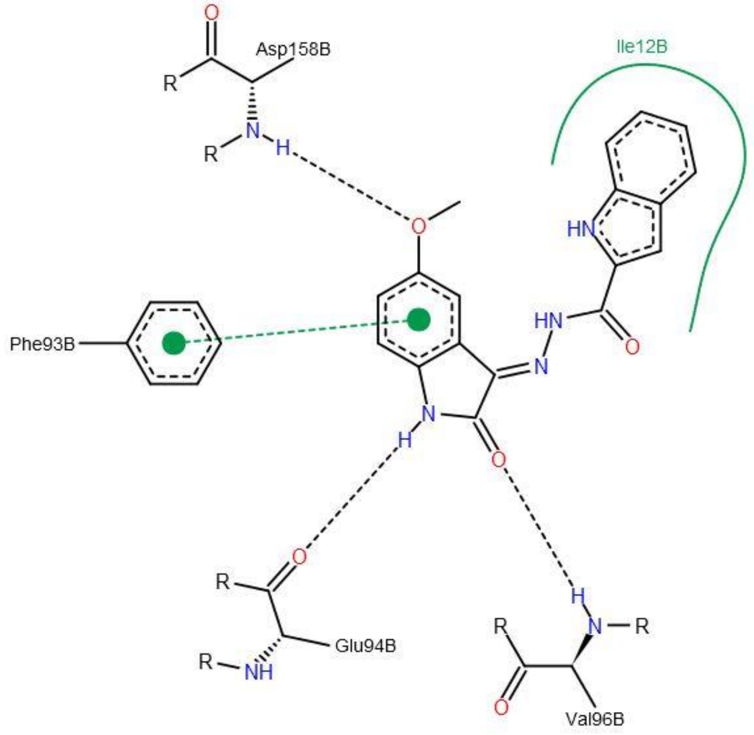

Figure S4. 2D diagram for hybrid **6f** showing its interaction with the CDK4 binding site.

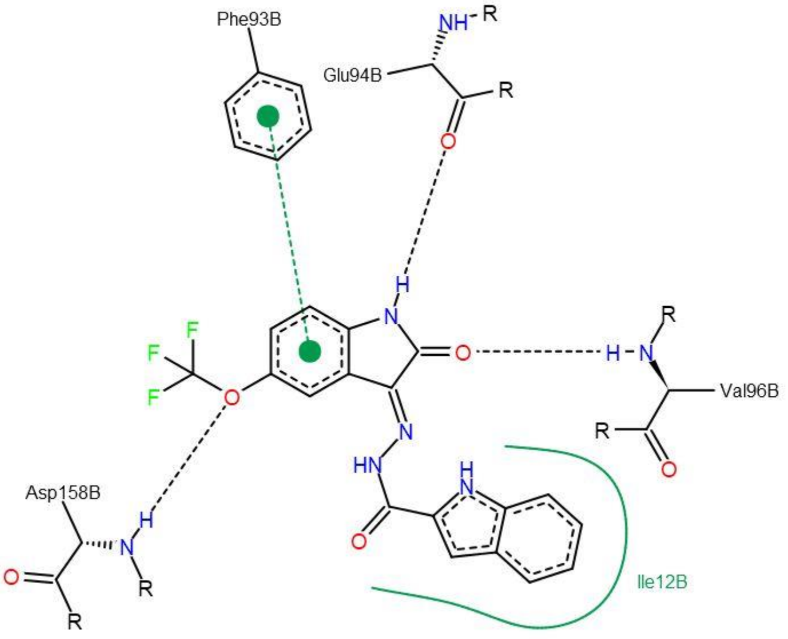

Figure S5. 2D diagram for hybrid **6g** showing its interaction with the CDK4 binding site.

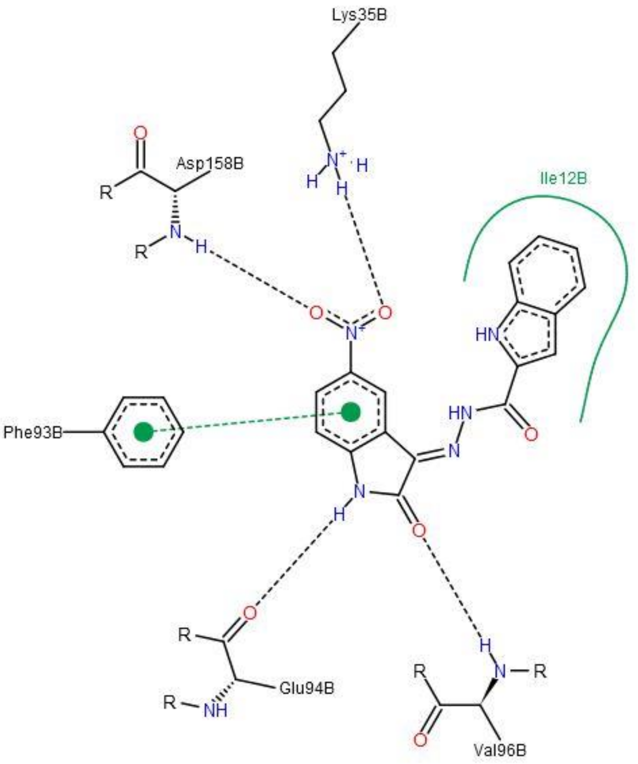

Figure S6. 2D diagram for hybrid **6h** showing its interaction with the CDK4 binding site.

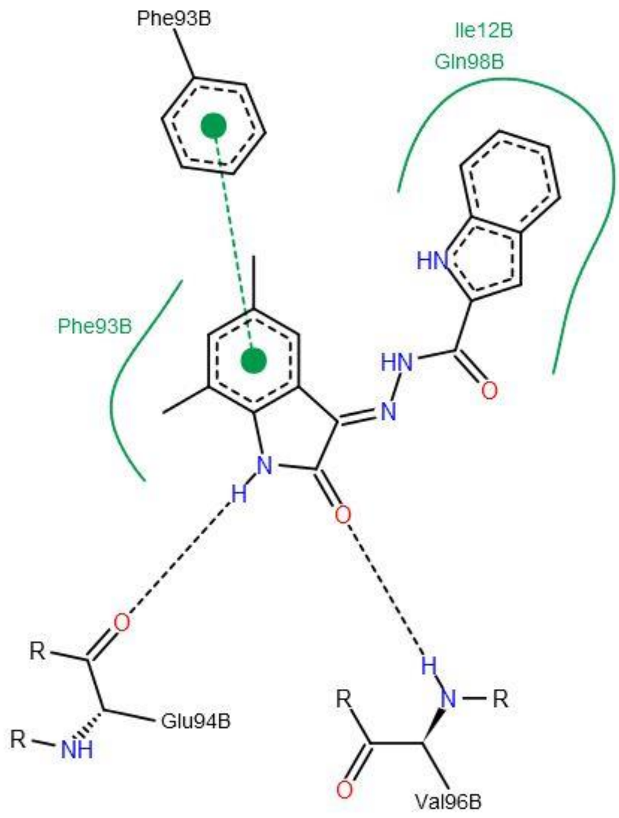

Figure S7. 2D diagram for hybrid **6i** showing its interaction with the CDK4 binding site.

7. Characterisation of intermediate (4) and the target hybrids (6a-i, 9a-f and 11a, b)

1H-indole-2-carbohydrazide 4

Yield 84 %, m.p. 249-251 °C (reported: 247-248 °C [48]); ^{1}H NMR (500 MHz) *δ ppm*: 4.51 (s, 2H, NH_2), 6.99 (t, 1H, *J* = 8.5 Hz, Ar_H), 7.07 (d, 1H, *J* = 2.0 Hz, Ar_H), 7.13 (dt, 1H, *J* = 1.5 and 8.5 Hz, Ar_H), 7.41 (d, 1H, *J* = 7.5 Hz, Ar_H), 7.56 (d, 1H, *J* = 8.0 Hz, Ar_H), 9.78 (s, 1H, NH), 11.60 (s, 1H, NH); ^{13}C NMR (125 MHz) *δ ppm*: 101.85, 112.28, 119.73, 121.43, 123.14, 127.13, 130.51, 136.33, 161.27

N'-(2-Oxoindolin-3-ylidene)-1H-indole-2-carbohydrazide 6a.

Yield 81 %, m.p. > 300 °C (reported: > 300 °C [49]); Purity 97%; IR: 3362, 3168, 3070 (3NH), 1727, 1658 (2C=O); ^{1}H NMR (500 MHz) *δ ppm*: 6.91 (d, 1H, *J* = 7.5 Hz, Ar_H), 7.07-7.11 (m, 2H, Ar_H), 7.24 (t, 1H, *J* = 8.0 Hz, Ar_H), 7.39 (t, 1H, *J* = 7.0 Hz, Ar_H), 7.47 (d, 1H, *J* = 8.5 Hz, Ar_H), 7.57 (s, 1H, Ar_H), 7.70 (d, 1H, *J* = 8.0 Hz, Ar_H), 8.01 (d, 1H, *J* = 7.5 Hz, Ar_H), 10.87 (s, 1H, NH), 11.68 (s, 1H, NH), 11.95 (s, 1H, NH); ^{13}C NMR (125 MHz) *δ ppm*: 110.68, 111.25, 112.52, 112.61, 115.62, 119.87, 120.25, 120.49, 120.96, 121.89, 122.24, 122.77, 124.65, 124.87, 126.73, 126.95, 127.04, 128.77, 131.69, 132.78, 137.13, 137.47, 142.38, 143.93, 160.73, 161.01, 163.16, 164.79; MS *m/z* [%]: 304.2 [M^+, 46.86], 144.02 [100]; Anal. calcd. For $C_{17}H_{12}N_4O_2$: C, 67.10; H, 3.97; N, 18.41; Found C, 66.83; H, 4.01; N, 18.53.

N'-(5-Fluoro-2-oxoindolin-3-ylidene)-1H-indole-2-carbohydrazide 6b.

Yield 82 %, m.p. > 300 °C (reported: > 300 °C [49]); Purity 96%; IR: 3453, 3372, 3189 (3NH), 1723, 1649 (2C=O); ^{1}H NMR (500 MHz) *δ ppm*: 6.89-6.92 (m, 1H, Ar_H), 7.07 (t, 1H, *J* = 8.0 Hz, Ar_H), 7.23-7.29 (m, 2H, Ar_H), 7.47 (d, 1H, *J* = 7.5 Hz, Ar_H), 7.64 (s, 1H, Ar_H), 7.71 (d, 1H, *J* = 7.5 Hz, Ar_H), 7.98 (d, 1H, *J* = 8.5 Hz, Ar_H), 10.88 (s, 1H, NH), 11.79 (s, 1H, NH), 11.95 (s, 1H,); ^{13}C NMR (125 MHz) *δ ppm*: 111.38, 111.44, 112.30, 112.52, 112.63, 113.71, 113.92, 115.79, 115.87, 117.89, 118.08, 118.82, 119.01, 120.25, 120.52, 121.11, 122.35, 124.78, 124.98 126.96, 127.05, 128.52, 137.12, 137.54, 138.61, 140.25, 156.50, 157.49, 158.38, 159.39, 161.26, 163.29, 164.94; MS *m/z* [%]: 322.02 [M^+, 21.90], 144.05 [46.89], 89.19 [100]; Anal. calcd. For $C_{17}H_{11}FN_4O_2$: C, 63.35; H, 3.44; N, 17.38; Found C, 63.49; H, 3.42; N, 17.45.

N'-(5-Chloro-2-oxoindolin-3-ylidene)-1H-indole-2-carbohydrazide 6c.

Yield 85 %, m.p. > 300 °C (reported: > 300 °C [49]); Purity 98%; IR: 3442, 3347, 3137 (3NH), 1721, 1657 (2C=O); ^{1}H NMR (500 MHz) *δ ppm*: 6.91 (d, 1H, *J* = 8.0 Hz, Ar_H), 7.07 (t, 1H, *J* = 8.0 Hz, Ar_H), 7.25 (t, 1H, *J* = 7.5 Hz, Ar_H), 7.42-7.45 (m, 1H, Ar_H), 7.47 (d, 1H, *J* = 8.5 Hz, Ar_H), 7.63 (s, 1H, Ar_H), 7.71 (d, 1H, *J* = 8.0 Hz, Ar_H), 8.19 (s, 1H, Ar_H), 10.99 (s, 1H, NH), 11.85 (s, 1H, NH), 11.95 (s, 1H, NH); ^{13}C NMR (125 MHz) *δ ppm*: 112.00, 112.52, 112.64, 112.74, 116.58, 120.26, 120.48, 120.52, 121.60, 122.36, 124.79, 124.99, 125.73, 126.15, 126.90, 126.96, 127.05, 128.50, 131.02, 131.98, 137.09, 137.56, 142.63, 160.84, 161.45, 162.98, 164.67; Anal. calcd. For $C_{17}H_{11}ClN_4O_2$: C, 60.28; H, 3.27; N, 16.54; Found C, 60.03; H, 3.31; N, 16.62.

N'-(5-Bromo-2-oxoindolin-3-ylidene)-1H-indole-2-carbohydrazide 6d.

Yield 85 %, m.p. > 300 °C (reported: > 300 °C [49]); Purity 96%; IR: 3433, 3344, 3161 (3NH), 1722, 1658 (2C=O); ^{1}H NMR (500 MHz) *δ ppm*: 6.87 (d, 1H, *J*= 8.5 Hz, Ar_H), 7.07 (t, 1H, *J*= 7.5 Hz, Ar_H), 7.25 (t, 1H, *J* = 7.5 Hz, Ar_H), 7.47 (d, 1H, *J* = 7.5 Hz, Ar_H), 7.56 (d, 1H, *J* = 7.5 Hz, Ar_H), 7.63 (s, 1H, Ar_H), 7.70 (d, 1H, *J* = 8.0 Hz, Ar_H), 8.31 (s, 1H, Ar_H), 11.00 (s, 1H, NH), 11.86 (s, 1H, NH), 11.95 (s, 1H, NH); Anal. calcd. For $C_{17}H_{11}BrN_4O_2$: C, 53.28; H, 2.89; N, 14.62; Found C, 53.13; H, 2.91; N, 14.72.

N'-(5-Methyl-2-oxoindolin-3-ylidene)-1H-indole-2-carbohydrazide 6e.

Yield 69 %, m.p. > 300 °C; Purity 99%; IR: 3455, 3329, 3140 (3NH), 1720, 1661 (2C=O); ^{1}H NMR (500 MHz) *δ ppm*: 2.31 (s, 3H, CH_3), 6.80 (d, 1H, *J*= 7.5 Hz, Ar_H), 7.07 (t, 1H, *J*= 8.0 Hz, Ar_H), 7.20 (d, 1H, *J*= 8.0 Hz, Ar_H), 7.24 (t, 1H, *J*= 7.5 Hz, Ar_H), 7.47 (d, 1H, *J*= 8.5 Hz, Ar_H), 7.61 (s, 1H, Ar_H), 7.70 (d, 1H, *J*= 8.0 Hz, Ar_H), 7.89 (s, 1H, Ar_H), 10.76 (s, 1H, NH), 11.62 (s, 1H, NH), 11.89 (s, 1H, NH); ^{13}C NMR (125 MHz) *δ ppm*: 20.58, 20.67 (CH_3), 110.42, 111.02, 112.51, 112.61, 115.60, 119.87, 120.22, 120.49, 121.32, 122.32, 124.66, 124.84, 126.97, 127.03, 127.07, 128.69, 130.73, 131.86, 132.12, 133.07, 137.06, 137.47, 140.12, 141.66, 160.75, 161.07, 163.24, 164.90; Anal. calcd. For $C_{18}H_{14}N_4O_2$: C, 67.92; H, 4.43; N, 17.60; Found C, 68.22; H, 4.40; N, 17.69.

N'-(5-Methoxy-2-oxoindolin-3-ylidene)-1H-indole-2-carbohydrazide 6f.

Yield 71 %, m.p. > 300 °C (reported: > 300 °C [49]); Purity 98%; IR: 3407, 3345, 3177 (3NH), 1722, 1664 (2C=O); ^{1}H NMR (500 MHz) *δ ppm*: 3.80 (s, 3H, OCH_3), 6.83 (d, 1H, *J*= 8.5 Hz, Ar_H), 6.99-7.02 (m, 1H, Ar_H), 7.06 (t, 1H, *J*= 8.0 Hz, Ar_H), 7.24 (t, 1H, *J*= 8.0 Hz, Ar_H), 7.46 (d, 1H, *J*= 8.5 Hz, Ar_H), 7.60 (s, 1H, Ar_H), 7.67 (brs, 1H, Ar_H), 7.70 (d, 1H, *J*= 7.5 Hz, Ar_H), 10.68 (s, 1H, NH), 11.80 (s, 1H, NH), 11.94 (s, 1H, NH); ^{13}C NMR (125 MHz) *δ ppm*: 55.65, 55.84 (OCH_3), 111.16, 112.11, 112.50, 112.65, 115.91, 118.21, 120.21, 120.50, 120.59, 122.32, 124.69, 124.87, 126.94, 127.05, 128.67, 136.02, 137.03, 137.50, 137.61, 154.49, 155.47, 160.45, 161.33, 163.29, 164.91; Anal. calcd. For $C_{18}H_{14}N_4O_3$: C, 64.67; H, 4.22; N, 16.76; Found C, 64.89; H, 4.18; N, 16.83.

N'-(2-Oxo-5-(trifluoromethoxy)indolin-3-ylidene)-1H-indole-2-carbohydrazide 6g.

Yield 78 %, m.p. > 300 °C; Purity 96%; IR: 3405, 3370, 3138 (3NH), 1728, 1653 (2C=O); ^{1}H NMR (500 MHz) *δ ppm*: 7.07-7.13 (m, 2H, Ar_H), 7.25-7.29 (m, 1H, Ar_H), 7.46 (t, 1H, *J*= 8.5 Hz, Ar_H), 7.65 (s, 1H, Ar_H), 7.71 (d, 1H, *J* = 8.0 Hz, Ar_H), 8.29-8.32 (m, 1H, Ar_H), 9.01 (s, 1H, Ar_H), 11.56 (s, 1H, NH), 11.97 (s, 1H, NH), 12.27 (s, 1H, NH); Anal. calcd. For $C_{18}H_{11}F_3N_4O_3$: C, 55.68; H, 2.86; N, 14.43; Found C, 55.83; H, 2.88; N, 14.54.

N'-(5-Nitro-2-oxoindolin-3-ylidene)-1H-indole-2-carbohydrazide 6h.

Yield 84 %, m.p. > 300 °C; Purity 97%; IR: 3418, 3337, 3141 (3NH), 1724, 1665 (2C=O); ^{1}H NMR (500 MHz) *δ ppm*: 6.98 (d, 1H, *J* = 8.5 Hz, Ar_H), 7.05-7.10 (m, 1H, Ar_H), 7.25 (t, 1H, *J*= 7.0 Hz, Ar_H), 7.41 (d, 1H, *J* = 9.5 Hz, Ar_H), 7.47 (d, 1H, *J* = 8.0 Hz, Ar_H), 7.64 (s, 1H, Ar_H), 7.70 (d, 1H, *J* = 8.5 Hz, Ar_H), 8.19 (s, 1H, Ar_H), 11.06 (s, 1H, NH), 11.94 (s, 2H, NH); ^{13}C NMR (125 MHz) *δ ppm*: 109.36, 112.53, 114.94, 120.27, 120.62, 122.25, 122.40, 123.31, 124.69, 126.43, 127.03, 128.74, 132.70, 137.15, 144.99, 161.73, 163.47; Anal. calcd. For $C_{17}H_{11}N_5O_4$: C, 58.46; H, 3.17; N, 20.05; Found C, 58.25; H, 3.30; N, 19.93.

N'-(5,7-Dimethyl-2-oxoindolin-3-ylidene)-1H-indole-2-carbohydrazide 6i.

Yield 86 %, m.p. > 300 °C; Purity 95%; IR: 3349, 3273, 3171 (3NH), 1720, 1668 (2C=O); ^{1}H NMR (500 MHz) *δ ppm*: 2.18 (s, 3H, CH_3), 2.29 (s, 3H, CH_3), 7.02-7.26 (m, 4H, Ar_H), 7.46 (d, 1H, *J*= 7.5 Hz, Ar_H), 7.60 (s, 1H, Ar_H), 7.70 (d, 1H, *J*= 8.0 Hz, Ar_H), 10.78 (s, 1H, NH), 11.56 (s, 1H, NH), 11.93 (s, 1H, NH); ^{13}C NMR (125 MHz) *δ ppm*: 16.03 (CH_3), 20.55 (CH_3), 112.49, 112.60, 115.32, 119.64, 120.19, 122.30, 124.41, 124.63, 127.06, 128.70, 130.60, 134.48, 137.03, 140.19, 163.72, 165.63; MS *m/z* [%]: 332.07 [M^+, 22.68], 144.13 [43.89], 89.10 [100]; Anal. calcd. For $C_{19}H_{16}N_4O_2$: C, 68.66; H, 4.85; N, 16.86; Found C, 68.49; H, 4.88; N, 16.76.

N'-(1-Methyl-2-oxoindolin-3-ylidene)-1H-indole-2-carbohydrazide 9a.

Yield 76 %, m.p. 259-261 °C (reported: 258-260 °C [49]); Purity 98%; IR: 3363, 3181 (2NH), 1722, 1659 (2C=O); ^{1}H NMR (500 MHz) *δ ppm*: 3.21 (s, 3H, *N*-CH_3), 7.07-7.13 (m, 2H, Ar_H), 7.16-7.19 (m, 1H, Ar_H), 7.25 (t, 1H, *J*= 8.0 Hz, Ar_H), 7.47-7.52 (m, 2H, Ar_H), 7.58 (s, 1H, Ar_H), 7.70 (d, 1H, *J*= 8.0 Hz, Ar_H), 8.06

(d, 1H, *J*= 7.5 Hz, Ar_H), 11.75 (s, 1H, NH), 11.96 (s, 1H, NH); ^{13}C NMR (125 MHz) *δ ppm*: 42.01 (*N*-CH_2), 109.87, 112.53, 115.18, 115.47, 115.64, 120.29, 122.28, 122.64, 124.74, 126.65, 127.04, 128.66, 129.42, 129.49, 129.66, 132.45, 132.56, 137.17, 143.70, 160.56, 161.07, 162.49, 163.65; MS *m/z* [%]: 318.20 [M^+, 24.32], 144.16 [67.97], 89.14 [100]; Anal. calcd. For $C_{18}H_{14}N_4O_2$: C, 67.92; H, 4.43; N, 17.60; Found C, 67.73; H, 4.46; N, 17.69.

N'-(2-Oxo-1-propylindolin-3-ylidene)-1H-indole-2-carbohydrazide 9b.

Yield 69 %, m.p. 229-231 °C; Purity 99%; IR: 3450, 3284 (2NH), 1716, 1681 (2C=O); ^{1}H NMR (500 MHz) *δ ppm*: 0.88 (t, 3H, *J*= 7.5 Hz, -CH_2-C<u>H</u>3), 1.60-1.67 (m, 2H, -C<u>H2</u>-CH_3), 3.70 (t, 2H, *J* = 7.5 Hz, *N*-CH_2), 7.08 (t, 1H, *J*= 7.0 Hz, Ar_H), 7.15-7.19 (m, 2H, Ar_H), 7.25 (t, 1H, *J*= 8.5 Hz, Ar_H), 7.47-7.50 (m, 2H, Ar_H), 7.59 (s, 1H, Ar_H), 7.70 (d, 1H, *J*= 8.5 Hz, Ar_H), 8.07 (d, 1H, *J*= 8.0 Hz, Ar_H), 11.74 (s, 1H, NH), 11.96 (s, 1H, NH); ^{13}C NMR (125 MHz) *δ ppm*: 11.17 (-CH_2-<u>C</u>H3), 20.40 (*N*-CH_2-<u>C</u>H2), 40.87 (*N*-CH_2), 109.55, 112.52, 114.99, 120.27, 122.26, 122.30, 124.70, 126.60, 127.04, 128.70, 132.69, 137.15, 144.33, 160.85, 163.47; Anal. calcd. For $C_{20}H_{18}N_4O_2$: C, 69.35; H, 5.24; N, 16.17; Found C, 69.08; H, 5.29; N, 16.08.

Ethyl 2-(3-(2-(1H-indole-2-carbonyl)hydrazono)-2-oxoindolin-1-yl)acetate 9c.

Yield 81 %, m.p. 261-263 °C; Purity 96%; IR: 3347, 3245 (2NH), 1726, 1687 (2C=O); ^{1}H NMR (500 MHz) *δ ppm*: 1.21 (t, 3H*J* = 7.0 Hz, CH_2-C<u>H3</u>,), 4.17 (q, 2H, *J* = 7.0 Hz, C<u>H2</u>-CH_3), 4.74 (s, 2H, *N*-CH_2-CO), 7.09 (t, 1H, *J*= 8.0 Hz, Ar_H), 7.20-7.23 (m, 3H, Ar_H), 7.26 (t, 1H, *J*= 8.0 Hz, Ar_H), 7.45 (t, 2H, *J*= 8.0 Hz, Ar_H), 7.71 (t, 2H, *J*= 8.0 Hz, Ar_H), 12.15 (s, 1H, NH), 13.69 (s, 1H, NH); ^{13}C NMR (125 MHz) *δ ppm*: 14.04 (CH_3), 40.96 (*N*-CH_2), 61.47 (<u>C</u>H2-CH_3), 110.38, 112.61, 119.03, 120.52, 120.80, 122.35, 123.62, 124.98, 126.95, 128.59, 131.63, 135.59, 137.55, 142.59, 161.25, 167.48; Anal. calcd. For $C_{21}H_{18}N_4O_4$: C, 64.61; H, 4.65; N, 14.35; Found C, 64.78; H, 4.67; N, 14.24.

N'-(1-Benzyl-2-oxoindolin-3-ylidene)-1H-indole-2-carbohydrazide 9d.

Yield 81 %, m.p. 253-255 °C (reported: 249-251 °C [49]); Purity 98%; IR: 3453, 3368 (2NH), 1717, 1665 (2C=O); ^{1}H NMR (500 MHz) *δ ppm*: 5.00 (s, 2H, benzylic H), 7.04 (d, 1H, *J*= 8.5 Hz, Ar_H), 7.08 (t, 1H, *J*= 7.5 Hz, Ar_H), 7.14 (t, 1H, *J*= 7.5 Hz, Ar_H), 7.25-7.28 (m, 1H, Ar_H), 7.32-7.36 (m, 5H, Ar_H), 7.40 (t, 1H, *J*= 7.5 Hz, Ar_H), 7.47 (d, 1H, *J*= 8.5 Hz, Ar_H), 7.62 (s, 1H, Ar_H), 7.70 (d, 1H, *J*= 8.5 Hz, Ar_H), 8.10 (d, 1H, *J*= 7.5 Hz, Ar_H), 11.79 (s, 1H, NH), 11.98 (s, 1H, NH); MS *m/z* [%]: 394.06 [M^+, 66.21], 144.06 [27.98], 91.13 [100]; Anal. calcd. For $C_{24}H_{18}N_4O_2$: C, 73.08; H, 4.60; N, 14.20; Found C, 72.71; H, 4.63; N, 14.28.

N'-(1-(4-Fluorobenzyl)-2-oxoindolin-3-ylidene)-1H-indole-2-carbohydrazide 9e.

Yield 84 %, m.p. 233-235 °C; Purity 97%; IR: 3448, 3378 (2NH), 1714, 1663 (2C=O); ^{1}H NMR (500 MHz) *δ ppm*: 4.99 (s, 2H, benzylic H), 7.07-7.09 (m, 2H, Ar_H), 7.15-7.19 (m, 3H, Ar_H), 7.25 (t, 1H, *J*= 7.0 Hz, Ar_H), 7.40-7.44 (m, 3H, Ar_H), 7.47 (d, 1H, *J*= 8.0 Hz, Ar_H), 7.61 (s, 1H, Ar_H), 7.70 (d, 1H, *J*= 8.0 Hz, Ar_H), 8.10 (d, 1H, *J*= 7.5 Hz, Ar_H), 11.77 (s, 1H, NH), 11.97 (s, 1H, NH); ^{13}C NMR (125 MHz) *δ ppm*: 41.95, 42.01, 109.85, 112.53, 115.18, 115.46, 115.63, 119.54, 120.28, 120.58, 120.89, 122.27, 122.63, 123.41, 124.73, 124.91, 126.63, 127.04, 128.65, 129.41, 129.48, 129.64, 129.71, 131.75, 132.46, 132.54, 137.17, 143.70, 160.56, 160.83, 162.48, 163.64; MS *m/z* [%]: 412.14 [M^+, 44.30], 109.03 [100]; Anal. calcd. For $C_{24}H_{17}FN_4O_2$: C, 69.89; H, 4.15; N, 13.59; Found C, 70.23; H, 4.17; N, 13.68.

N'-(5-Chloro-2-oxo-1-propylindolin-3-ylidene)-1H-indole-2-carbohydrazide 9f.

Yield 73 %, m.p. 242-243 °C; Purity 99%; IR: 3450, 3284 (2NH), 1716, 1681 (2C=O); ^{1}H NMR (500 MHz) *δ ppm*: 0.84 (t, 3H, *J*= 6.8 Hz, -CH_2-C<u>H3</u>), 1.57-1.60 (m, 2H, -C<u>H2</u>-CH_3), 3.64 (t, 2H, *J*= 6.8 Hz, *N*-CH_2), 6.99 (t, 1H, *J*= 7.2 Hz, Ar_H), 7.08 (t, 1H, *J*= 7.2 Hz, Ar_H), 7.16 (s, 1H, Ar_H), 7.37-7.60 (m, 5H, Ar_H), 10.99 (s, 1H, NH), 12.39 (s, 1H, NH); ^{13}C NMR (125 MHz) *δ ppm*: 11.55 (-CH_2-<u>C</u>H3), 20.78 (*N*-CH_2-<u>C</u>H2), 41.29 (*N*-CH_2),

107.29, 111.95, 112.05, 119.00, 120.49, 121.45, 121.63, 125.01, 127.69, 131.05, 136.57, 142.02, 160.83; Anal. calcd. For $C_{20}H_{17}Cl N_4O_2$: C, 63.08; H, 4.50; N, 14.71; Found C, 63.27; H, 4.53; N, 14.63.

N'-(1,3-Dihydro-2H-inden-2-ylidene)-1H-indole-2-carbohydrazide 11a.

Yield 79 %, m.p. 219-212 °C; Purity 97%; IR: 3358, 3264 (2NH), 1689 (C=O); ^{1}H NMR (400 MHz) *δ ppm*: 3.41 (s, 4H, 2 CH_2), 7.06 (t, 1H, *J*= 8.5 Hz, Ar_H), 7.20 (t, 1H, *J*= 8.5 Hz, Ar_H), 7.33 (d, 2H, *J*= 8.0 Hz, Ar_H), 7.37 (s, 1H, Ar_H), 7.41 (t, 2H, *J*= 8.0 Hz, Ar_H), 7.45 (d, 1H, *J*= 8.5 Hz, Ar_H), 7.65 (d, 1H, *J*= 8.5 Hz, Ar_H), 10.74 (s, 1H, NH), 11.92 (s, 1H, NH); Anal. calcd. For $C_{18}H_{15}N_3O$: C, 74.72; H, 5.23; N, 14.52; Found C, 75.11; H, 5.18; N, 14.47.

N'-(3,4-Dihydronaphthalen-1(2H)-ylidene)-1H-indole-2-carbohydrazide 11b.

Yield 74 %, m.p. 195-197 °C; Purity 98%; IR: 3329, 3283 (2NH), 1691 (C=O); ^{1}H NMR (400 MHz) *δ ppm*: 1.84 (q, 2H, *J* = 6.5 Hz, CH_2-C$\underline{H_2}$-CH_2), 2.78 (t, 4H, *J* = 6.5 Hz, C$\underline{H_2}$-CH_2-C$\underline{H_2}$), 7.04 (t, 1H, *J*= 8.0 Hz, Ar_H), 7.20 (t, 2H, *J*= 7.5 Hz, Ar_H), 7.28-7.33 (m, 2H, Ar_H), 7.39 (brs, 1H, Ar_H), 7.45 (d, 1H, *J*= 8.5 Hz, Ar_H), 7.65 (d, 1H, *J*= 8.5 Hz, Ar_H), 8.09 (d, 1H, *J*= 7.5 Hz, Ar_H), 10.63 (s, 1H, NH), 11.76 (s, 1H, NH); ^{13}C NMR (100 MHz) *δ ppm*: 21.44, 26.03, 28.93, 112.36, 119.88, 121.80, 123.84, 124.72, 126.35, 127.06, 128.70, 129.38, 132.31, 136.71, 140.18; MS *m/z* [%]: 303.13 [M^+, 100], 144.18 [42.85]; Anal. calcd. For $C_{19}H_{17}N_3O$: C, 75.23; H, 5.65; N, 13.85; Found C, 75.32; H, 5.69; N, 13.77.

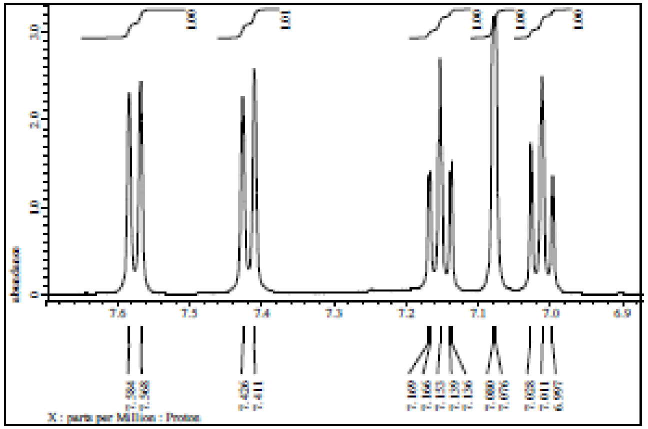
X : parts per Million : Proton
7.584
7.568
7.426
7.411

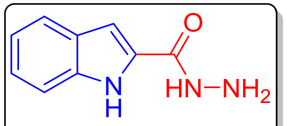
O
N
H
HN–NH2

4

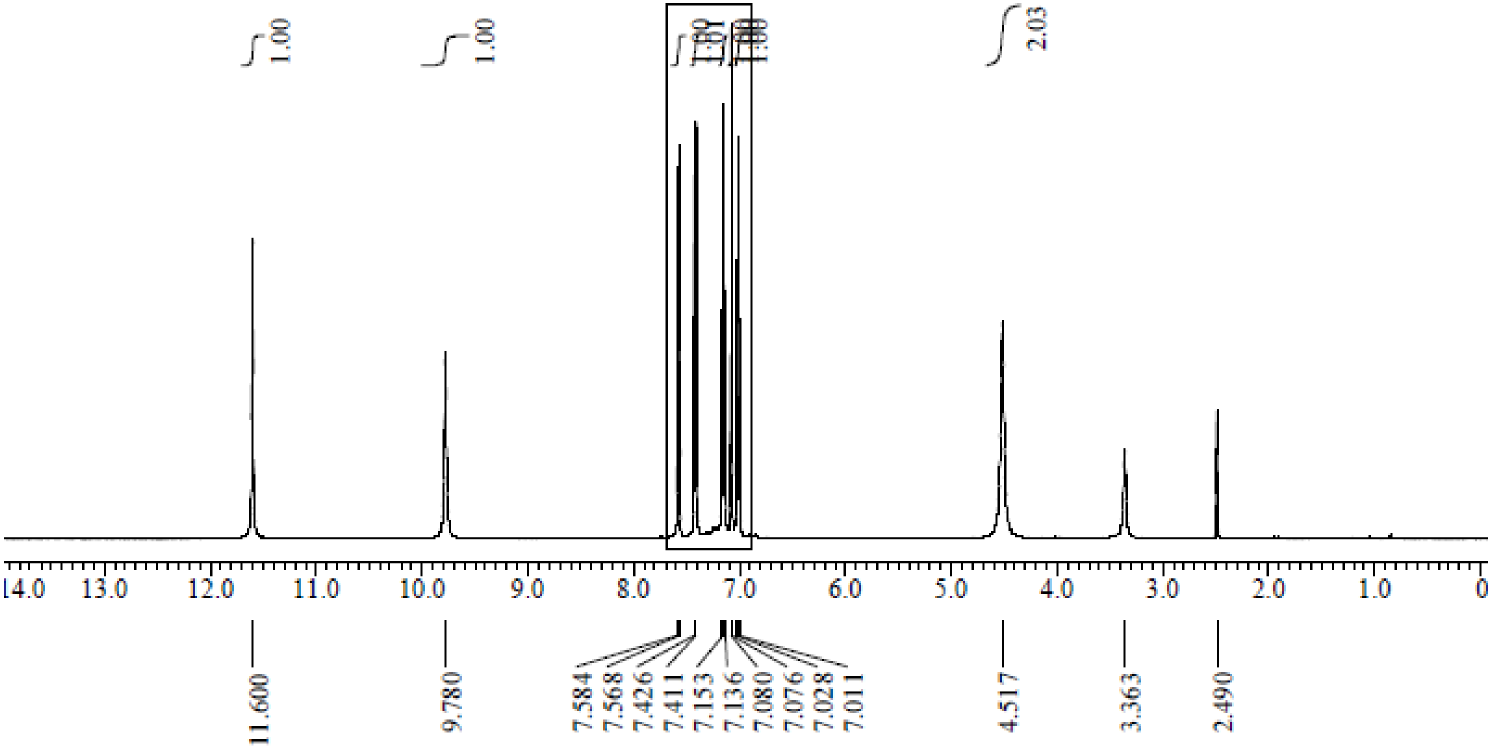
1.00
1.00
2.03
14.0
13.0
12.0
11.0
10.0
9.0
8.0
7.0
6.0
5.0
4.0
3.0
2.0
1.0
0
11.600
9.780
7.584
7.568
7.426
7.411
7.153
7.136
7.080
7.076
7.028
7.011
4.517
3.363
2.490

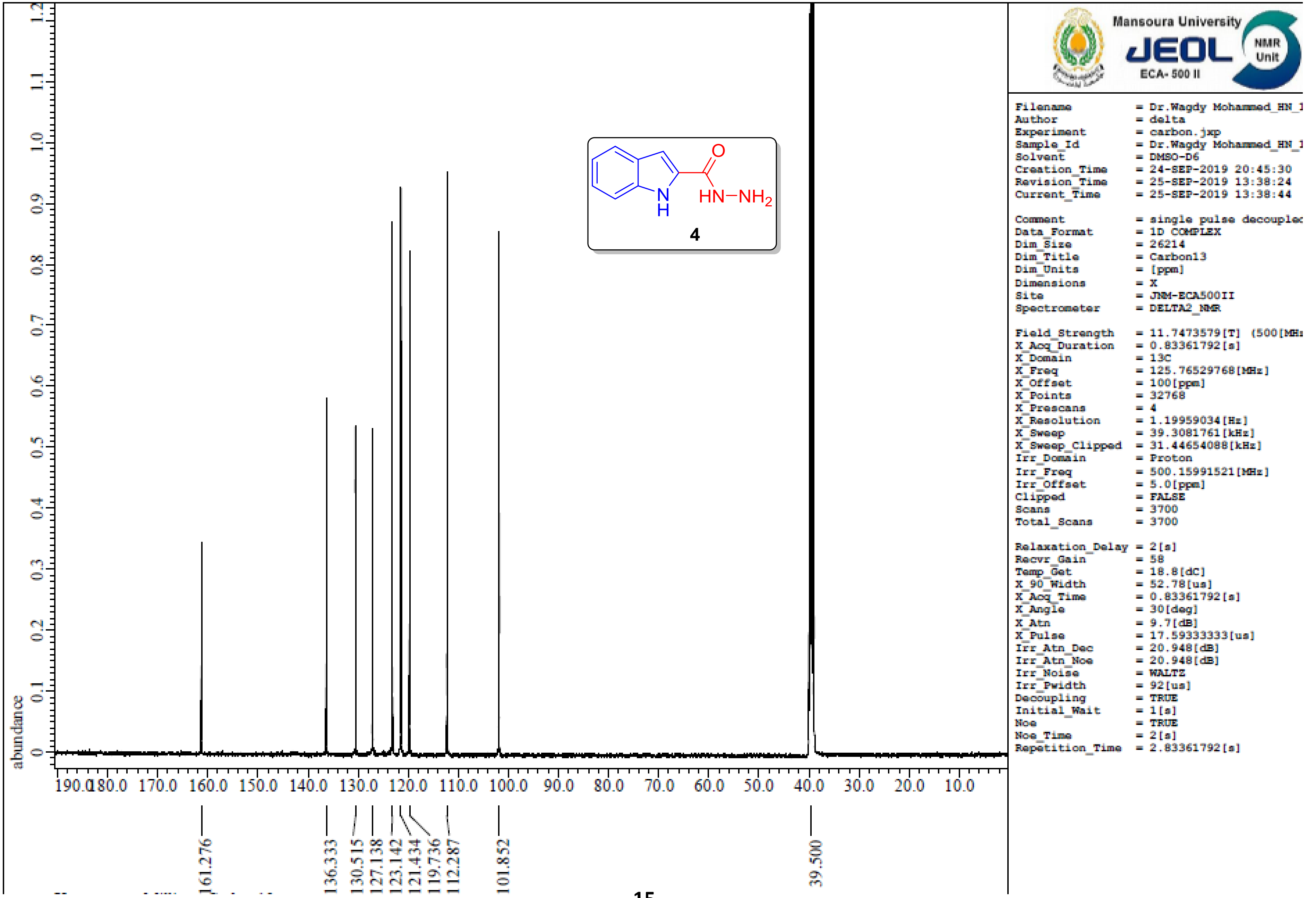
Mansoura University
JEOL
ECA- 500 II
NMR Unit
HN–NH2
O
N
H
4
abundance
Filename = Dr.Wagdy Mohammed_HN_
Author = delta
Experiment = carbon.jxp
Sample_Id = Dr.Wagdy Mohammed_HN_
Solvent = DMSO-D6
Creation_Time = 24-SEP-2019 20:45:30
Revision_Time = 25-SEP-2019 13:38:24
Current_Time = 25-SEP-2019 13:38:44
Comment = single pulse decouple
Data_Format = 1D COMPLEX
Dim_Size = 26214
Dim_Title = Carbon13
Dim_Units = [ppm]
Dimensions = X
Site = JNM-ECA500II
Spectrometer = DELTA2_NMR
Field_Strength = 11.7473579[T] (500[MH
X_Acq_Duration = 0.83361792[s]
X_Domain = 13C
X_Freq = 125.76529768[MHz]
X_Offset = 100[ppm]
X_Points = 32768
X_Prescans = 4
X_Resolution = 1.19959034[Hz]
X_Sweep = 39.3081761[kHz]
X_Sweep_Clipped = 31.44654088[kHz]
Irr_Domain = Proton
Irr_Freq = 500.15991521[MHz]
Irr_Offset = 5.0[ppm]
Clipped = FALSE
Scans = 3700
Total_Scans = 3700
Relaxation_Delay = 2[s]
Recvr_Gain = 58
Temp_Get = 18.8[dC]
X_90_Width = 52.78[us]
X_Acq_Time = 0.83361792[s]
X_Angle = 30[deg]
X_Atn = 9.7[dB]
X_Pulse = 17.59333333[us]
Irr_Atn_Dec = 20.948[dB]
Irr_Atn_Noe = 20.948[dB]
Irr_Noise = WALTZ
Irr_Pwidth = 92[us]
Decoupling = TRUE
Initial_Wait = 1[s]
Noe = TRUE
Noe_Time = 2[s]
Repetition_Time = 2.83361792[s]
161.276
136.333
130.515
127.138
123.142
121.434
119.736
112.287
101.852
39.500

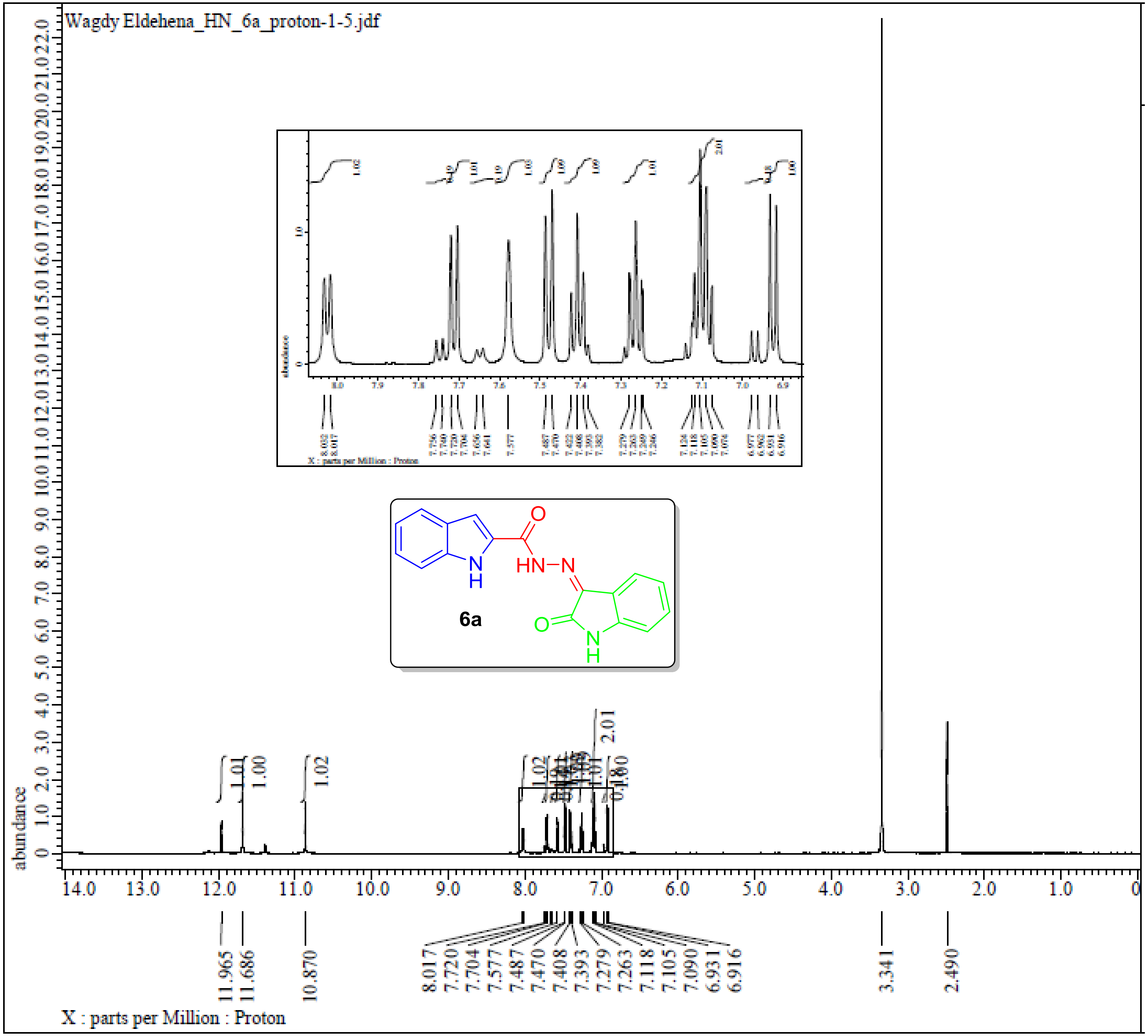
Wagdy Eldehena_HN_6a_proton-1-5.jdf
abundance
X : parts per Million : Proton
11.965
11.686
10.870
8.017
7.720
7.704
7.577
7.487
7.470
7.408
7.393
7.279
7.263
7.118
7.105
7.090
6.931
6.916
3.341
2.490
1.01
1.00
1.02
2.01
6a
HN
N
NH
O

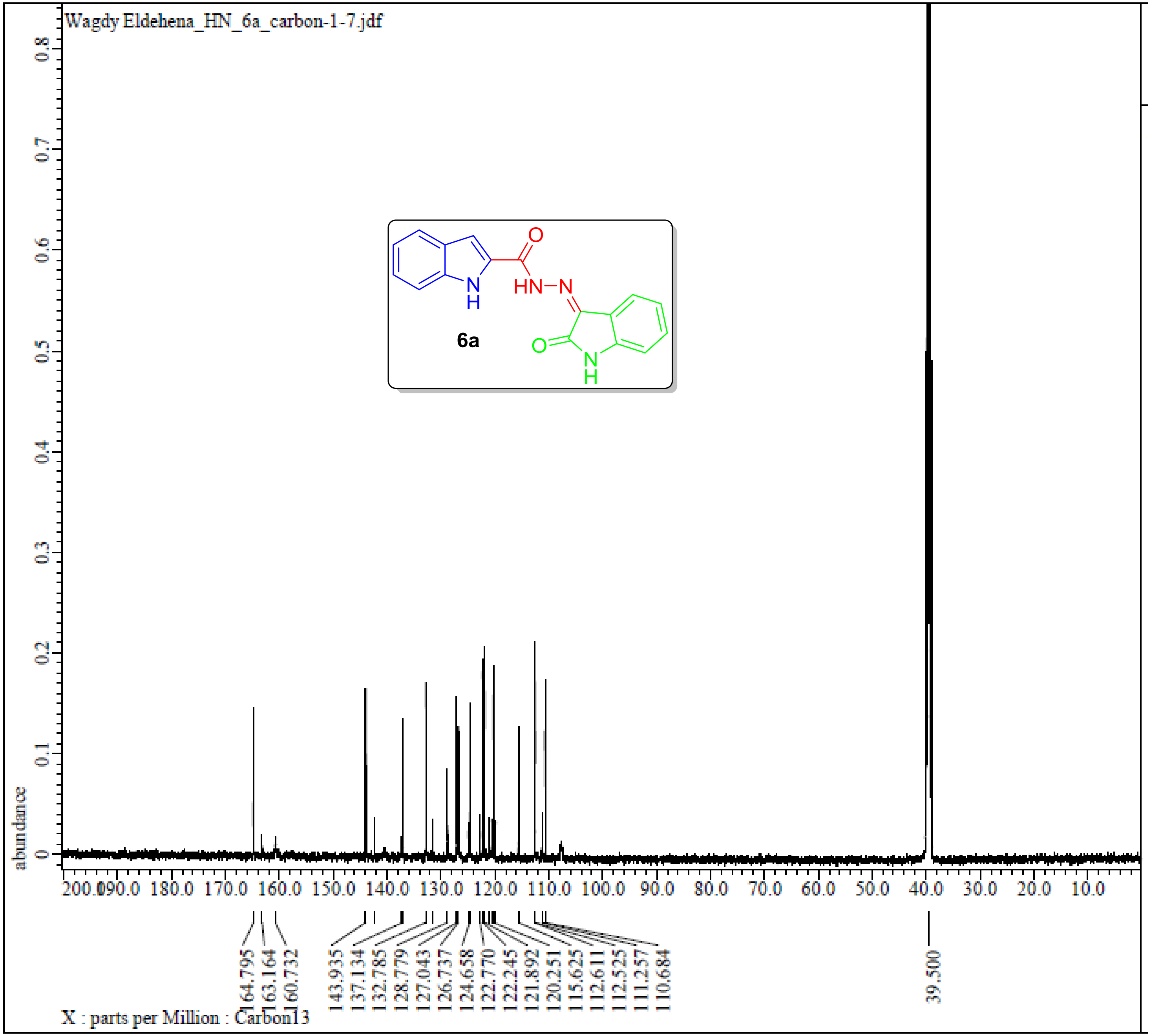
Wagdy Eldehena_HN_6a_carbon-1-7.jdf
6a
abundance
X : parts per Million : Carbon13
164.795
163.164
160.732
143.935
137.134
132.785
128.779
127.043
126.737
124.658
122.770
122.245
121.892
120.251
115.625
112.611
112.525
111.257
110.684
39.500

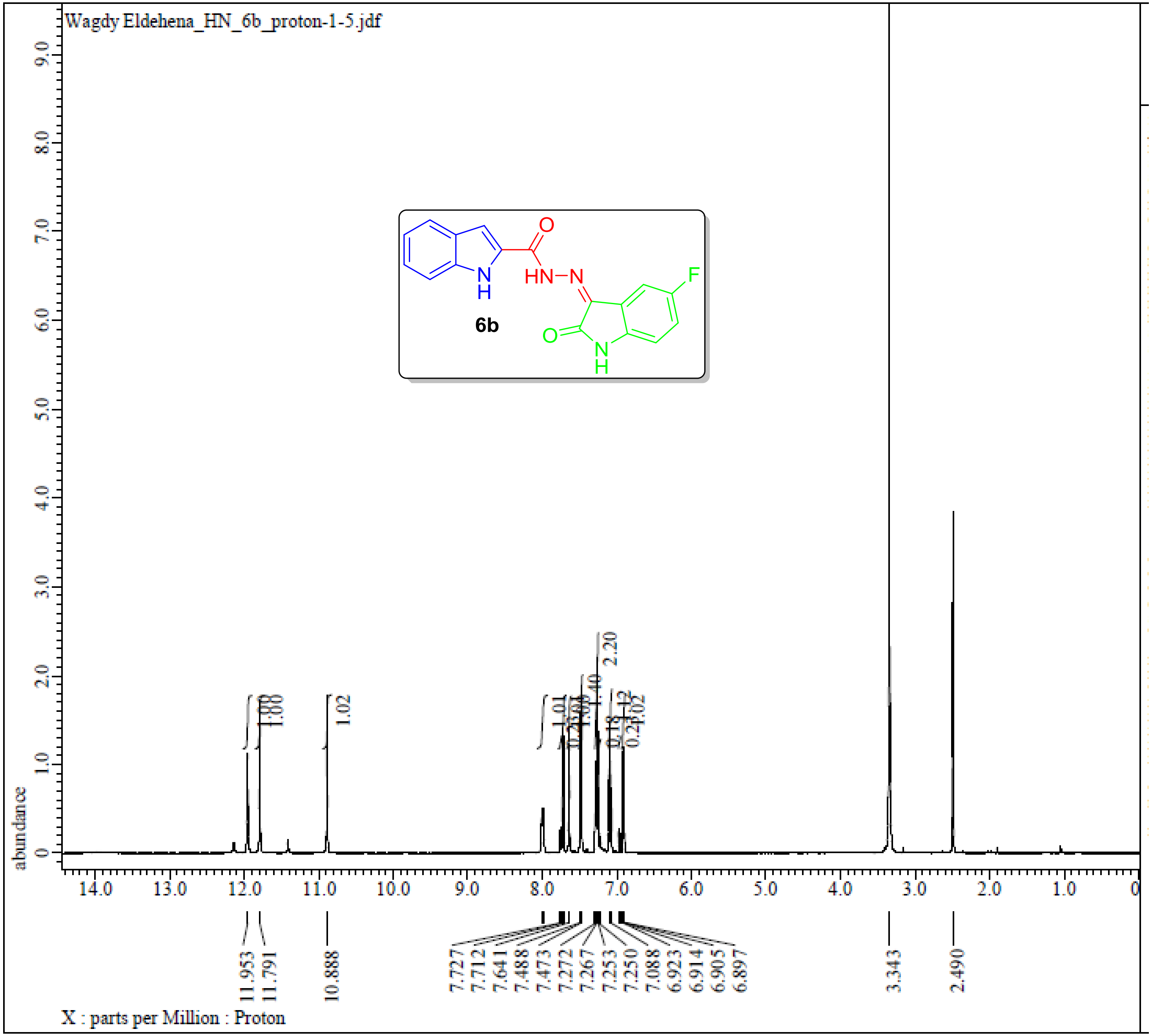
Wagdy Eldehena_HN_6b_proton-1-5.jdf
6b
abundance
X : parts per Million : Proton
11.953
11.791
10.888
7.727
7.712
7.641
7.488
7.473
7.272
7.267
7.253
7.250
7.088
6.923
6.914
6.905
6.897
3.343
2.490
1.02
2.20

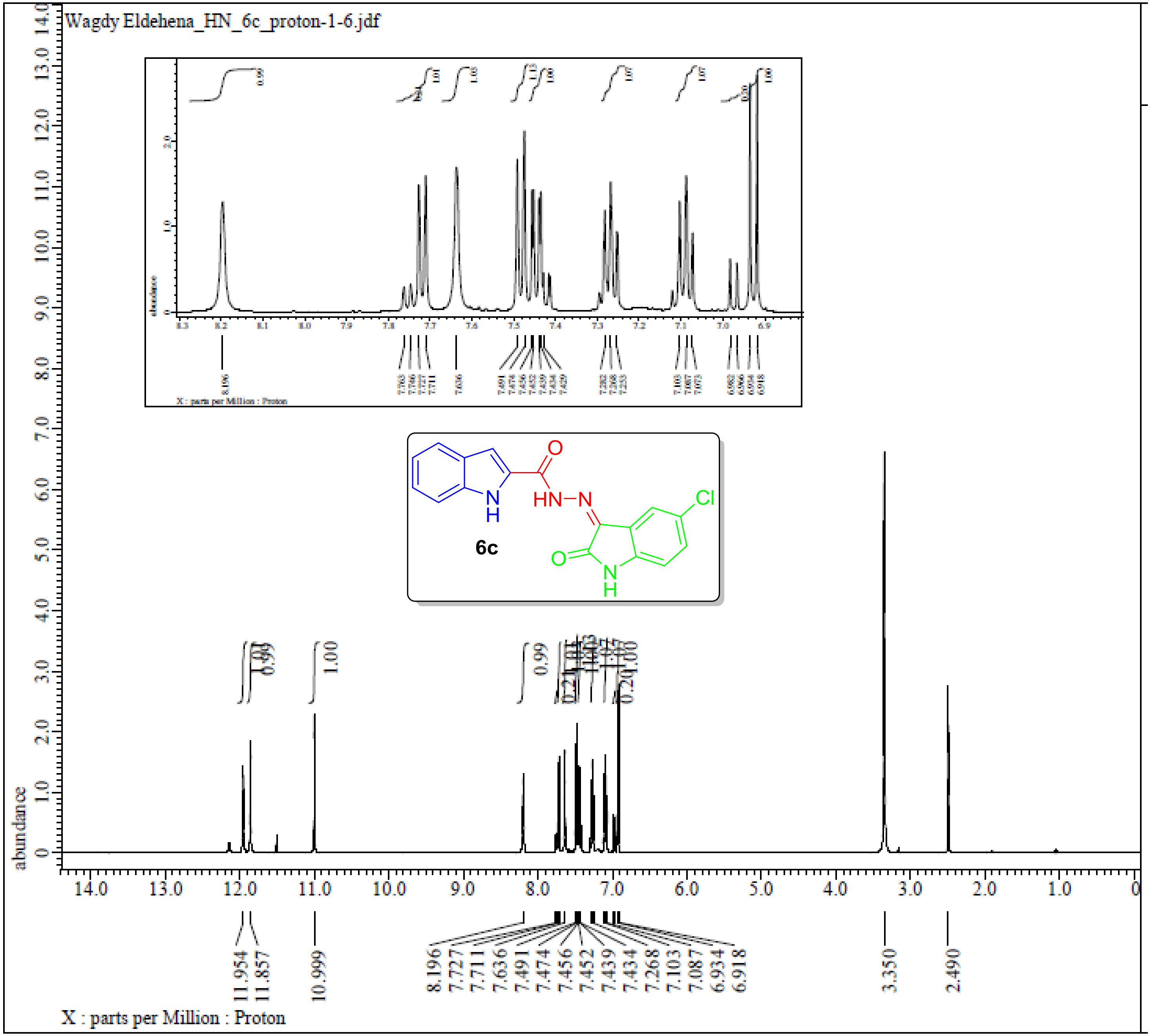
Wagdy Eldehena_HN_6c_proton-1-6.jdf
6c
O
HN−N
N
H
Cl
O
N
H
abundance
X : parts per Million : Proton
11.954
11.857
10.999
8.196
7.727
7.711
7.636
7.491
7.474
7.456
7.452
7.439
7.434
7.268
7.103
7.087
6.934
6.918
3.350
2.490
1.00
0.99

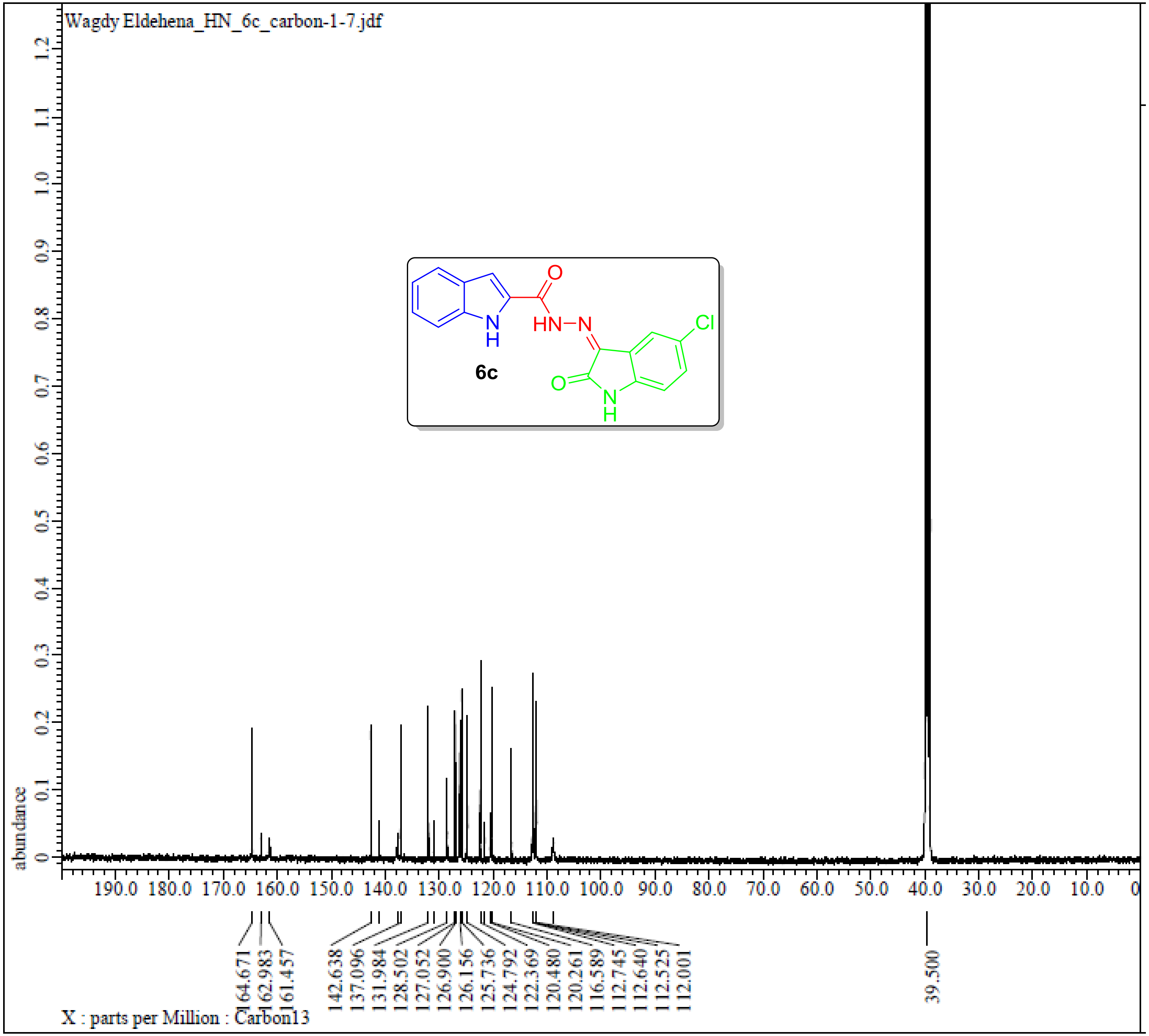
Wagdy Eldehena_HN_6c_carbon-1-7.jdf
6c
X : parts per Million : Carbon13
abundance
164.671
162.983
161.457
142.638
137.096
131.984
128.502
127.052
126.900
126.156
125.736
124.792
122.369
120.480
120.261
116.589
112.745
112.640
112.525
112.001
39.500

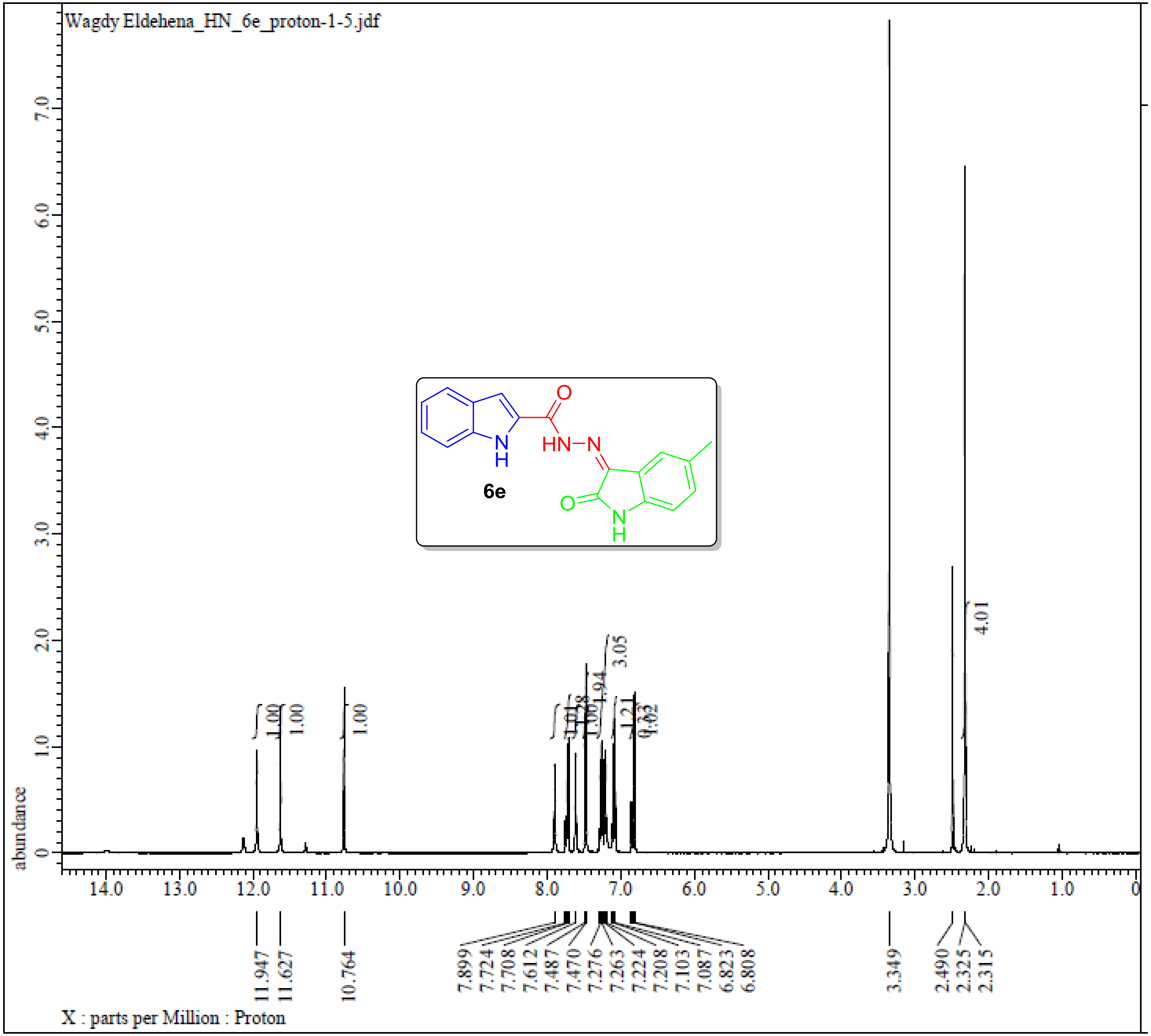
Wagdy Eldehena_HN_6e_proton-1-5.jdf
abundance
X : parts per Million : Proton
6e
1.00
1.00
1.00
3.05
1.94
1.21
4.01
11.947
11.627
10.764
7.899
7.724
7.708
7.612
7.487
7.470
7.276
7.263
7.224
7.208
7.103
7.087
6.823
6.808
3.349
2.490
2.325
2.315

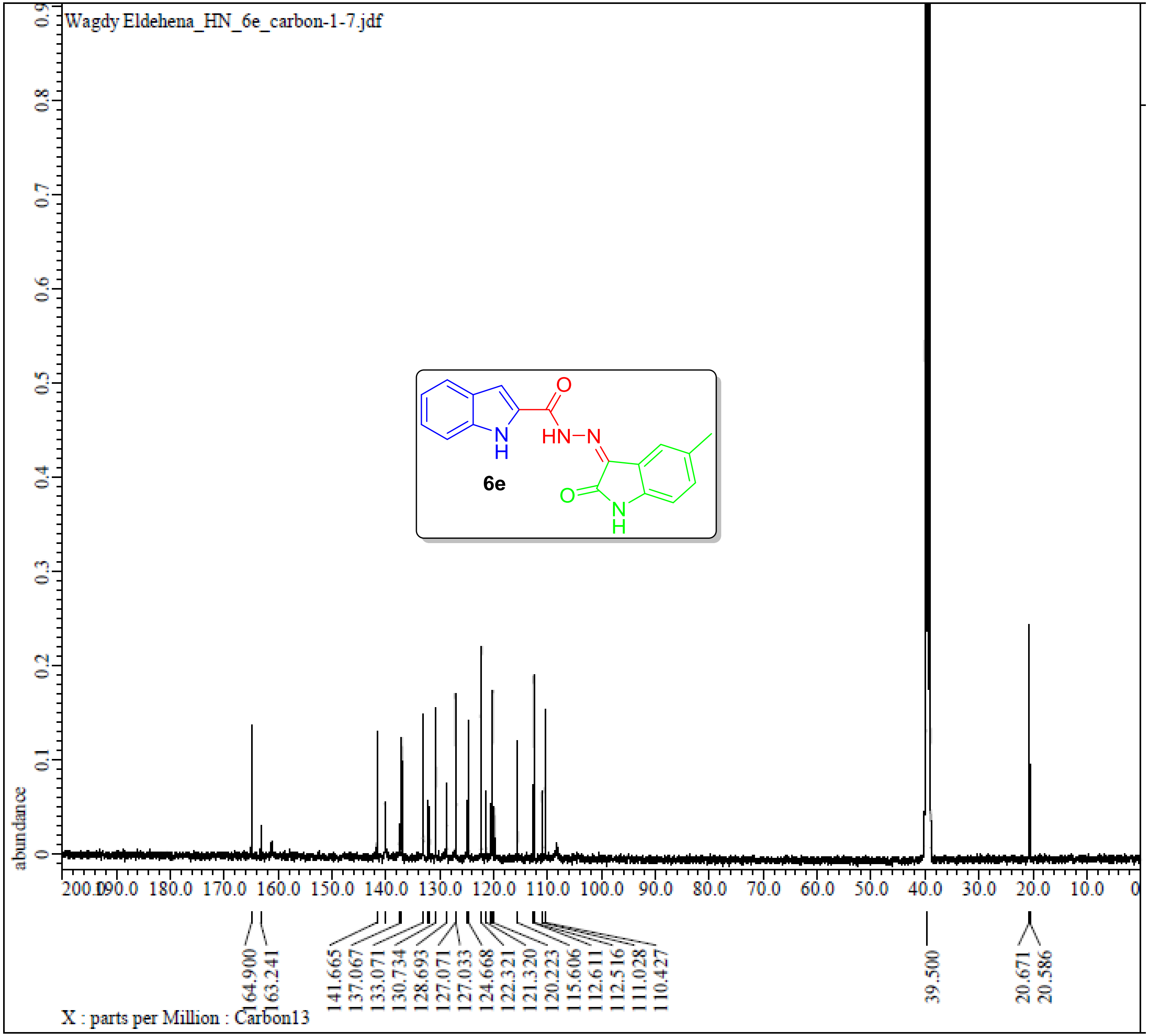
Wagdy Eldehena_HN_6e_carbon-1-7.jdf
abundance
X : parts per Million : Carbon13
6e
O
HN–N
N
H
164.900
163.241
141.665
137.067
133.071
130.734
128.693
127.071
127.033
124.668
122.321
121.320
120.223
115.606
112.611
112.516
111.028
110.427
39.500
20.671
20.586

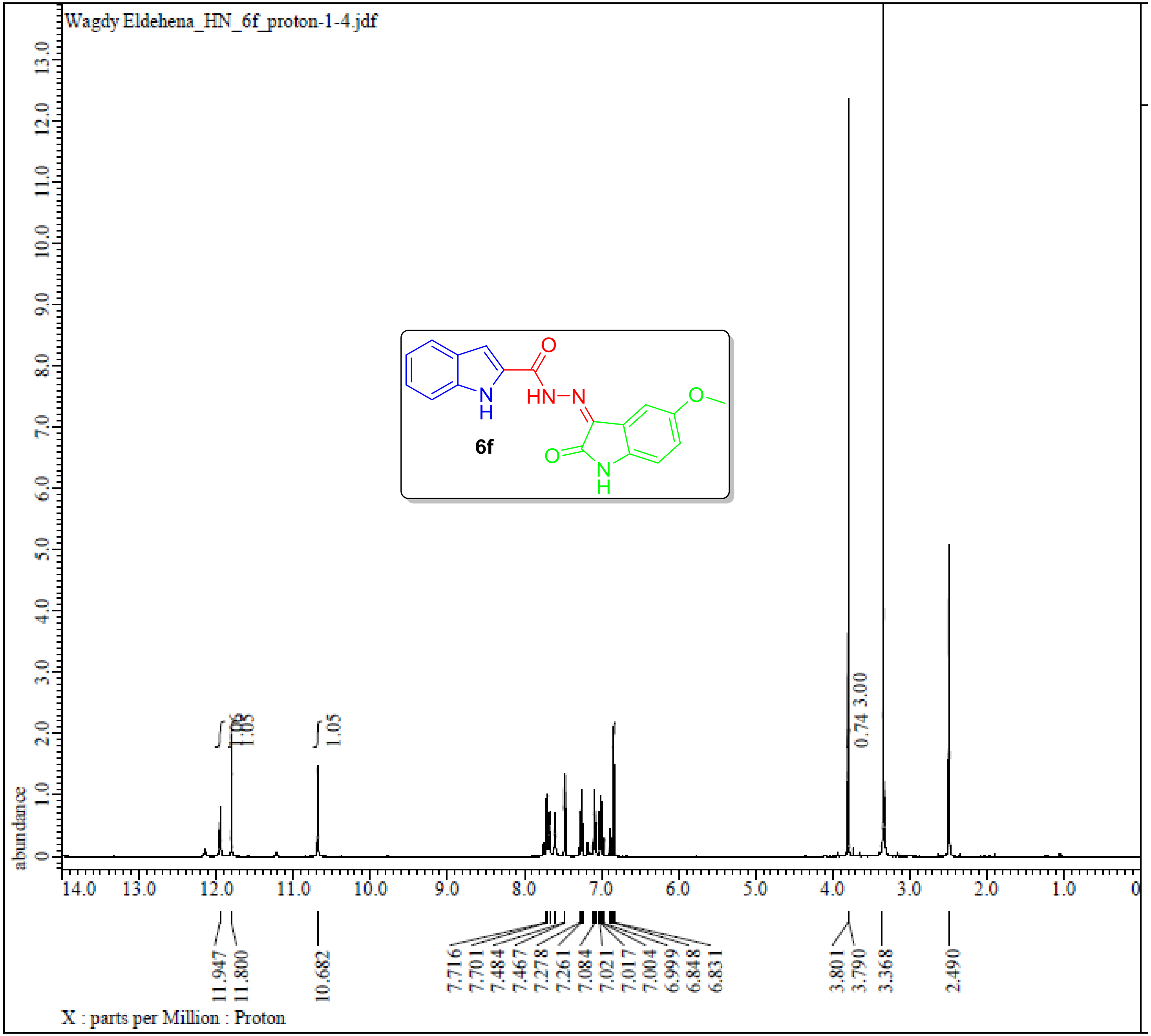
Wagdy Eldehena_HN_6f_proton-1-4.jdf
6f
abundance
X : parts per Million : Proton
1.05
0.74
3.00
11.947
11.800
10.682
7.716
7.701
7.484
7.467
7.278
7.261
7.084
7.021
7.017
7.004
6.999
6.848
6.831
3.801
3.790
3.368
2.490

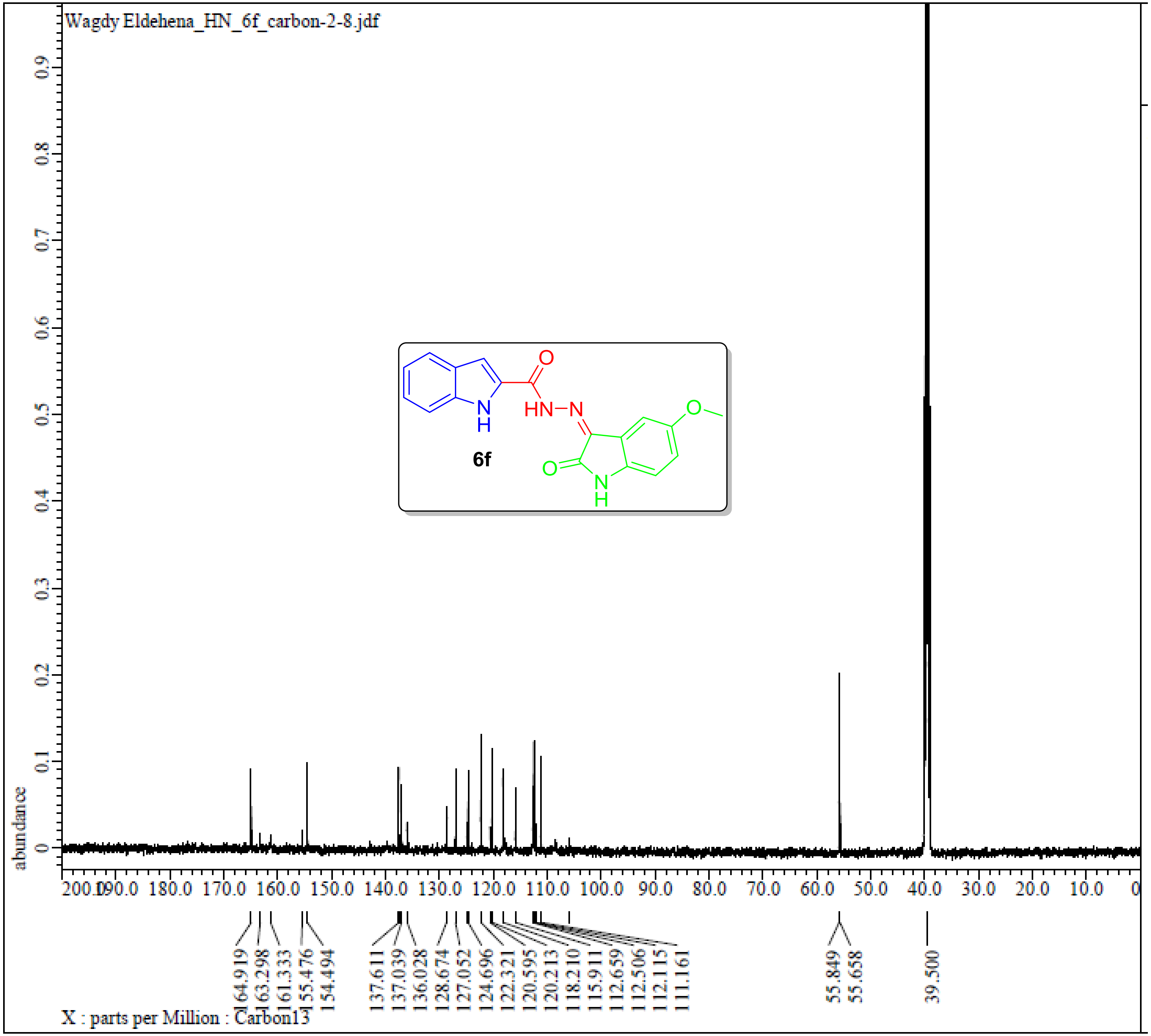
Wagdy Eldehena_HN_6f_carbon-2-8.jdf
abundance
0 0.1 0.2 0.3 0.4 0.5 0.6 0.7 0.8 0.9
200.0 190.0 180.0 170.0 160.0 150.0 140.0 130.0 120.0 110.0 100.0 90.0 80.0 70.0 60.0 50.0 40.0 30.0 20.0 10.0 0
X : parts per Million : Carbon13
164.919
163.298
161.333
155.476
154.494
137.611
137.039
136.028
128.674
127.052
124.696
122.321
120.595
120.213
118.210
115.911
112.659
112.506
112.115
111.161
55.849
55.658
39.500
O
HN–N
N
H
O
O
N
H
6f

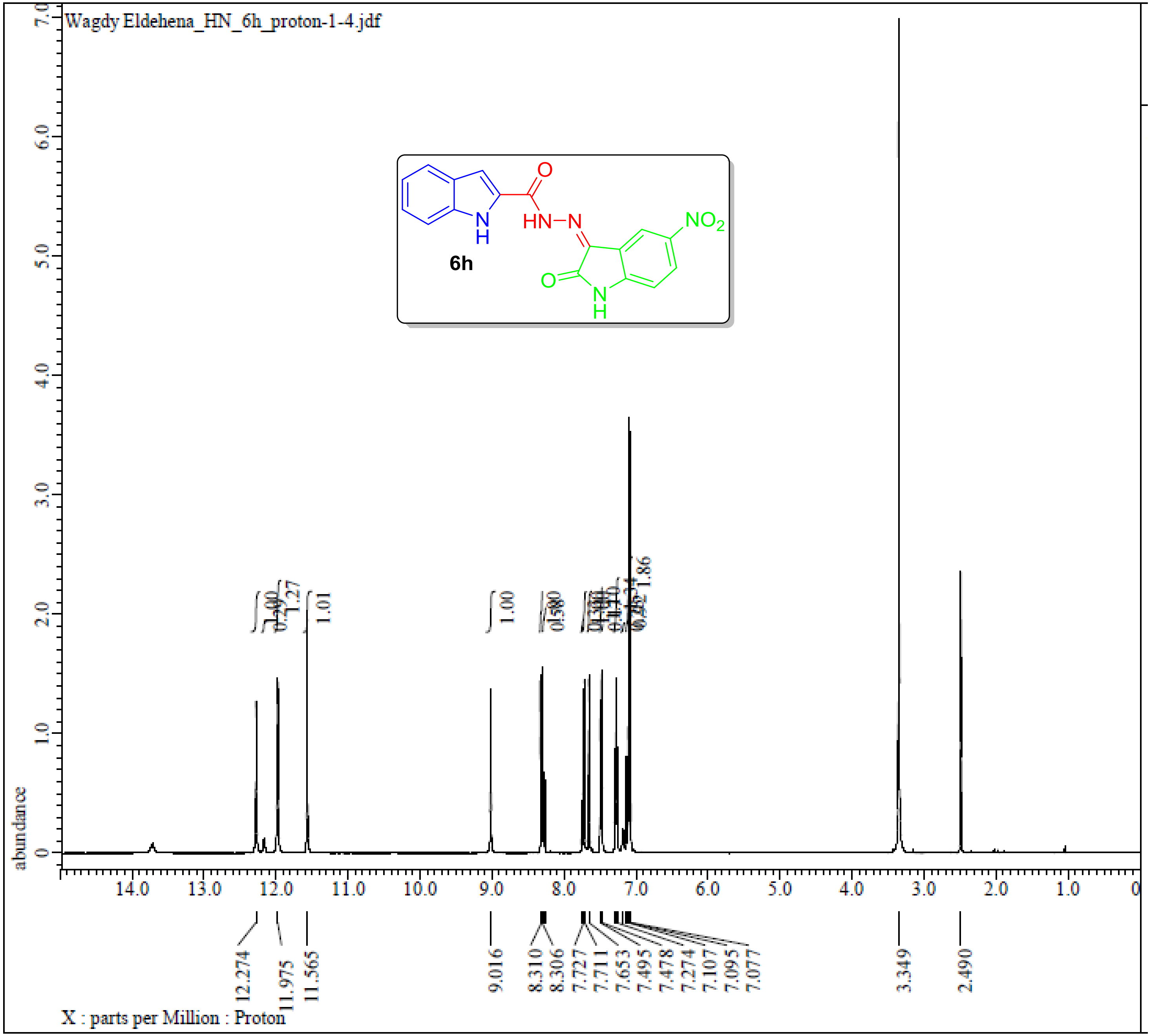
Wagdy Eldehena_HN_6h_proton-1-4.jdf
abundance
X : parts per Million : Proton
O
HN–N
NO_2
N
H
6h
12.274
11.975
11.565
9.016
8.310
8.306
7.727
7.711
7.653
7.495
7.478
7.274
7.107
7.095
7.077
3.349
2.490
1.27
1.01
1.00
1.86

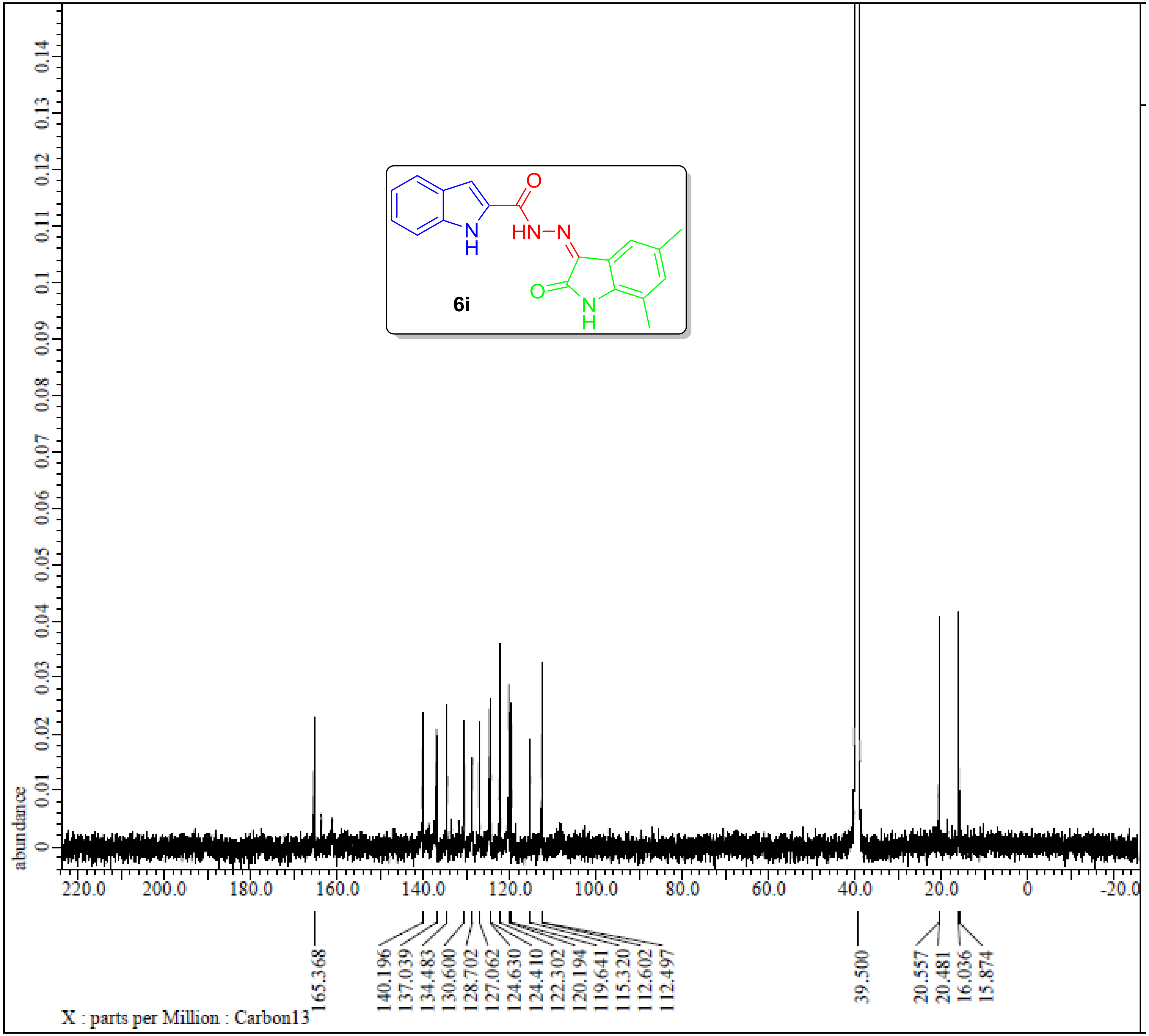
6i
O
HN–N
N
H
O
N
H
abundance
0
0.01
0.02
0.03
0.04
0.05
0.06
0.07
0.08
0.09
0.1
0.11
0.12
0.13
0.14
220.0
200.0
180.0
160.0
140.0
120.0
100.0
80.0
60.0
40.0
20.0
0
-20.0
165.368
140.196
137.039
134.483
130.600
128.702
127.062
124.630
124.410
122.302
120.194
119.641
115.320
112.602
112.497
39.500
20.557
20.481
16.036
15.874
X : parts per Million : Carbon13

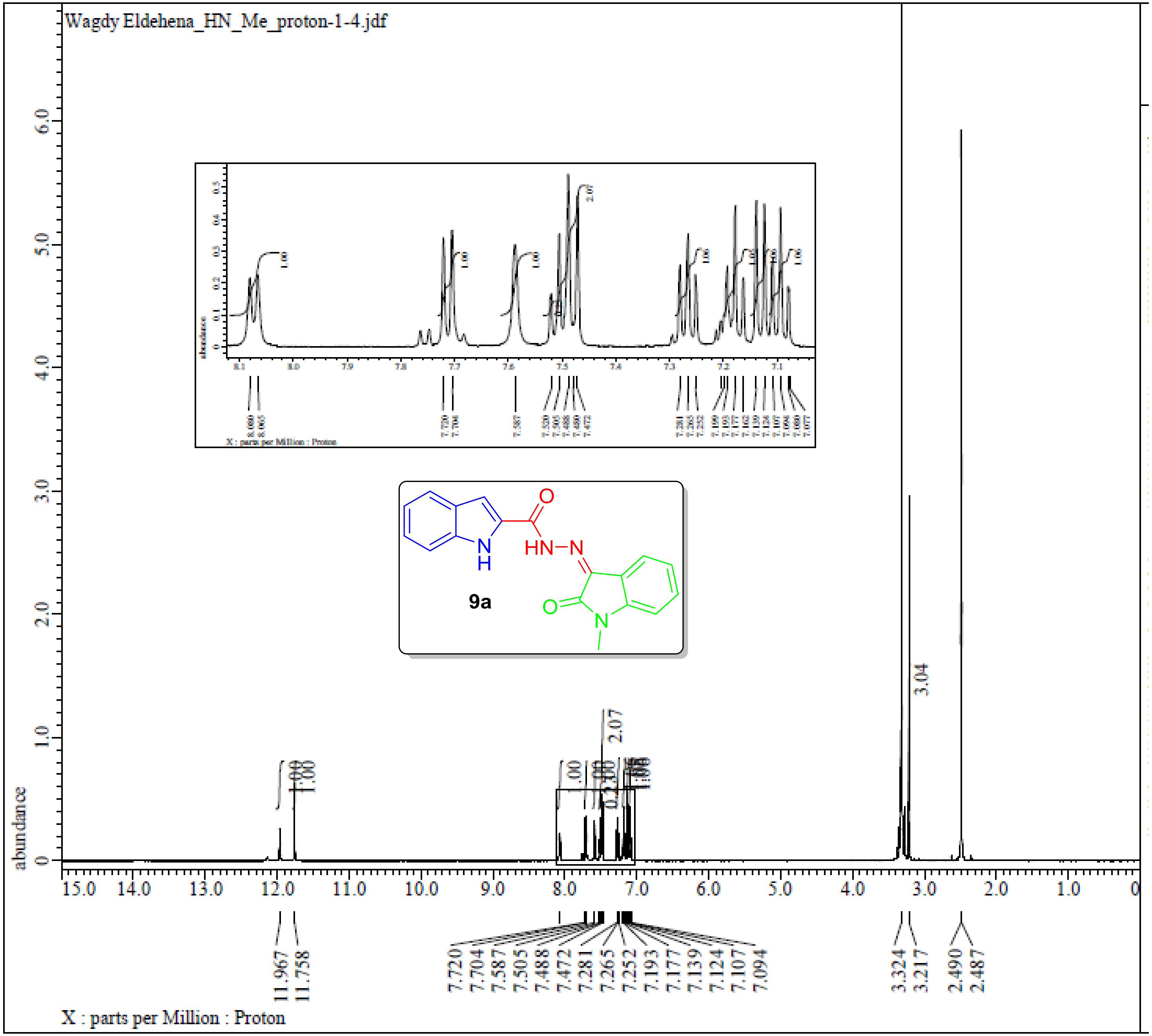
Wagdy Eldehena_HN_Me_proton-1-4.jdf
abundance
X : parts per Million : Proton
9a
HN−N
O
N
H
11.967
11.758
7.720
7.704
7.587
7.505
7.488
7.472
7.281
7.265
7.252
7.193
7.177
7.139
7.124
7.107
7.094
3.324
3.217
2.490
2.487
3.04
2.07

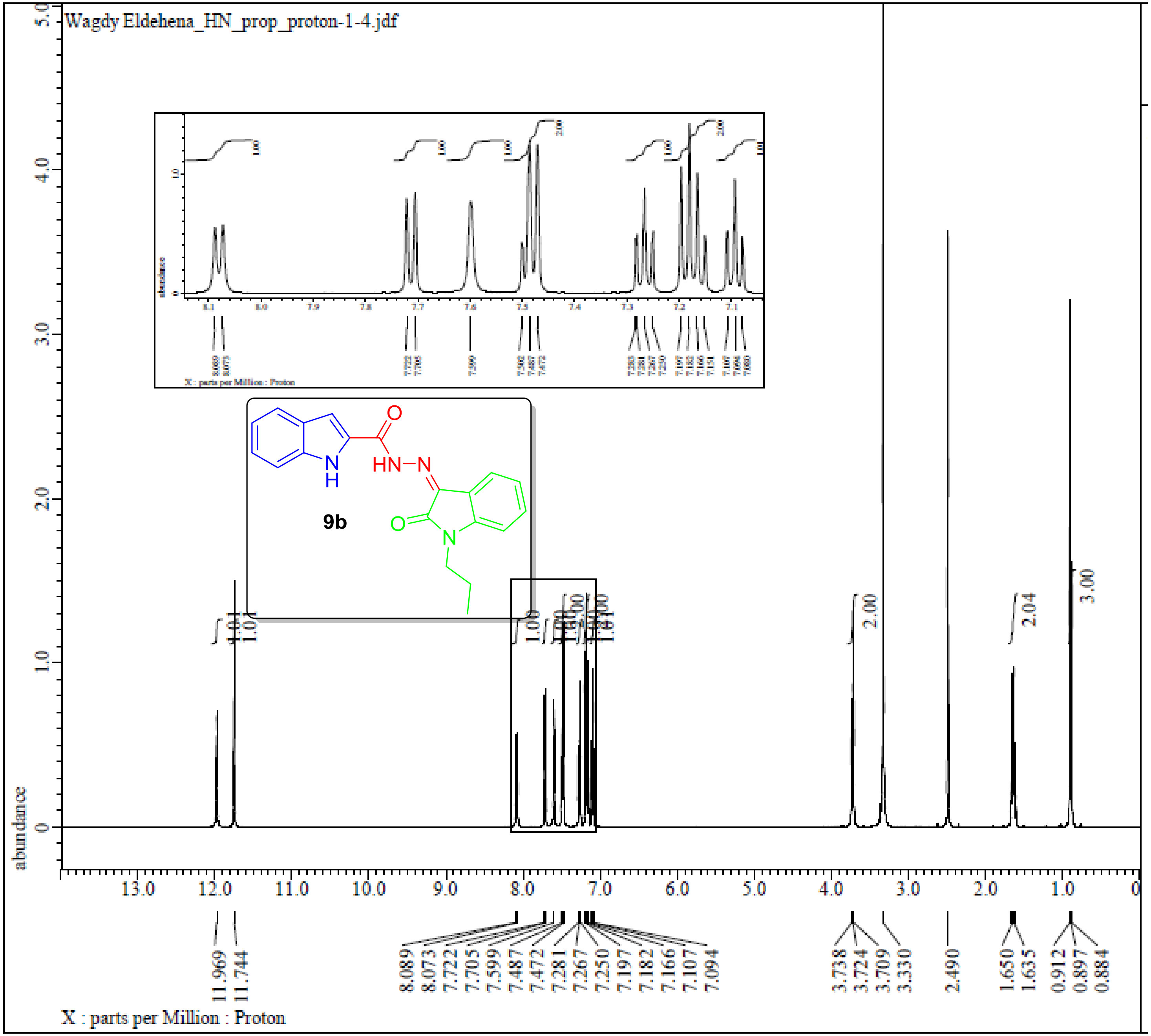
Wagdy Eldehena_HN_prop_proton-1-4.jdf
9b
abundance
X : parts per Million : Proton
11.969
11.744
8.089
8.073
7.722
7.705
7.599
7.487
7.472
7.281
7.267
7.250
7.197
7.182
7.166
7.107
7.094
3.738
3.724
3.709
3.330
2.490
1.650
1.635
0.912
0.897
0.884
2.00
2.04
3.00

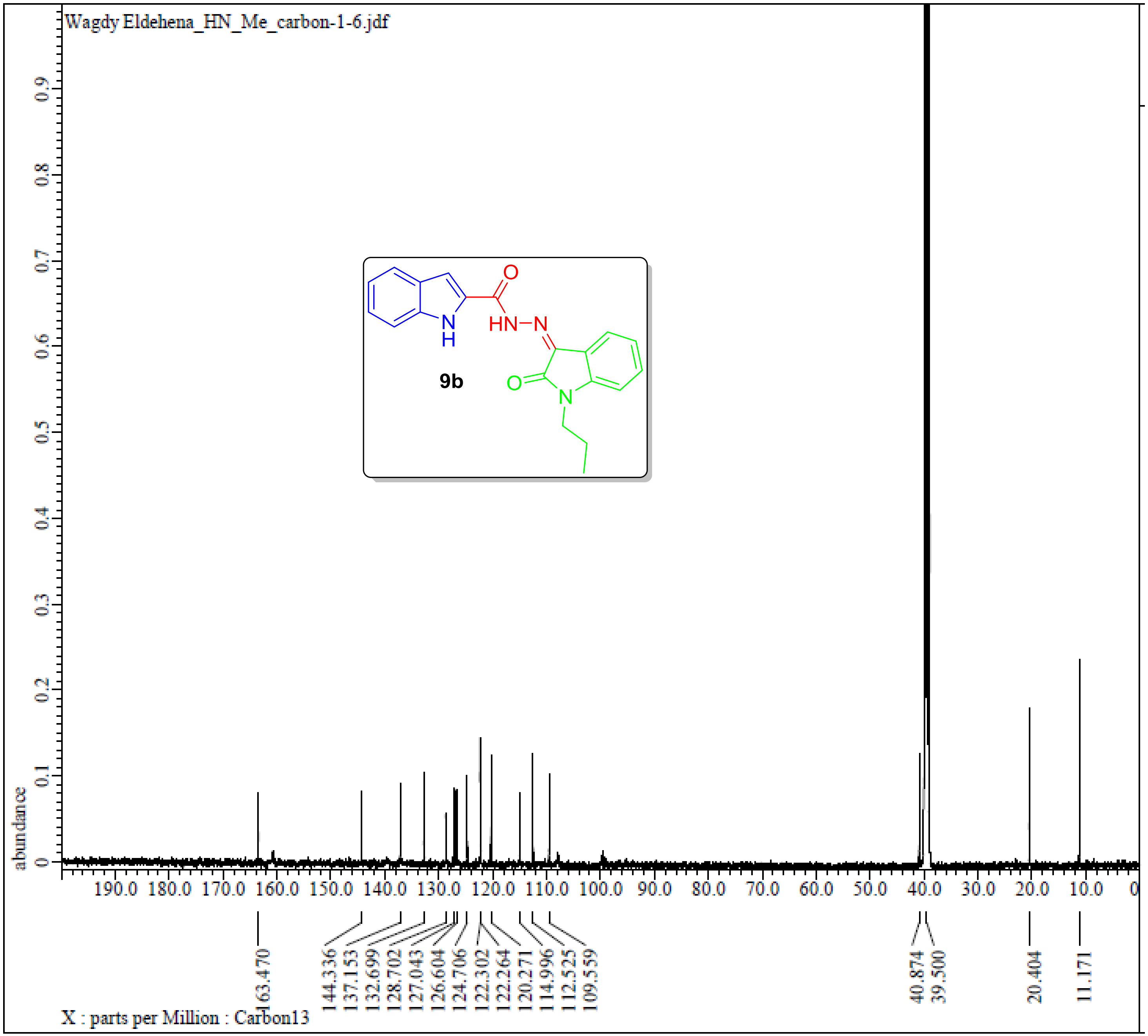
Wagdy Eldehena_HN_Me_carbon-1-6.jdf
9b
O
HN
N
H
abundance
0.9
0.8
0.7
0.6
0.5
0.4
0.3
0.2
0.1
0
190.0 180.0 170.0 160.0 150.0 140.0 130.0 120.0 110.0 100.0 90.0 80.0 70.0 60.0 50.0 40.0 30.0 20.0 10.0 0
163.470
144.336
137.153
132.699
128.702
127.043
126.604
124.706
122.302
122.264
120.271
114.996
112.525
109.559
40.874
39.500
20.404
11.171
X : parts per Million : Carbon13

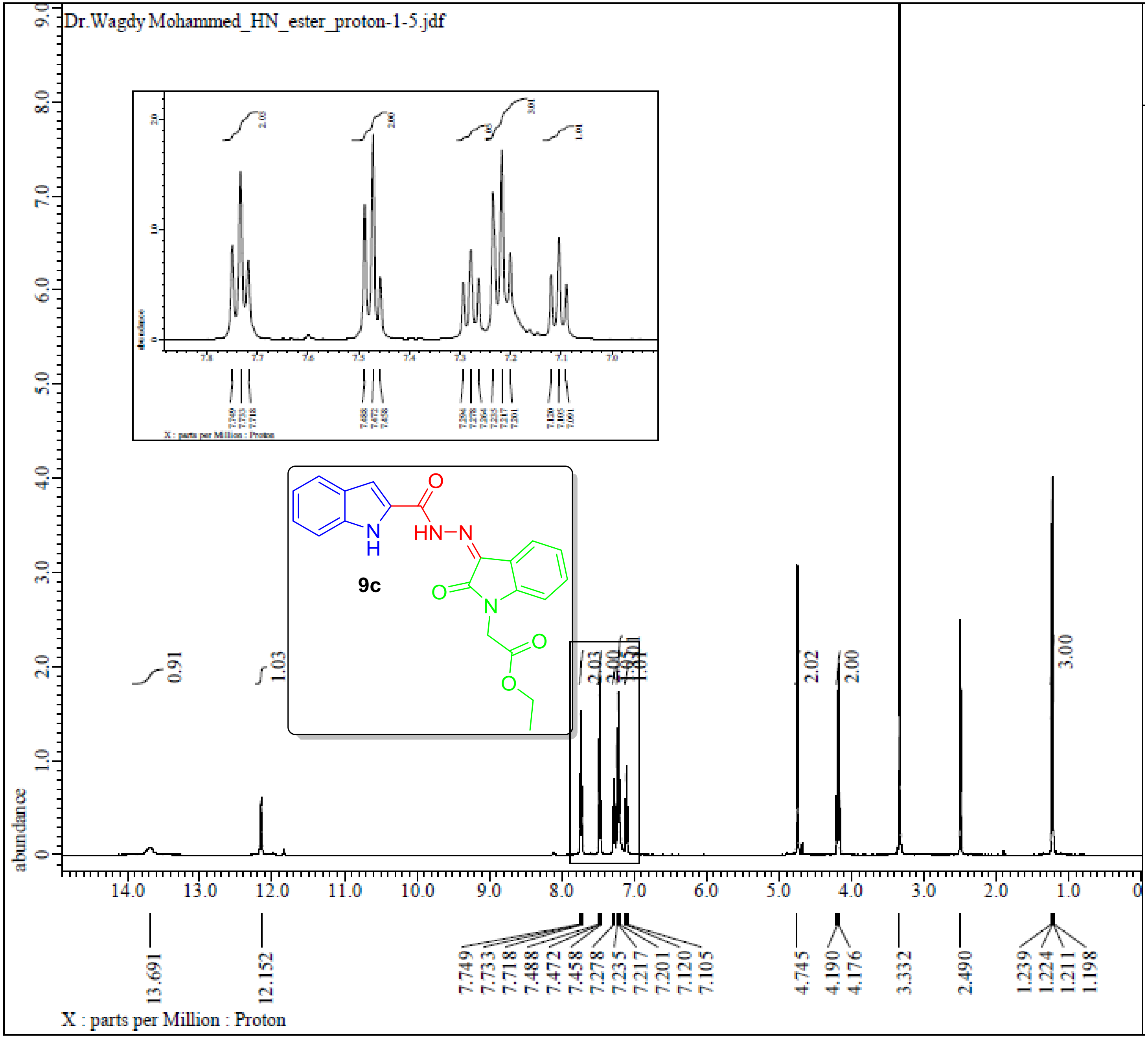
Dr.Wagdy Mohammed_HN_ester_proton-1-5.jdf
abundance
X : parts per Million : Proton
9c
HN−N
N
H
13.691
12.152
7.749
7.733
7.718
7.488
7.472
7.458
7.278
7.235
7.217
7.201
7.120
7.105
4.745
4.190
4.176
3.332
2.490
1.239
1.224
1.211
1.198
0.91
1.03
2.03
2.02
2.00
3.00

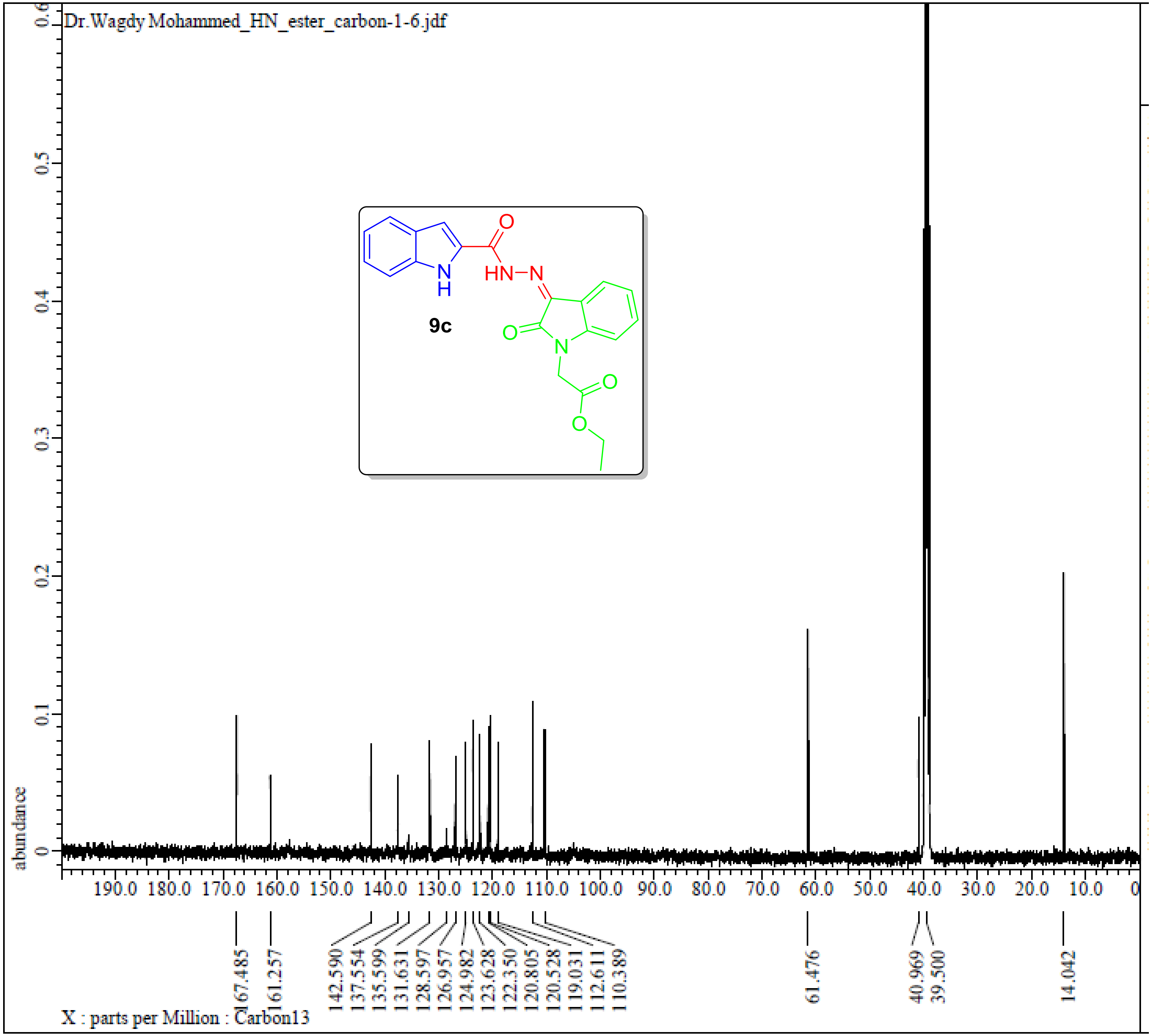
Dr.Wagdy Mohammed_HN_ester_carbon-1-6.jdf
9c
O
HN—N
N
H
O
N
O
O
abundance
0
0.1
0.2
0.3
0.4
0.5
0.6
190.0 180.0 170.0 160.0 150.0 140.0 130.0 120.0 110.0 100.0 90.0 80.0 70.0 60.0 50.0 40.0 30.0 20.0 10.0 0
167.485
161.257
142.590
137.554
135.599
131.631
128.597
126.957
124.982
123.628
122.350
120.805
120.528
119.031
112.611
110.389
61.476
40.969
39.500
14.042
X : parts per Million : Carbon13

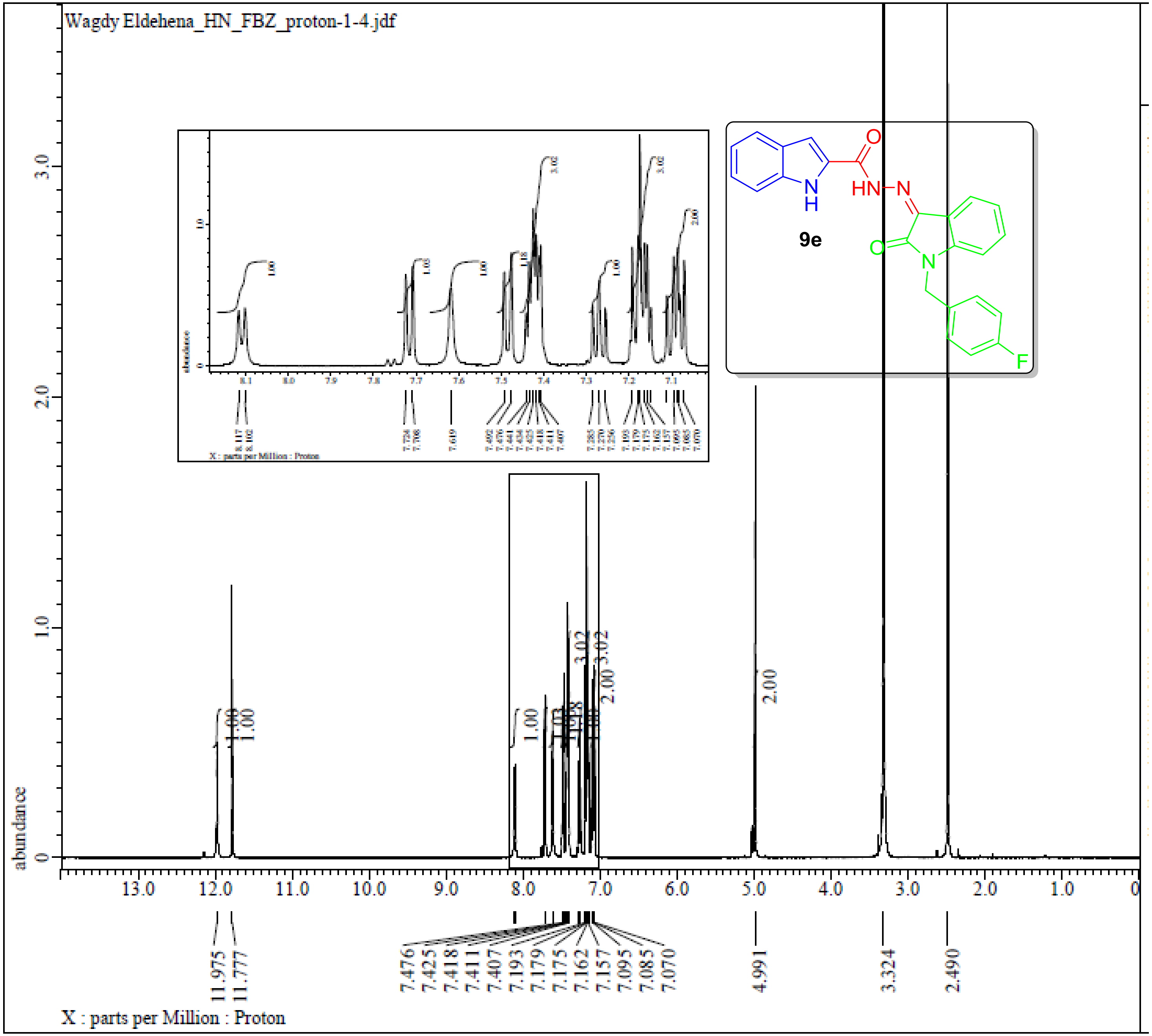
Wagdy Eldehena_HN_FBZ_proton-1-4.jdf
9e
abundance
X : parts per Million : Proton
11.975
11.777
7.476
7.425
7.418
7.411
7.407
7.193
7.179
7.175
7.162
7.157
7.095
7.085
7.070
4.991
3.324
2.490
1.00
1.00
2.00

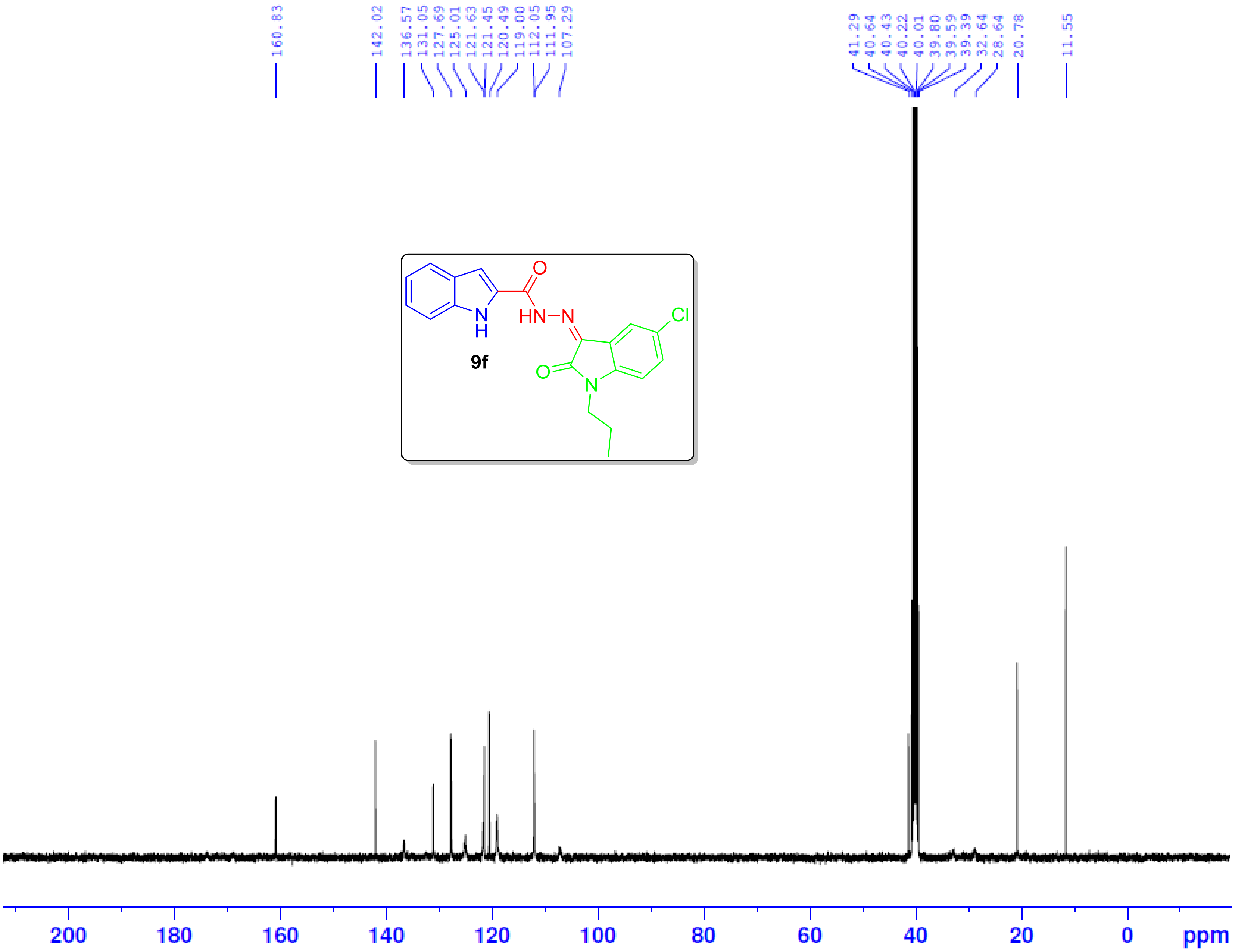
160.83
142.02
136.57
131.05
127.69
125.01
121.63
121.45
120.49
119.00
112.05
111.95
107.29
41.29
40.64
40.43
40.22
40.01
39.80
39.59
39.39
32.64
28.64
20.78
11.55
O
HN
N
H
N
Cl
O
N
9f
200
180
160
140
120
100
80
60
40
20
0
ppm

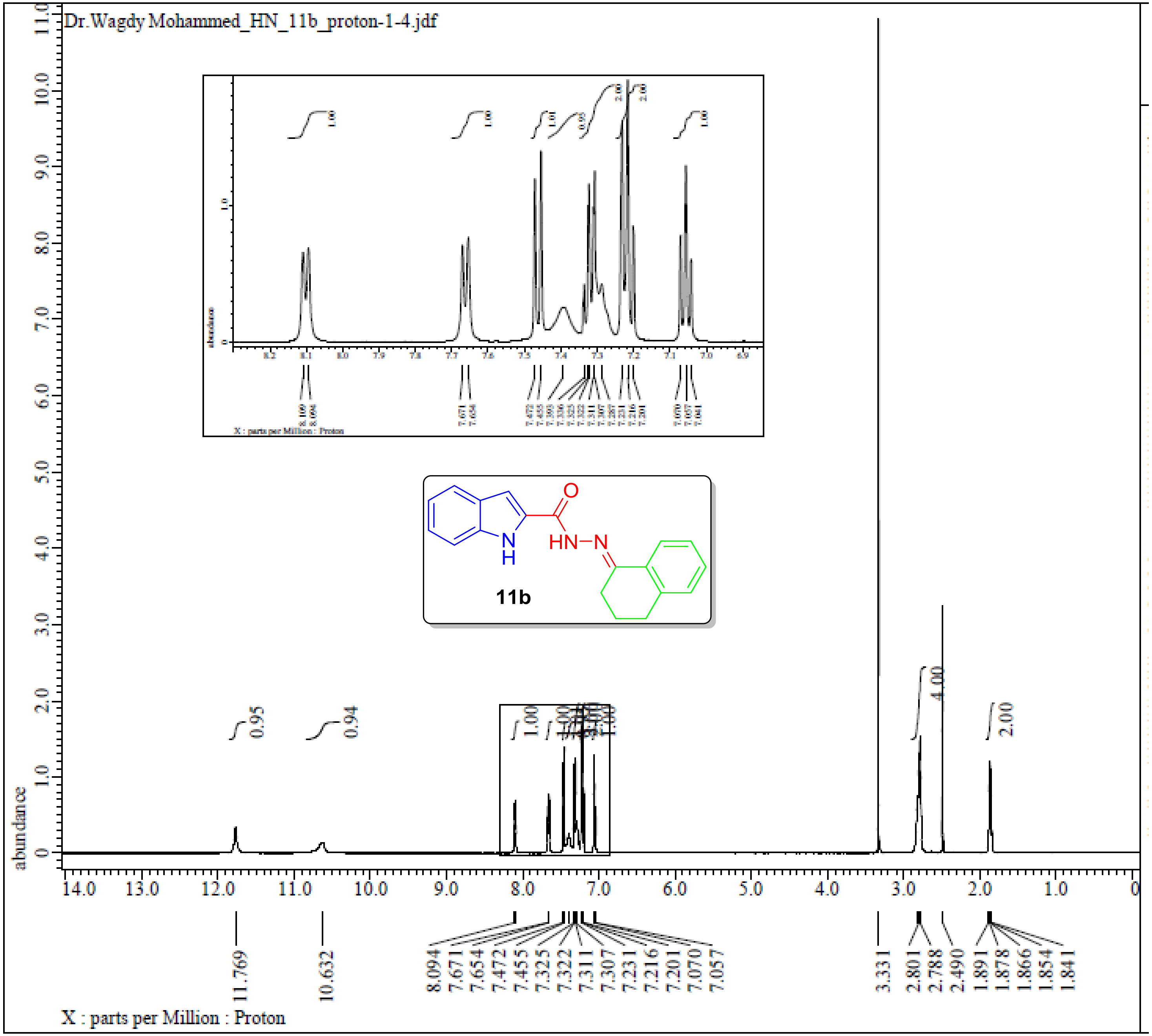
Dr.Wagdy Mohammed_HN_11b_proton-1-4.jdf
11b
abundance
X : parts per Million : Proton
11.769
10.632
8.094
7.671
7.654
7.472
7.455
7.325
7.322
7.311
7.307
7.231
7.216
7.201
7.070
7.057
3.331
2.801
2.788
2.490
1.891
1.878
1.866
1.854
1.841
0.95
0.94
1.00
4.00
2.00

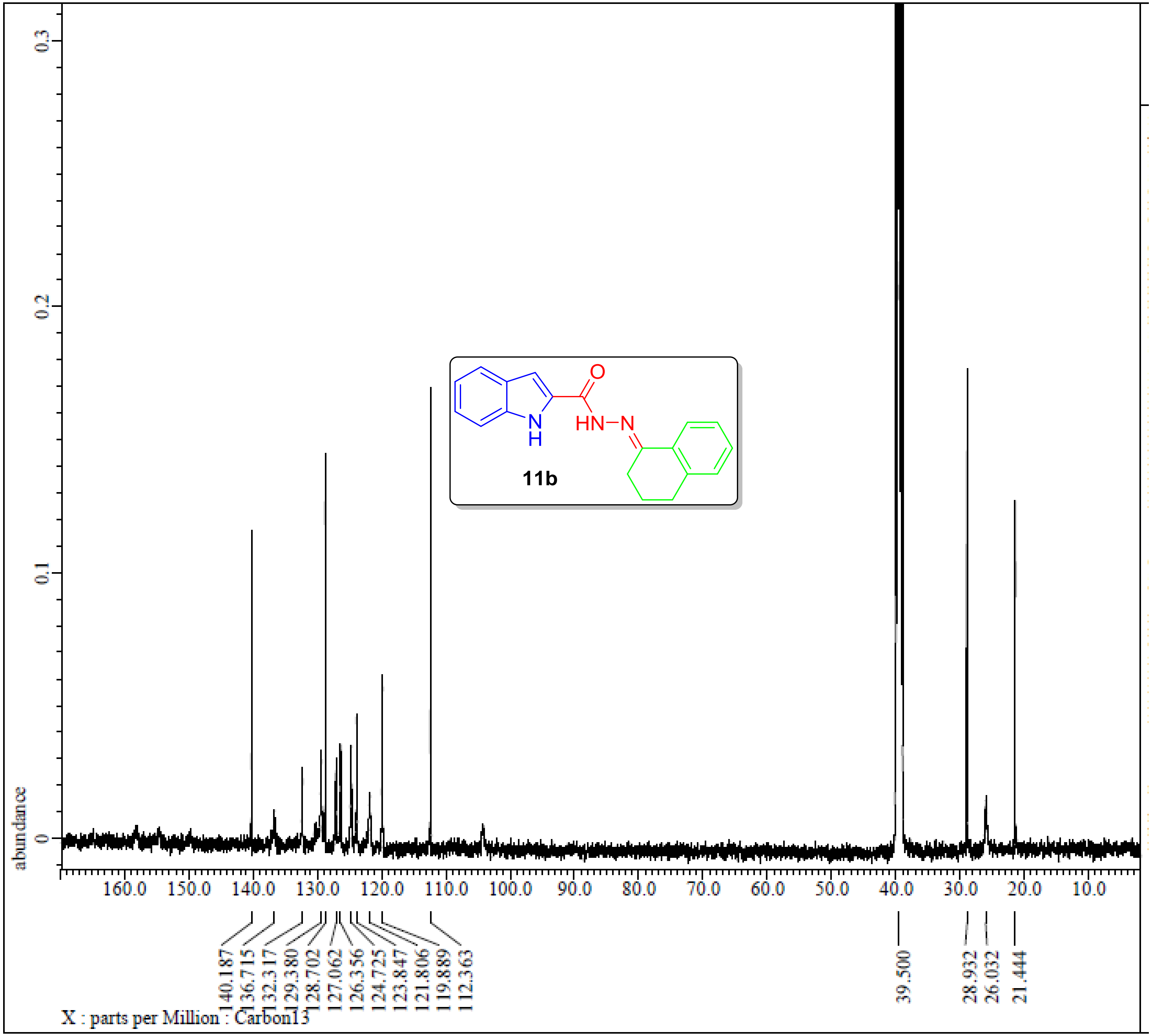

0.3
0.2
0.1
0
abundance
O
HN
N
N
H
11b
160.0
150.0
140.0
130.0
120.0
110.0
100.0
90.0
80.0
70.0
60.0
50.0
40.0
30.0
20.0
10.0
140.187
136.715
132.317
129.380
128.702
127.062
126.356
124.725
123.847
121.806
119.889
112.363
39.500
28.932
26.032
21.444
X : parts per Million : Carbon13

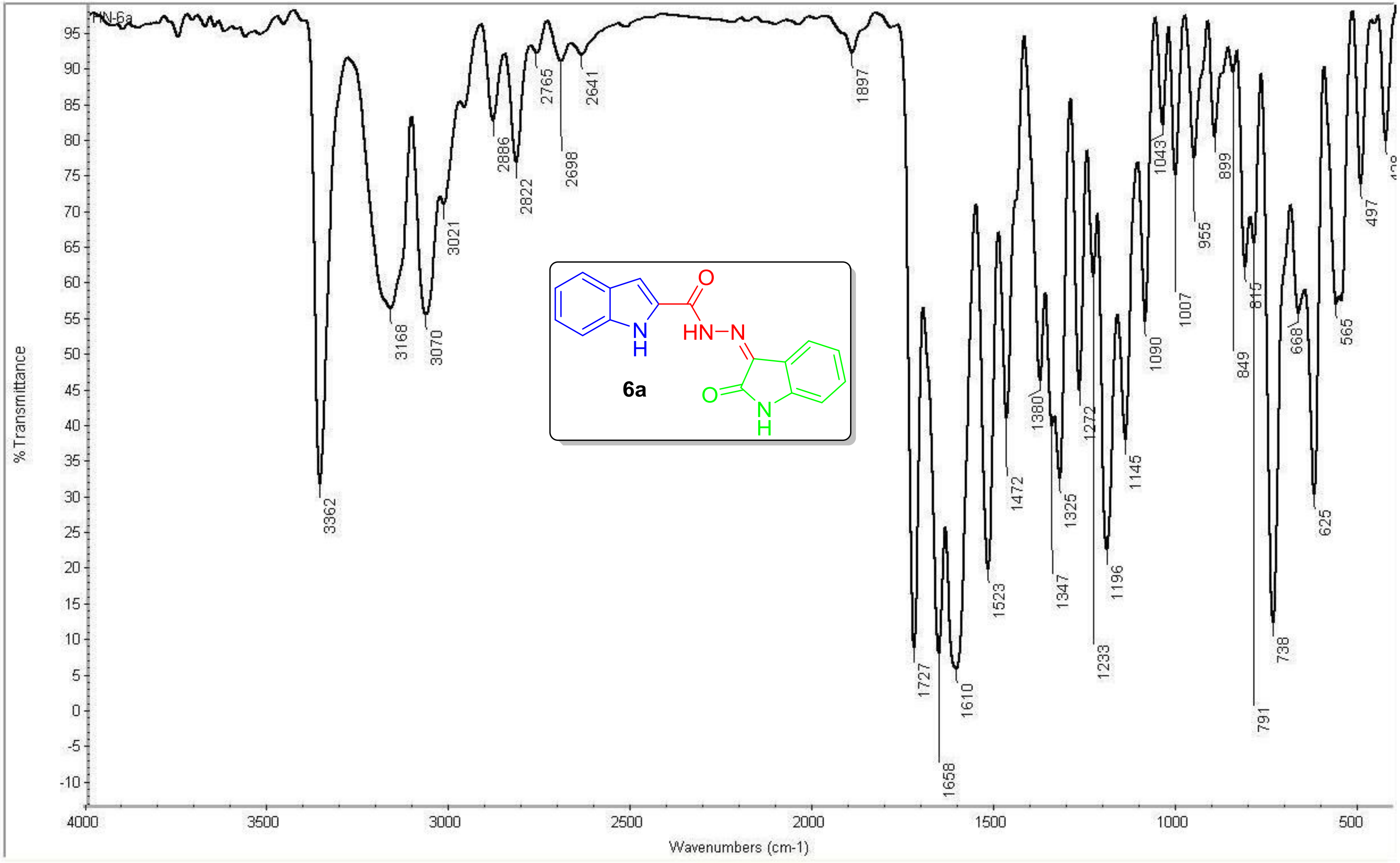
HN-6a
% Transmittance
Wavenumbers (cm-1)
6a
3362
3168
3070
3021
2886
2822
2765
2698
2641
1897
1727
1658
1610
1523
1472
1380
1347
1325
1272
1233
1196
1145
1090
1043
1007
955
899
849
815
791
738
668
625
565
497

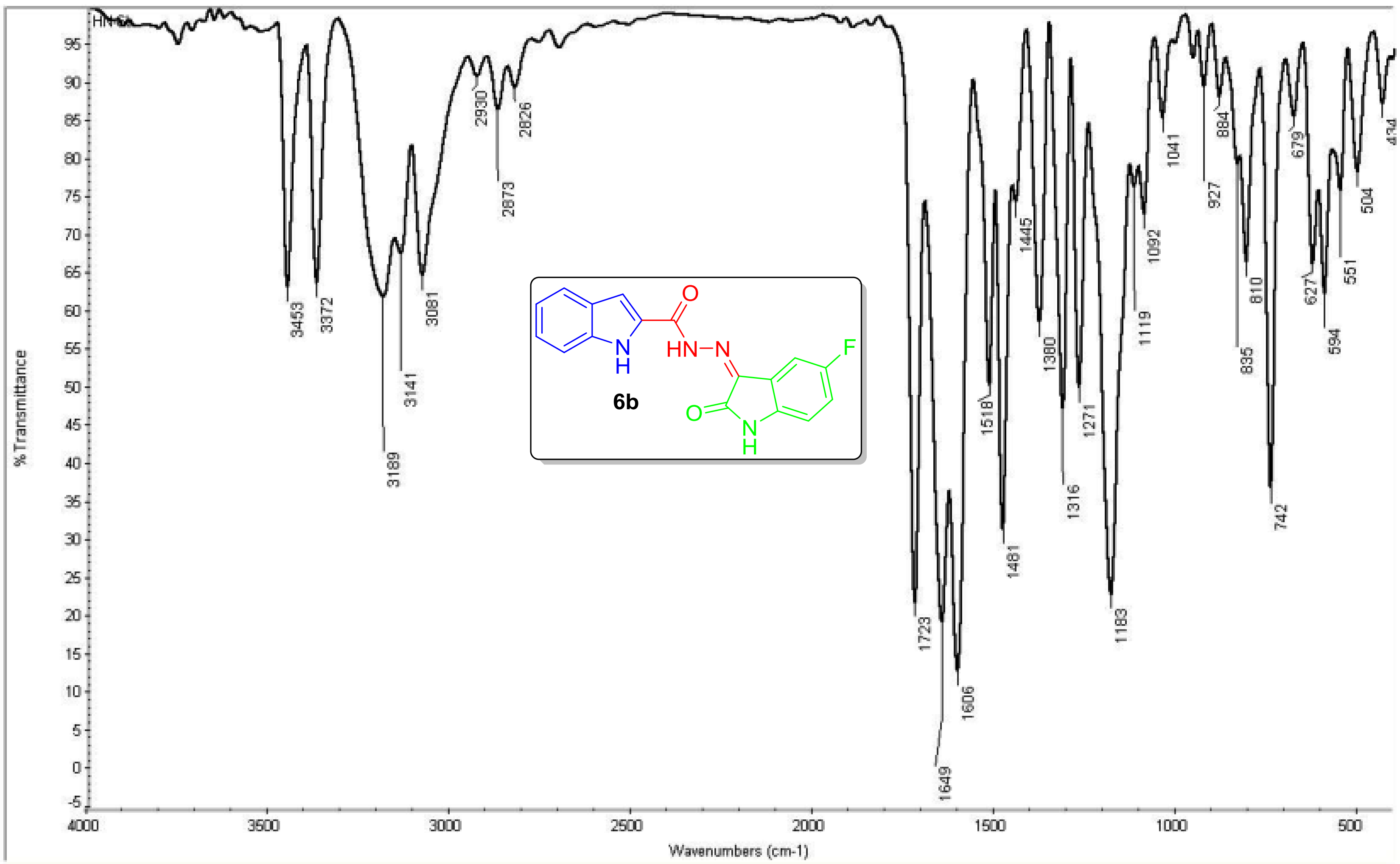

% Transmittance
Wavenumbers (cm-1)
6b
3453
3372
3189
3141
3081
2930
2873
2826
1723
1649
1606
1518
1481
1445
1380
1316
1271
1183
1119
1092
1041
927
884
835
810
742
679
627
594
551
504

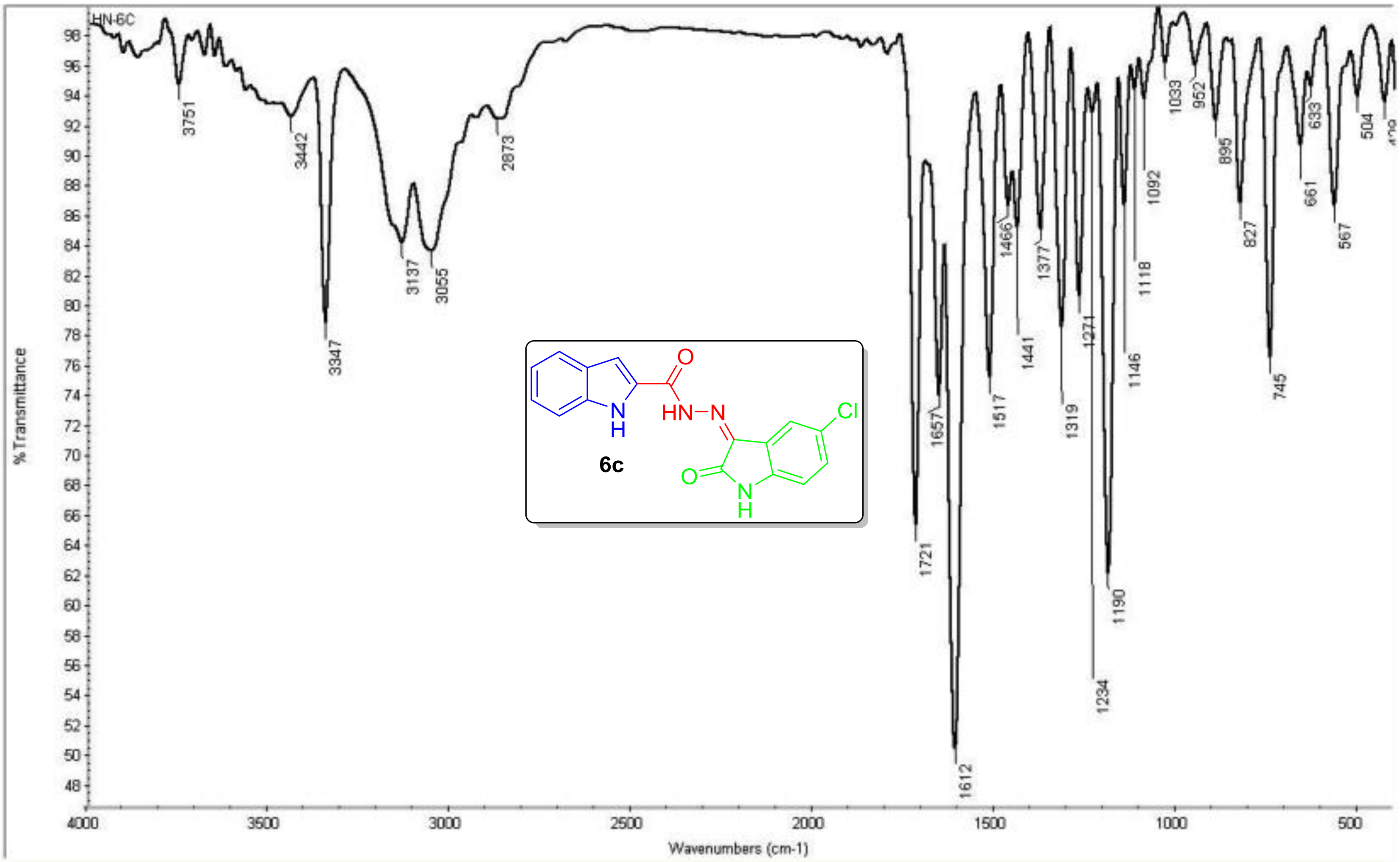

HN-6C
% Transmittance
Wavenumbers (cm-1)
4000
3500
3000
2500
2000
1500
1000
500
3751
3442
3347
3137
3055
2873
1721
1657
1612
1517
1466
1441
1377
1319
1271
1234
1190
1146
1118
1092
1033
952
895
827
745
661
633
567
504
O
HN–N
N
H
Cl
O
N
H
6c

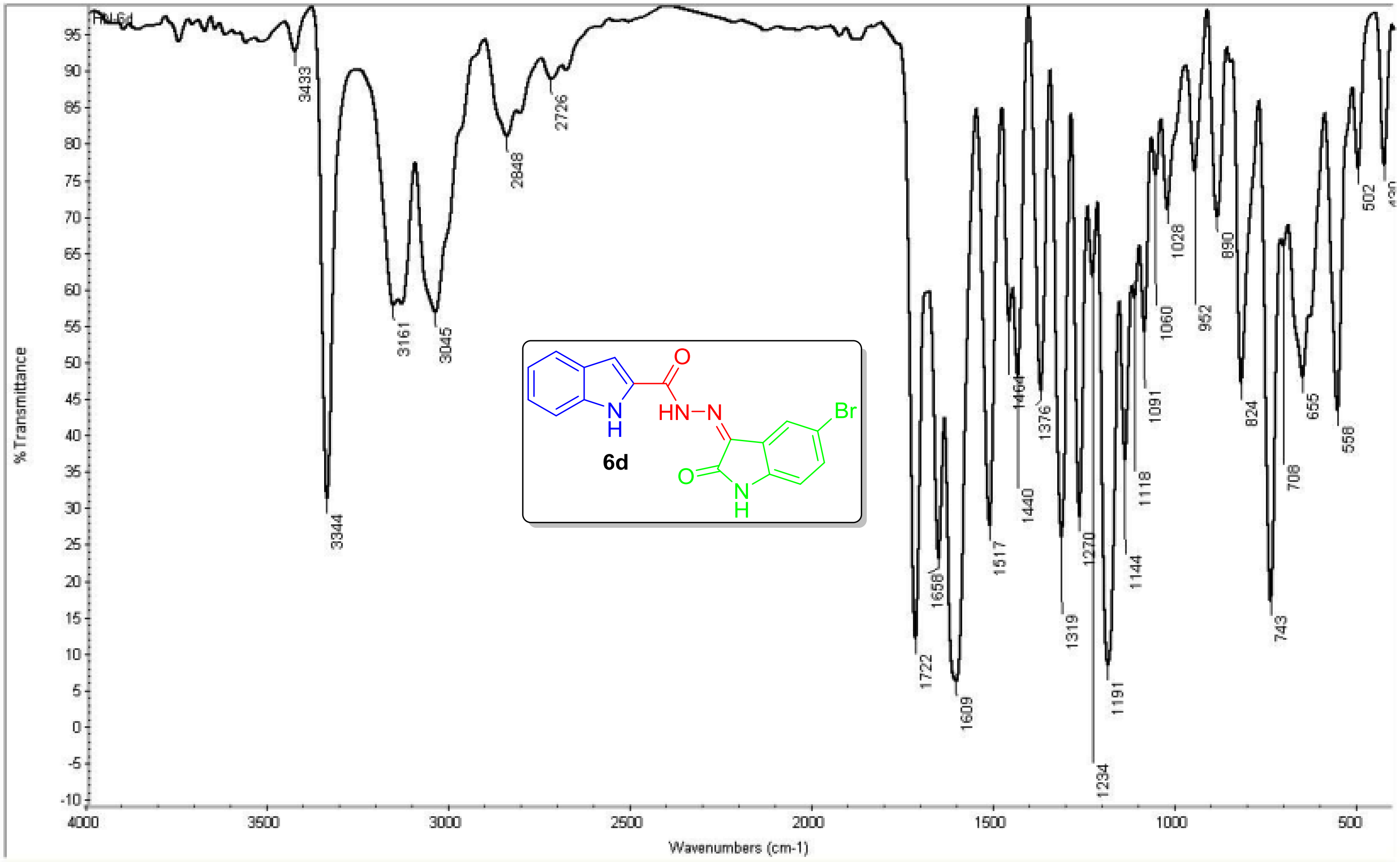

% Transmittance
Wavenumbers (cm-1)
4000
3500
3000
2500
2000
1500
1000
500
95
90
85
80
75
70
65
60
55
50
45
40
35
30
25
20
15
10
5
0
-5
-10
3433
3344
3161
3045
2848
2726
1722
1658
1609
1517
1464
1440
1376
1319
1270
1234
1191
1144
1118
1091
1060
1028
952
890
824
743
708
655
558
502
O
HN–N
N
H
Br
O
N
H
6d

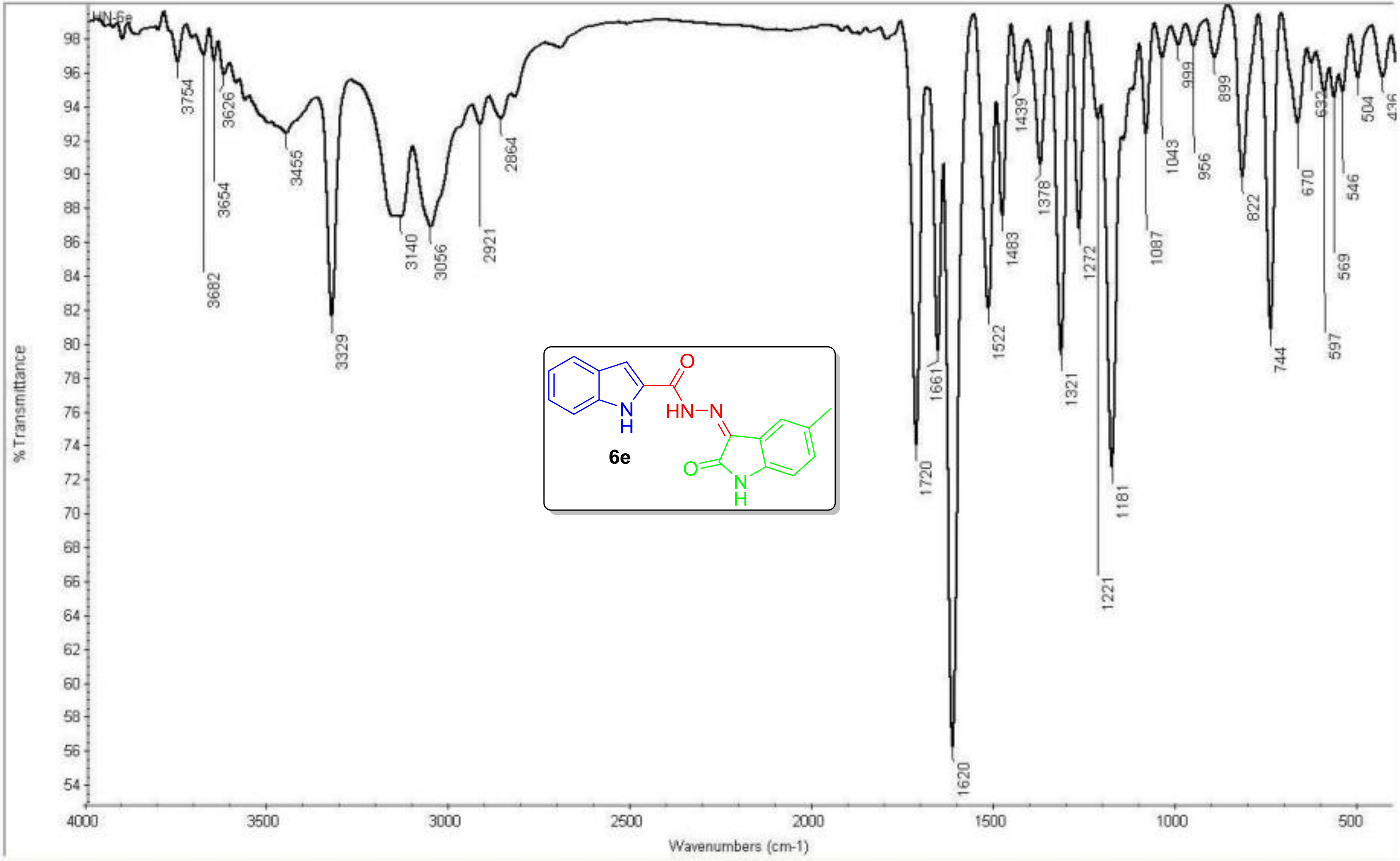

HN-6e
% Transmittance
Wavenumbers (cm-1)
3754
3682
3654
3626
3455
3329
3140
3056
2921
2864
1720
1661
1620
1522
1483
1439
1378
1321
1272
1221
1181
1087
1043
999
956
899
822
744
670
632
597
569
546
504
436
6e
O
HN
N
H

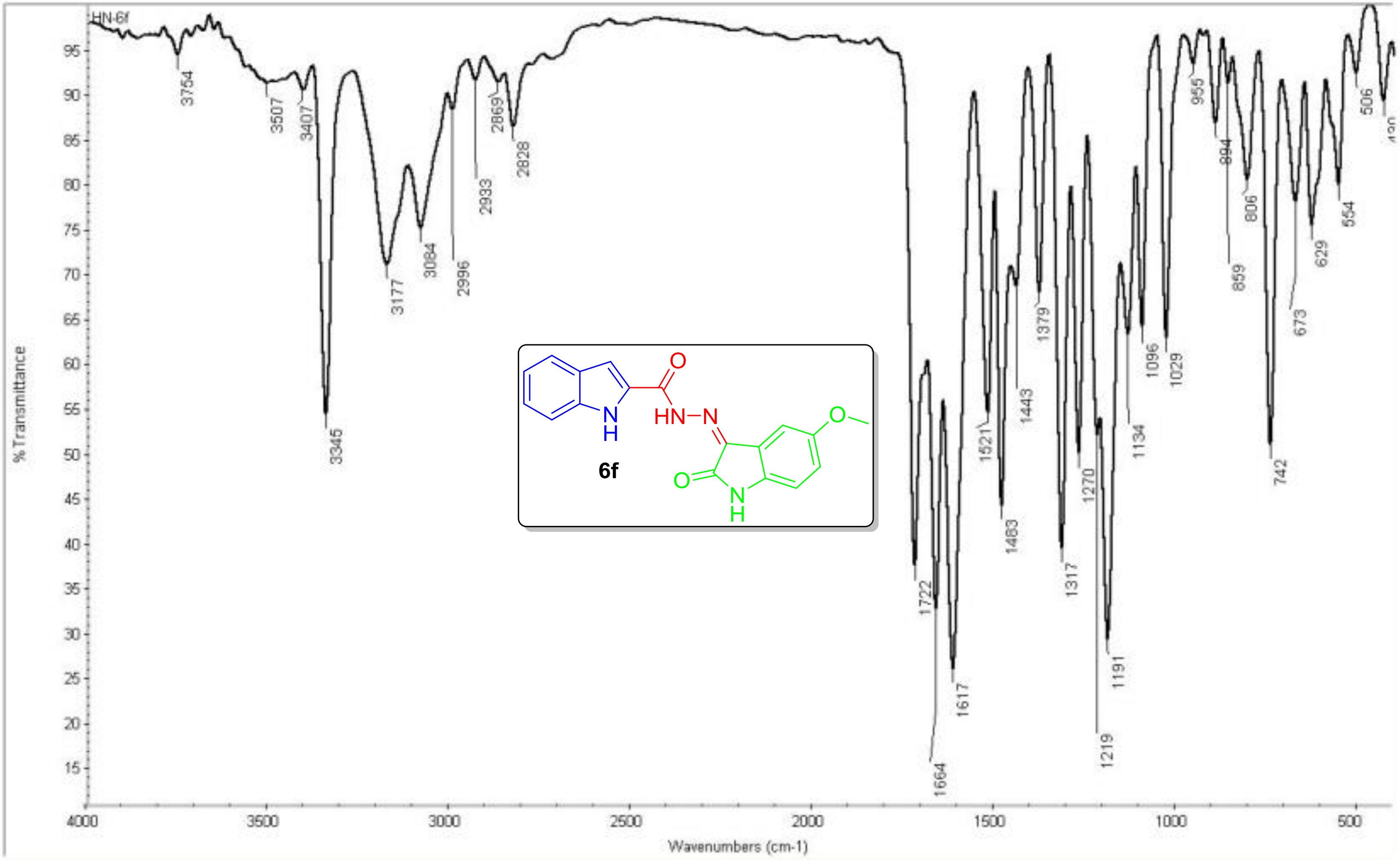
HN-6f
% Transmittance
Wavenumbers (cm-1)
4000
3500
3000
2500
2000
1500
1000
500
95
90
85
80
75
70
65
60
55
50
45
40
35
30
25
20
15
3754
3507
3407
3345
3177
3084
2996
2933
2869
2828
1722
1664
1617
1521
1483
1443
1379
1317
1270
1219
1191
1134
1096
1029
955
894
859
806
742
673
629
554
506
6f

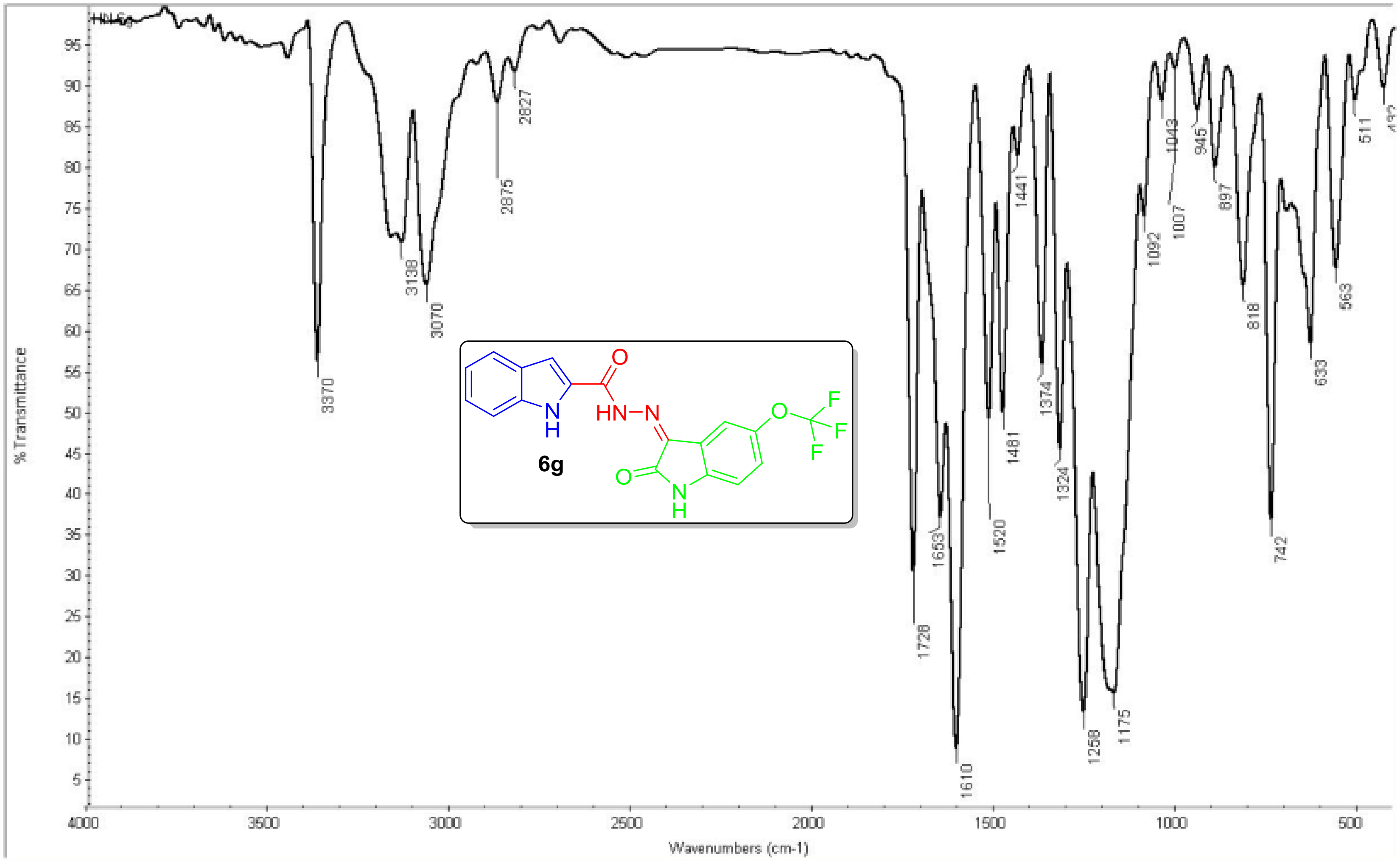
%Transmittance
95
90
85
80
75
70
65
60
55
50
45
40
35
30
25
20
15
10
5
4000
3500
3000
2500
2000
1500
1000
500
Wavenumbers (cm-1)
3370
3138
3070
2875
2827
1728
1653
1610
1520
1481
1441
1374
1324
1258
1175
1092
1043
1007
945
897
818
742
633
563
511
O
HN
N
H
N
6g
O
N
H
O
F
F
F

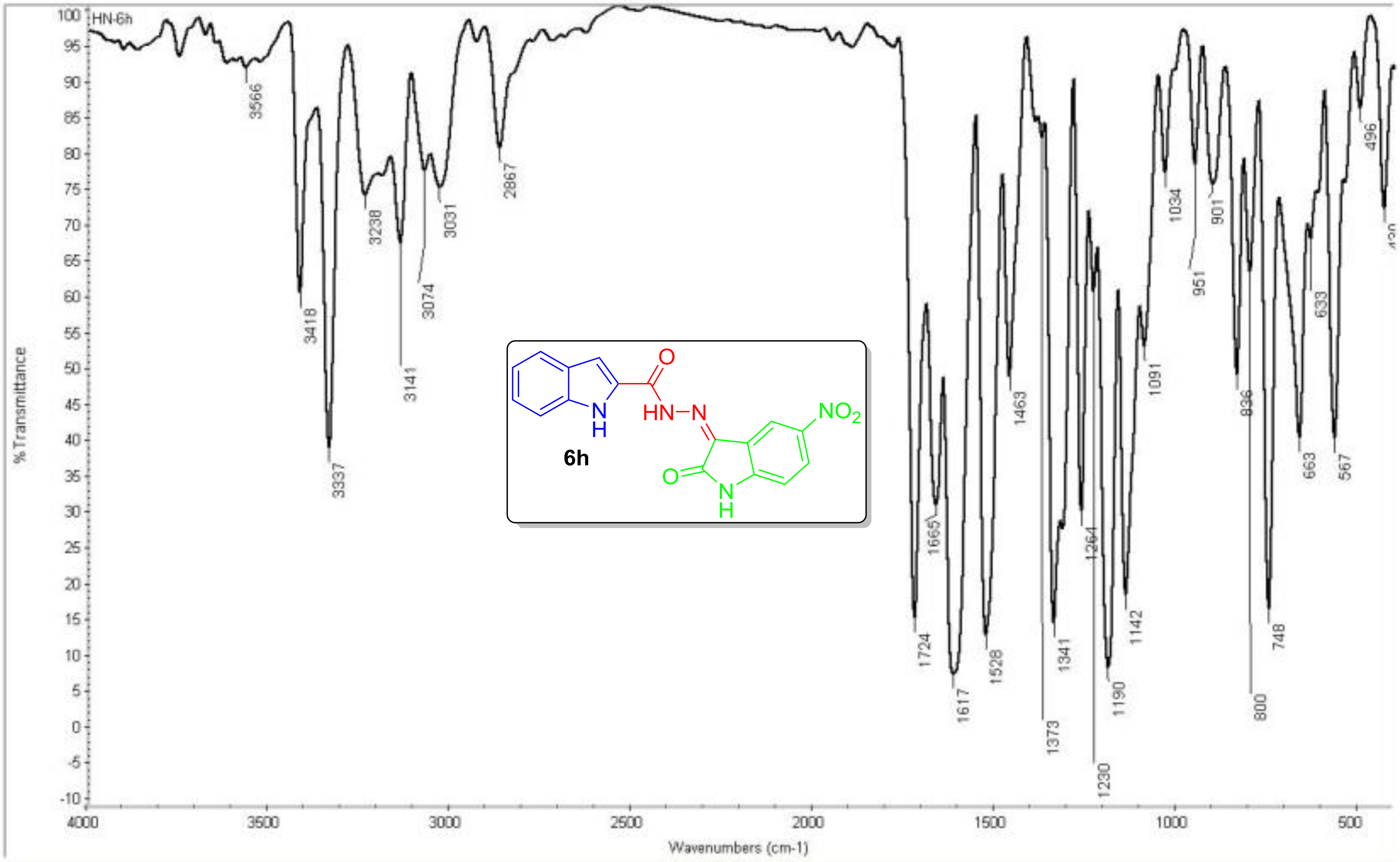
HN-6h
% Transmittance
Wavenumbers (cm-1)
3566
3418
3337
3238
3141
3074
3031
2867
1724
1665
1617
1528
1463
1373
1341
1264
1230
1190
1142
1091
1034
951
901
836
800
748
663
633
567
496
6h
O
HN–N
NO2
O
N
H
N
H

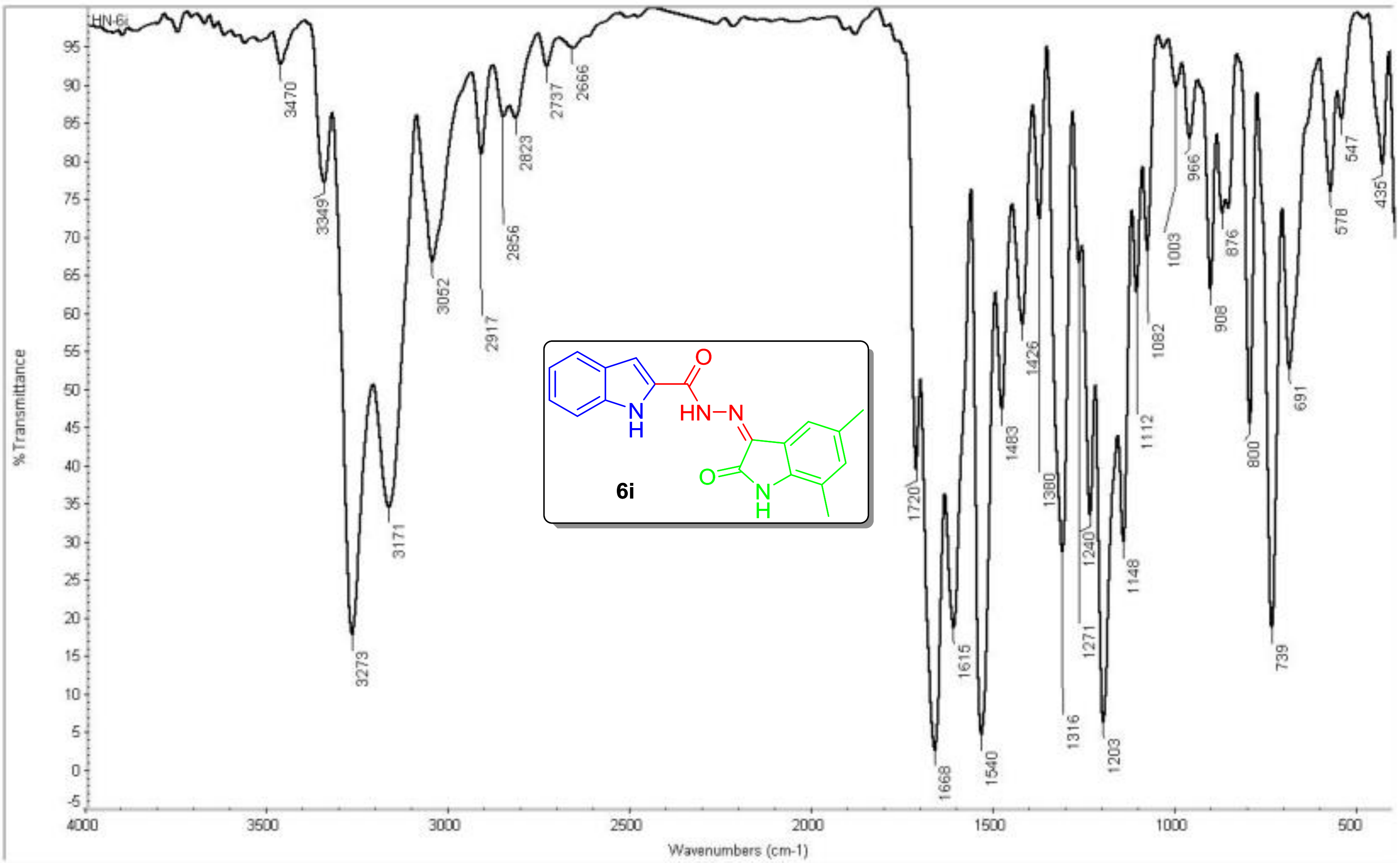
HN-6i
% Transmittance
Wavenumbers (cm-1)
3470
3349
3273
3171
3052
2917
2856
2823
2737
2666
1720
1668
1615
1540
1483
1426
1380
1316
1271
1240
1203
1148
1112
1082
1003
966
908
876
800
739
691
578
547
435
6i

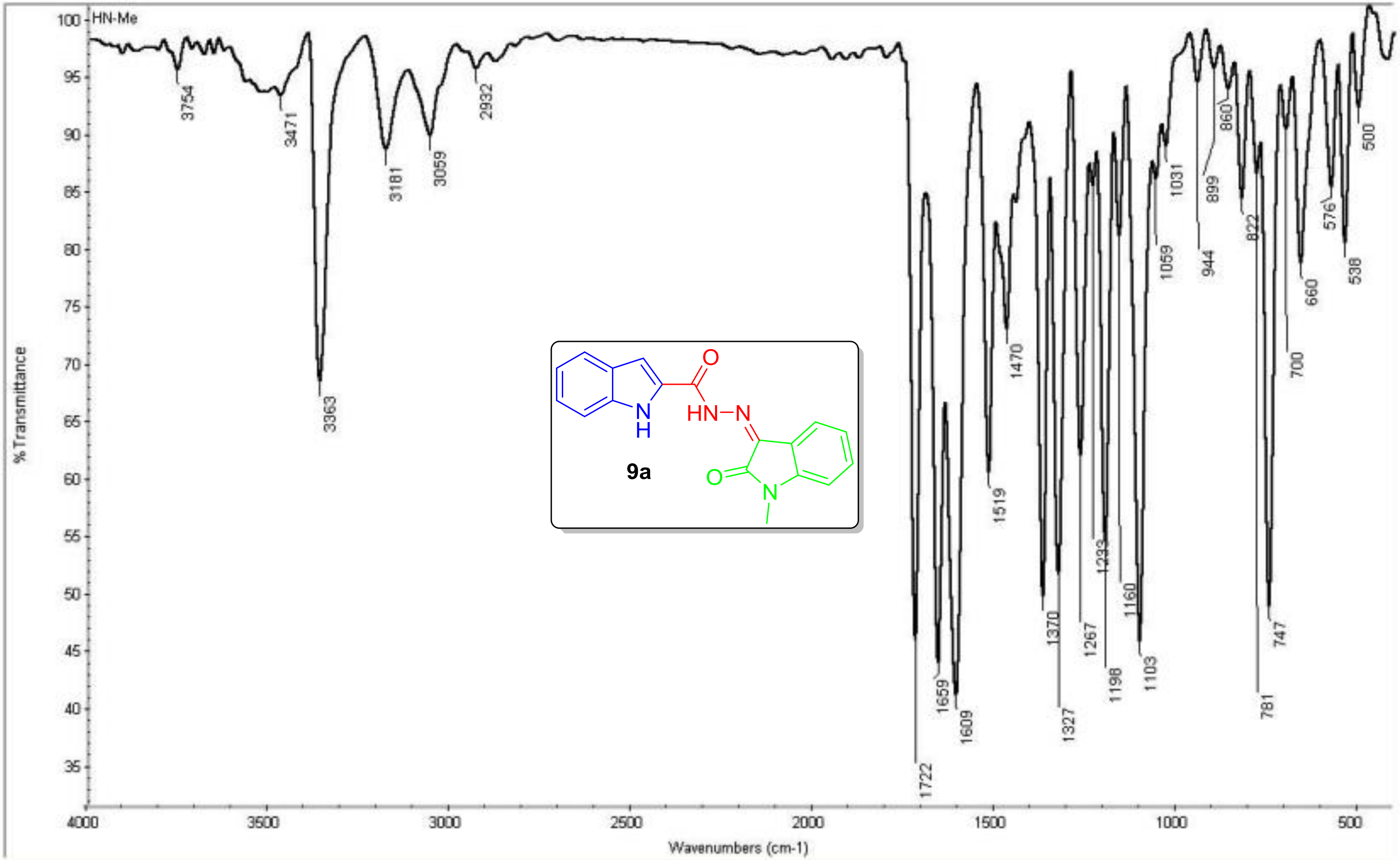
HN-Me
% Transmittance
Wavenumbers (cm-1)
100
95
90
85
80
75
70
65
60
55
50
45
40
35
4000
3500
3000
2500
2000
1500
1000
500
3754
3471
3363
3181
3059
2932
1722
1659
1609
1519
1470
1370
1327
1267
1233
1198
1160
1103
1059
1031
944
899
860
822
781
747
700
660
576
538
500
O
HN–N
N
H
O
N
9a

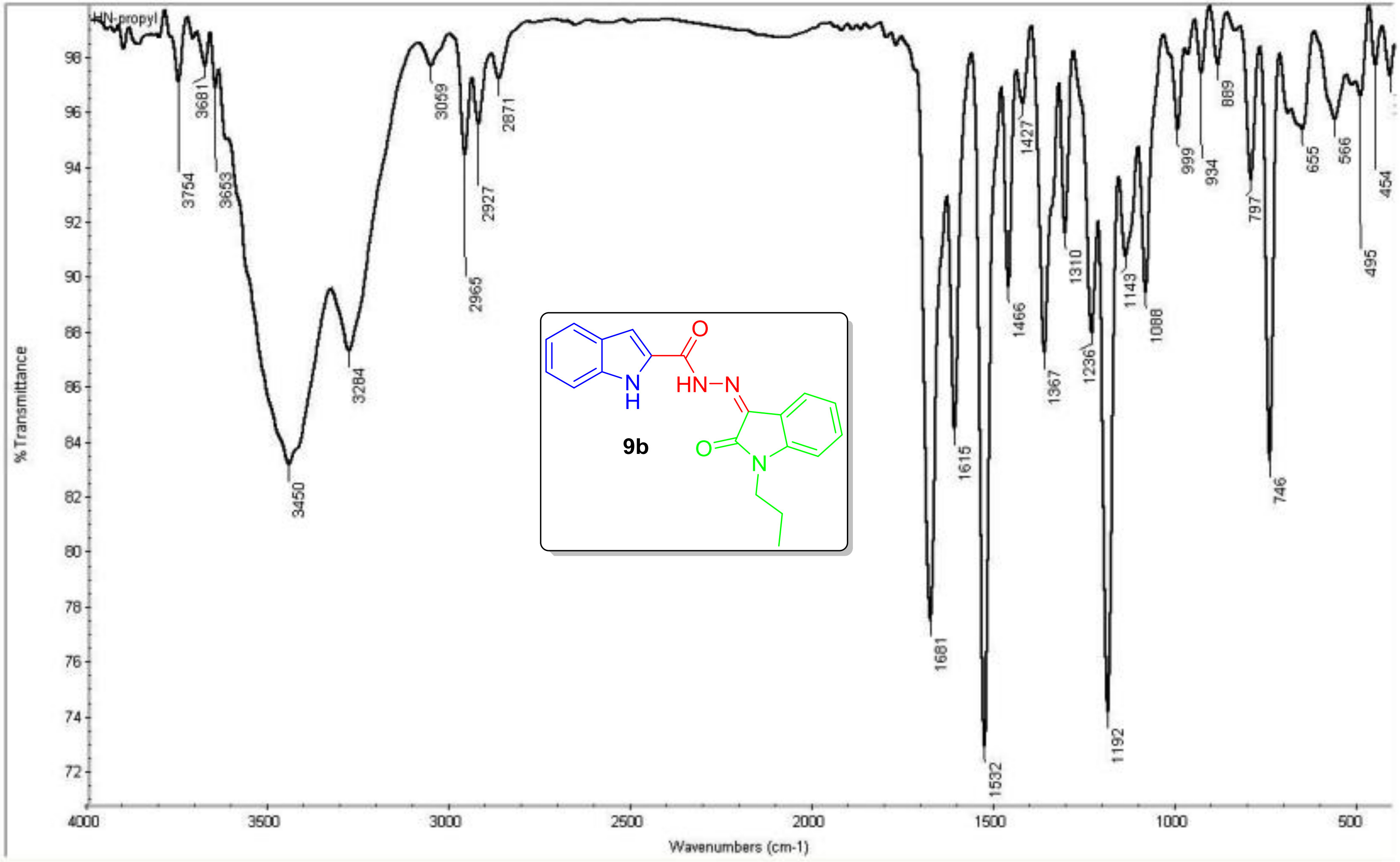
HN-propyl
%Transmittance
Wavenumbers (cm-1)
3754
3681
3653
3450
3284
3059
2965
2927
2871
1681
1615
1532
1466
1427
1367
1310
1236
1192
1143
1088
999
934
889
797
746
655
566
495
454
9b

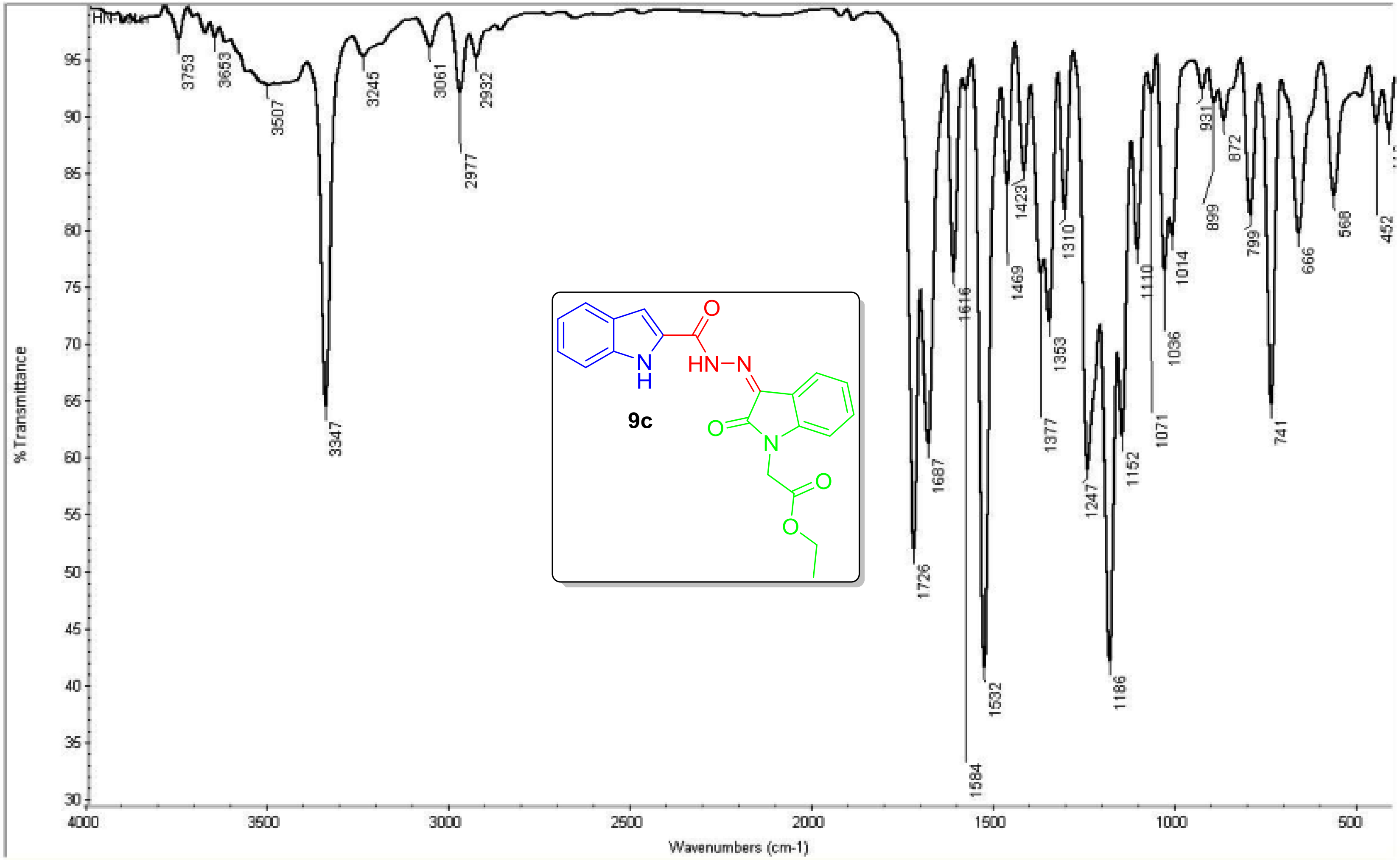

9c
% Transmittance
Wavenumbers (cm-1)
4000
3500
3000
2500
2000
1500
1000
500
95
90
85
80
75
70
65
60
55
50
45
40
35
30
3753
3653
3507
3347
3245
3061
2977
2932
1726
1687
1616
1584
1532
1469
1423
1377
1353
1310
1247
1186
1152
1110
1071
1036
1014
931
899
872
799
741
666
568
452

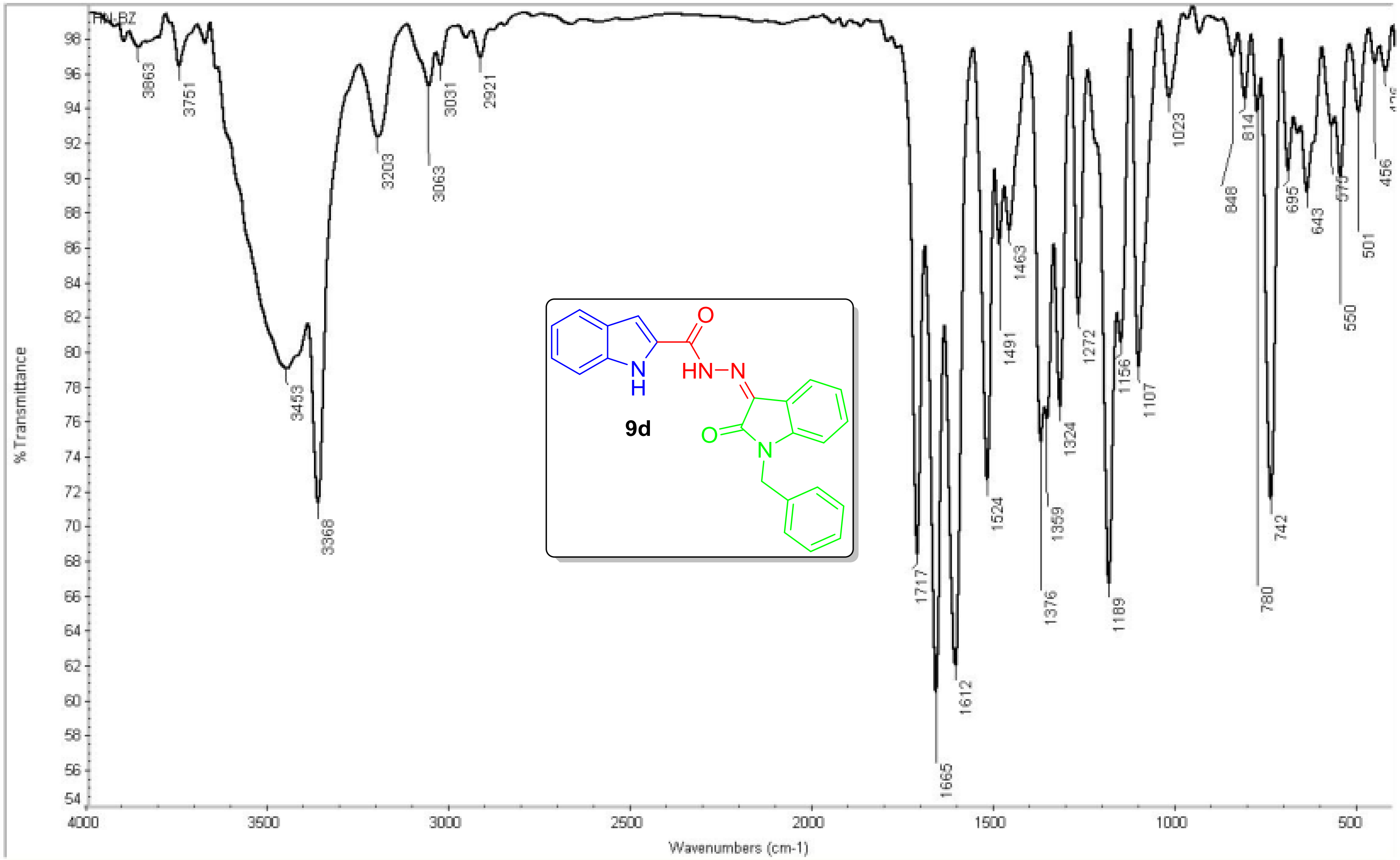
% Transmittance
Wavenumbers (cm-1)
98
96
94
92
90
88
86
84
82
80
78
76
74
72
70
68
66
64
62
60
58
56
54
4000
3500
3000
2500
2000
1500
1000
500
3863
3751
3453
3368
3203
3063
3031
2921
1717
1665
1612
1524
1491
1463
1376
1359
1324
1272
1189
1156
1107
1023
848
814
780
742
695
643
550
501
456
9d
O
HN–N
N
H
O
N

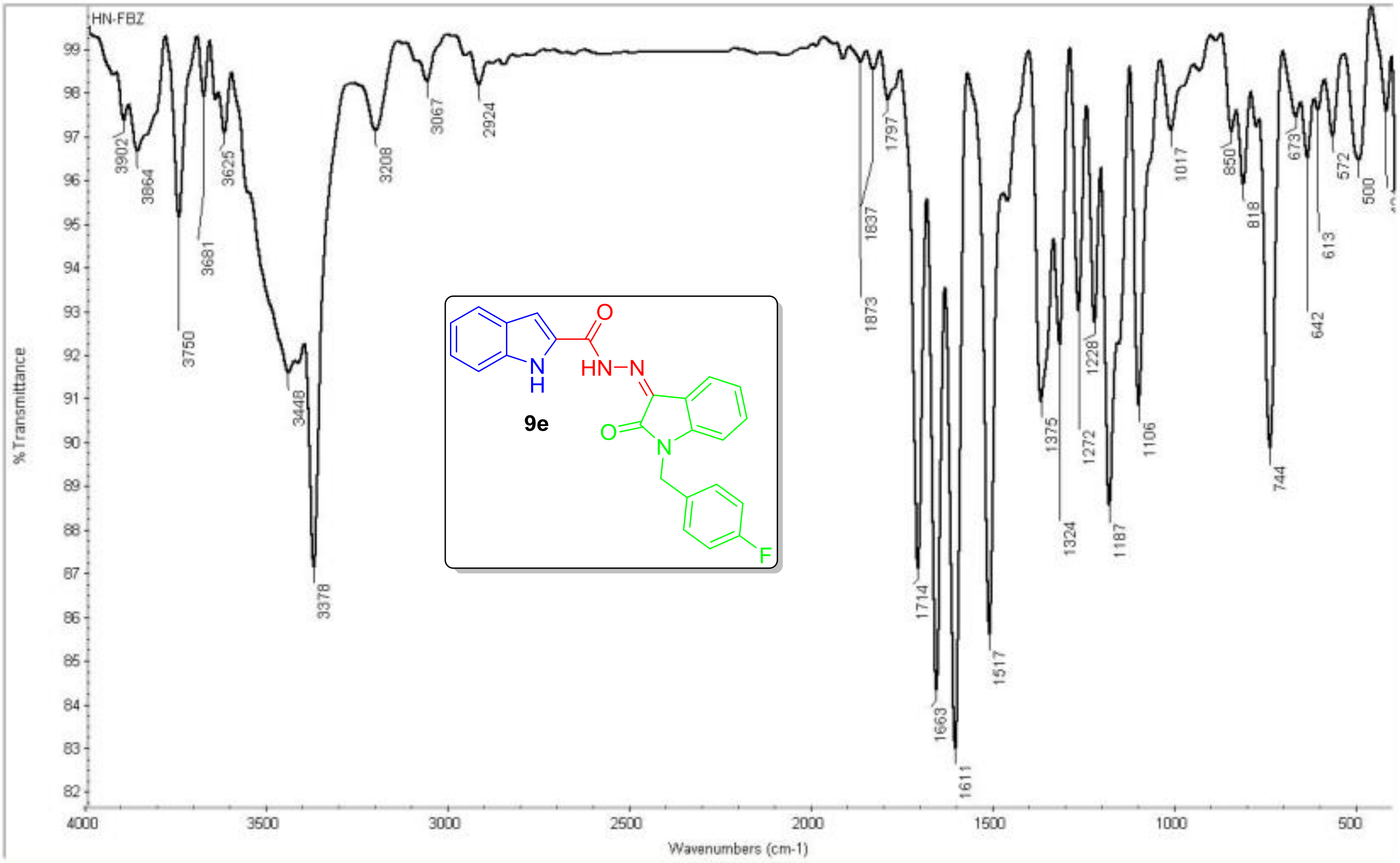
HN-FBZ
% Transmittance
Wavenumbers (cm-1)
99
98
97
96
95
94
93
92
91
90
89
88
87
86
85
84
83
82
4000
3500
3000
2500
2000
1500
1000
500
3902
3864
3750
3681
3625
3448
3378
3208
3067
2924
1873
1837
1797
1714
1663
1611
1517
1375
1324
1272
1228
1187
1106
1017
850
818
744
673
642
613
572
500
9e
